# How Migration Status Shapes Susceptibility of Individuals’ Loneliness to Social Isolation

**DOI:** 10.3389/ijph.2022.1604576

**Published:** 2022-12-06

**Authors:** Lea-Maria Löbel, Hannes Kröger, Ana Nanette Tibubos

**Affiliations:** ^1^ German Institute for Economic Research (DIW), Berlin, Germany; ^2^ Berlin Graduate School of Social Sciences, Humboldt University of Berlin, Berlin, Germany; ^3^ Department of Nursing Science, University of Trier, Trier, Germany

**Keywords:** social isolation, loneliness, mental health, migration, refugees

## Abstract

**Objectives:** Our research provides competing hypotheses and empirical evidence how associations between objectively social isolation and subjective loneliness differ between host populations, migrants, and refugees.

**Methods:** The analysis uses data of 25,171 participants from a random sample of the German population (SOEP v.35). We estimate regression models for the host population, migrants, and refugees and test five hypotheses on the association between social isolation and loneliness using a Bayesian approach in a multiverse framework.

**Results:** We find the strongest relative support for an increased need for social inclusion among refugees, indicated by a higher Bayes factor compared to the hosts and migrants. However, all theoretically developed hypotheses perform poorly in explaining the major pattern in our data: The association of social isolation and loneliness is persistently lower for migrants (0.15 SD−0.29 SD), with similar sizes of associations for refugees and the host population (0.38 SD−0.67 SD).

**Conclusion:** The migration history must be actively considered in health service provision and support programs to better cater to the needs of the different groups.

## Introduction

Loneliness has been called a pandemic of modern times [1, 2], constituting a severe problem for modern societies. It has been associated with an increased risk of developing mental health problems [3–5] and can exacerbate existing vulnerabilities to other health outcomes [6]. Several societal trends have been proposed as explanations for increased risks of loneliness in recent decades, among them global mobility [7]. In 2019, 272 million migrants lived outside of their home country [8]. At the same time, UNHCR counted 25.9 million refugees, 41 million internally displaced persons, and another 3.5 million asylum seekers [9].

Loneliness is defined as the subjective feeling of disconnection from social interactions in everyday life [10]. It is the cognitive evaluation of the objective absence of social networks and support. The objective aspect of the definition of loneliness, absence of a social support network, is often referred to as social isolation [11]. Although social isolation and loneliness are often used interchangeably [12], they are not identical [13]. While social isolation does not *per se* invoke feelings of loneliness [11], it is an important predictor of loneliness throughout the entire life [14, 15]. It is therefore important to know under which conditions social isolation works as a strong predictor for loneliness and hence identify more susceptible groups. Following Diderichsen et al., we use the term differential exposure denoting the differences in incidences of social isolation and loneliness among the groups under investigation. Further, we refer to the differences in the strength of the association of social isolation with loneliness between the groups as *differential susceptibility to social isolation* [16].

Migrants are more often at risk of greater exposure to social isolation than host populations, as their networks in the new environment need to be (re-) established [17, 18]. Additionally, they are prone to experiencing higher rates of loneliness due to cultural differences and language barriers [19]. Whether comparable trends to exposure to social isolation and loneliness exist for refugees has yet to be established. In the current study, we not only study these groups’ risks of suffering from objective and subjective social network deprivation: The economic, legal, and social differences in context motivate our investigation of the question whether—under the condition of social isolation—migrants, refugees, and the host population exhibit differing susceptibilities to social isolation. Answering this question can guide interventions and their prioritization in this area.

We propose competing hypotheses for differential susceptibility to social isolation. These hypotheses imply that host population, migrants, and refugees differ in reaction to the lack of social networks and support given their different circumstances (Table 1). Loneliness is often referred to as an evolutionary warning signal of the body, indicating deviations from a norm of social interaction. Hence, susceptibility to social isolation should not differ substantively based on context [20, 21]. Yet, as we propose context moderation, we expect the maximum difference between the associations among the host population, migrants, and refugees to be above a substantive threshold—in **H**_**1,**_ 0.20 standard deviations (for a more detailed discussion of how we selected this value, see Supplementary Material S4).

**TABLE 1:** Hypotheses on different degrees of vulnerability to social isolation with respect to loneliness. Random sample of the host population, migrants and refugees in Germany, 2016/2017 (SOEP v.35).

Hypothesis	Theory	Proposal		
H_1_—contextual relevance	Context moderation	$\max\left(\left.{\left|\beta \right.}_{h}-\beta_{m}\left|,{\left|\beta \right.}_{h}-\beta_{r}\right|,{\left|\beta \right.}_{r}-\beta_{m}\right|\right)>tt=0.2\;SD$		Substantive differences in the way social isolation correlates with loneliness across host, migrant, and refugee population
		**Ranking**	**Prior Probability^a^**		
			
H_2a_—increased need	Varying need for networks and support: hence elevated vulnerability according to migration experience	$\beta_{h}<\beta_{m}<\beta_{r}$	$\frac{1}{6}$	Host population is hypothesized to experience the least, refugee population the most vulnerability
H_2b_—refugee exceptionalism	Exceptional vulnerability due to exceptional strain among refugees	${\left\{\beta \right.}_{h},\beta_{m}\}<\beta_{r}$	$\frac{2}{6}$	Refugees are exceptionally vulnerable to social isolation, but there are no systematic differences between host and migrant population
H_2c_—numbing	Trauma resulting in emotional unresponsiveness	${\left\{\beta \right.}_{h},\beta_{m}\}>\beta_{r}$	$\frac{2}{6}$	Lesser association between social isolation and loneliness experienced by refugees compared to the host and migrant population
H_2d_—anticipation	Self-selection into migration yields lower susceptibility	$\beta_{h}>\beta_{m}>\beta_{r}$	$\frac{1}{6}$	Refugees experience the least susceptibility to social isolation, migrants less than host population
H_3_—No systematic ordering		$\left.{\left\{\beta \right.}_{h},\beta_{m},\beta_{r}\right\}$	1	No systematic ordering

The β indicates the association between social isolation and loneliness. Population groups are defined in the index: h = host, m = migrant, r = refugee.

H_3_: “no systematic difference,” in contrast to conventional hypothesis terminology in frequentist statistics. We believe that the association will never be exactly equal. Therefore, we also establish hypothesis H_3_ instead of H_0_.

a See methods section and Supplementary Material for a derivation of the prior probabilities.

**H**_**2**_ advances a set of competing rankings of differential susceptibility between the host population, migrants, and refugees, founded in migration context and experiences: In detail, **H**_**2a**_ proposes that the highest need for social support and inclusion exists for refugees and to a lesser extent for migrants. Their circumstances make them particularly susceptible to social isolation [22]. **H**_**2b**_ hypothesizes an exceptional susceptibility in refugees, evinced in an elevated association between social isolation and loneliness compared to migrants and the host population [23]. **H**_**2c**_ expects an emotionally numb response among refugees resulting from traumatic displacement experiences. This would lead to a smaller association between social isolation and loneliness compared to the levels of sensibility in the host and migrant population [24, 25]. **H**_**2d**_ postulates that social isolation is anticipated by migrants, and even more so by refugees, through self-selection into migration, reducing their susceptibility to social isolation [26, 27]. **H**_**3**_ is the baseline hypothesis in this analysis, suggesting no systematic differences in susceptibility between groups.

We use one of the few available data sets which includes comparable and harmonized data for refugees, migrants, and a host population: the German Socio-Economic Panel Study (SOEP, v.35, *N* = 25,171). We use a Bayesian Evaluation of Informative Hypotheses (BEIH) framework to evaluate the hypotheses [28, 29], testing the robustness of our results in a multiverse framework [30, 31].

## Methods

### Study Population

We use 2016 and 2017 data from the SOEP v.35 [32]. The household survey is a stratified random sample of the German population with recent booster samples for migrants and refugees (DOI: 10.5684/soep-core.v35). Notably, the IAB-BAMF-SOEP refugee survey enabling the analysis of many cases of recently arrived refugees to Germany between 2013 and 2016. The SOEP draws its refugee samples from the Central Register of Foreign Nationals. Hence, the refugee survey is a random sample from a clearly defined population. This is a clear advantage, as most refugee surveys are based on highly targeted, clinical, or convenience samples.

### Migration Status

We group individuals into [1] Germans and second-generation migrants–labelled host population (*n* = 16,658) [2], first generation migrants (*n* = 3790) and [3] refugees (or similar protected) who have arrived since 2013 as part of the IAB-BAMF-SOEP survey (*n* = 4723). We define the host population as those born as German nationals or those born in the Federal Republic of Germany as of 1949. Further, we define refugees in Germany as those having applied for asylum, regardless of the outcome of their application.

### Loneliness

We use the three-item version of the UCLA loneliness scale as our measure for subjective loneliness [33]. Items are rated on 5-point scales (0 = “never”, 1 = “rarely”, 2 = “sometimes”, 3 = “often”, 4 = “very often”). Based on this scale, we test two different outcome measures of loneliness [1]: a simple summary score and [2] the factor score from a confirmatory factor analysis of the three items. Migrants and host population were surveyed on the three items in 2017, refugees in 2016, leading us to transmit the 2016 information to 2017. Measurement invariance tests across groups can be found in the supplementary material (Supplementary Figures S1, S2 and Supplementary Tables S1, S2).

### Social Isolation

We measure social isolation as a composite index, across three domains consisting of several indicators [34]:

1. The size of the support network (SS), measured as the number of individuals given by respondents as social support in three categories. The SOEP reports the social support (SS) for refugees in 2017 and for the host population and other migrants in 2016. Hence, we transmit the 2016 information for SOEP participants to 2017.
2. Living and partnership arrangements (LA): a) having a spouse and b) presence of other household members.
3. Frequency of attending social activities (SA): a) church, b) cultural activities, c) cinema/disco, d) sports, e) arts.

For each indicator and domain, and for the total calculation of domains, we test two alternative cut-off points: one that allows for more and another allowing for less substitution within and between domains (*N* = 16) (see Supplementary Material S3). Substitution refers to the assumption that a lack in one dimension can compensated by another.

### Covariates

We control for age groups and gender as well as education, residency in rural and urban areas, and residency in former East or West Germany (Supplementary Material S2).

### Statistical Analysis

The central parameters that represent the quantities of interest from our hypotheses are the coefficients ( $\left.\beta \right)$ of social isolation in a regression model for three migration groups (for more information see Supplementary Material S7). Moreover, the model is a multilevel model. Individuals are nested within 24 gender-specific age groups. This multilevel setup allows for a post-stratification procedure, accounting for the possibility that differences in the association found in the data could be attributed to the strong differences in the age and gender composition of the groups (for more information see Supplementary Material S7).

We use the BEIH framework (see Supplementary Material S8), designed for a comparative evaluation of competing hypotheses (H_t_) [35]. The general estimation procedure used for the posterior distribution of the parameters is the Integrated Nested Laplace Approximation (INLA) [36]. The key feature of the BEIH method is to compare the *observed* support $p\left(H_{t}|s\left(Y\right)\right)$ for the hypothesis from the estimated posterior distribution of the coefficients to the *expected* support $p\left(H_{t}\right)$ for the hypothesis (prior probability). The prior probability is calculated assuming random ordering of the coefficients (Table 1) [37]. If the retrieved Bayes factor is larger than one, the hypothesis formulated has more predictive power than given by chance. Posterior model probabilities (PMP) state how much support one hypothesis receives compared to the overall support that all hypotheses under investigation receive [29].

Recent research proposes that studies based on secondary data report all plausible specifications of their data coding and sample definitions [30, 31]. This reduces the probability of reporting findings specific to certain idiosyncratic decisions in the process of the data analysis [38]. Based on the definition of social isolation and the different cut-off points presented, in addition to alterations in sample definition and coding, we report all plausible specifications or codings in a multiverse framework (Supplementary Material S3–S5).

## Results

Descriptive statistics of loneliness are provided in Table 2. The factor score of loneliness is smallest for the host population (M = -0.17, SD = 0.85), and larger for migrants (M = −0.03, SD = 0.97) and refugees (M = 0.55, SD = 1.24). The magnitude of the differences is even more intuitive when observing the summary score between the groups, ranging from a M = 2.88 (SD = 2.22) in the host population to a M = 4.81 (SD = 3.23) for refugees. The dispersion is larger in the refugee population compared to the host population and migrants. Supplementary Table S7 lays out further descriptive statistics.

**TABLE 2:** Descriptive statistics of the two dependent variables of interest: a factor score of loneliness and the sum score—by subgroup. Random sample of the host population, migrants and refugees in Germany, 2016/2017 (SOEP v.35).

	Hosts				Migrants				Refugees			
	M	SD	Min	Max	M	SD	Min	Max	M	SD	Min	Max
Factor score	−0.17	0.85	−1.28	3.33	0.04	0.97	−1.28	3.33	0.57	1.24	−1.28	3.33
Summary score	2.88	2.22	0.00	12.00	3.42	2.53	0.00	12.00	4.81	3.23	0.00	12.00

M, mean; SD, standard deviation; min, minimum; max, maximum.

**FIGURE 1:**
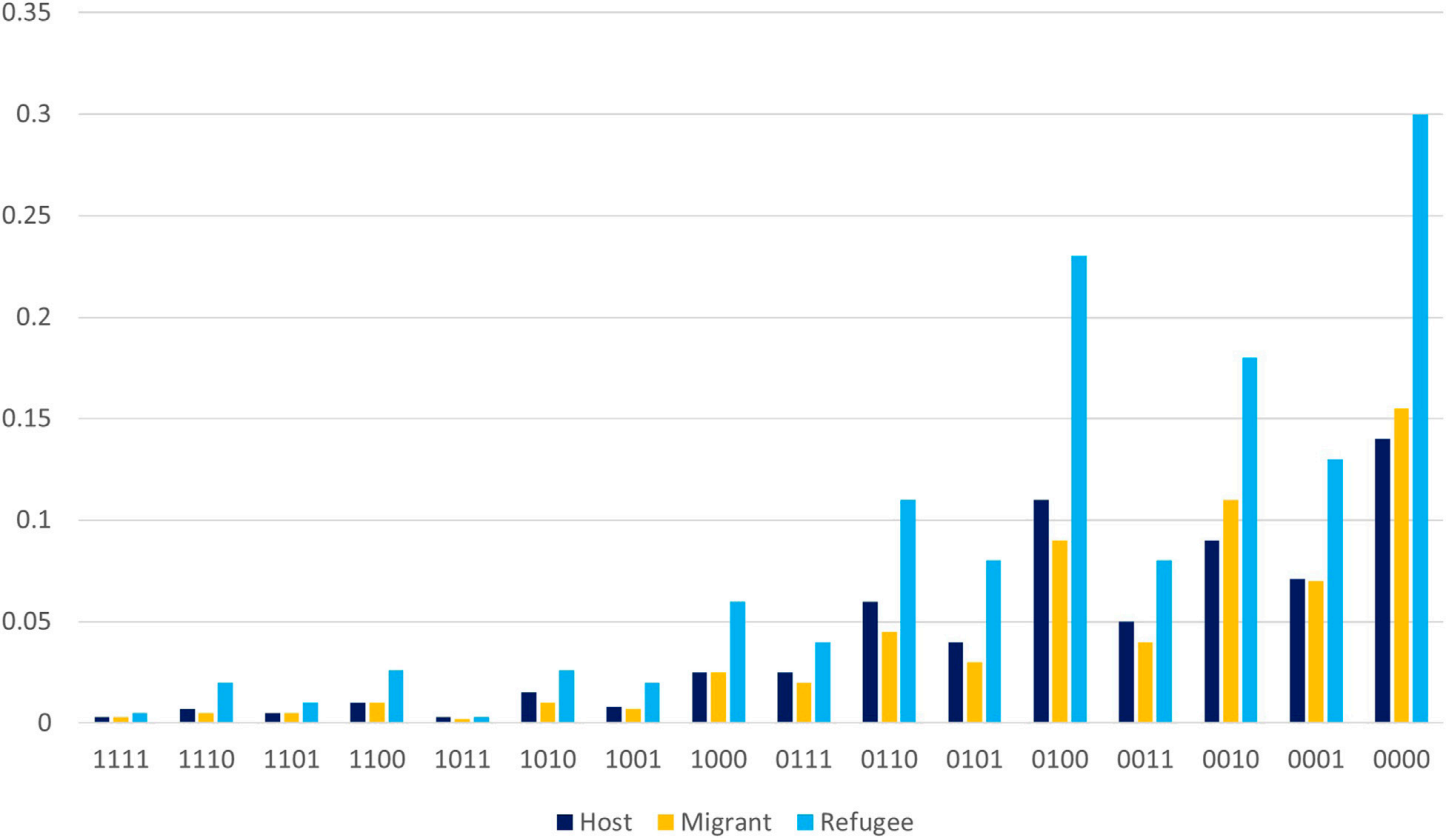
Prevalence of social isolation by migration status over different codings of social isolation. Random sample of the host population, migrants and refugees in Germany, 2016/2017 (SOEP v.35). ^a^X-Axis: Coding choice of the social isolation indicator: The 4 digits for each isolation indicator on the X-axis describe the degree of substitution allowed in each step of the generation of the indicator for social isolation (see equations 1 and 2 in the Supplementary Material S4 (p.18)). Y-Axis: prevalence of social isolation given the choice of the social isolation indicator. ^b^The first digit represents the coding for the degree of substitution across dimensions ( $s_{SI}$ ). The second digit indicates substitutability within the social support ( $s_{SS}$ ) dimension. The third digit indicates substitutability within the living and partnership arrangements ( $s_{LA}$ ) dimension. The fourth digit indicates substitutability within the social activities ( $s_{SA}$ ) dimension. s = 1 stands for full substitutability, s = 0 for partial substitutability.

Notably, the prevalence of social isolation depends on the choice of cut-off points (Figure 1). The less we allow for substitution among and between dimensions, the more people count as socially isolated. Under full substitution (coding 1111), less than 1% of the sample are categorized as socially isolated. When only partial substitution is allowed (coding 0000), social isolation becomes as high as 30% among the group of refugees, and 15% for host population and migrants. Disallowing full substitutability in the domain of social activities most strongly increases the prevalence of social isolation. Overall, refugees are more socially isolated than migrants and the host population. Host population and migrants do not differ greatly regarding the prevalence of social isolation.

**FIGURE 2:**
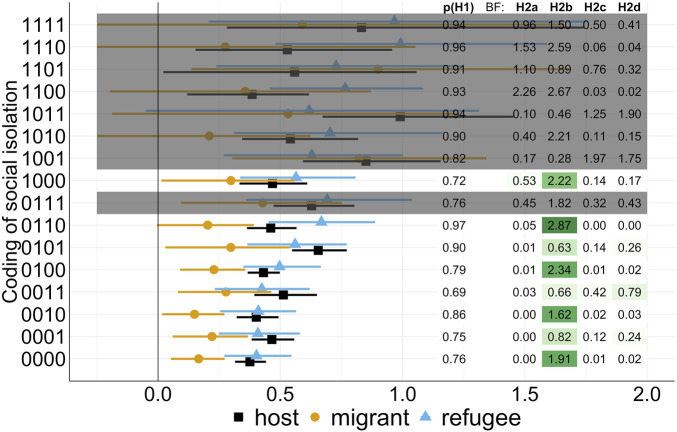
Regression results and relative support for the hypotheses. Random sample of the host population, migrants and refugees in Germany, 2016/2017 (SOEP v.35). ^a^The table compares posterior model probabilities (colour) and Bayes Factor (numerics) to the credible intervals derived from analysis across the different codings of the social isolation indicator. ^b^The X-axis refers to effect sizes while the Y-axis describes different choices of codings of the social isolation indicator in the multiverse analysis (see also Supplementary Figures S4A,B). ^c^The 4 digits of each isolation indicator describe the degree of substitution allowed in each step of the generation of the indicator for social isolation. The first digit represents the coding for the degree of substitution across dimensions ( $s_{SI}$ ). The second digit indicates substitutability within the social support ( $s_{SS}$ ) dimension. The third digit indicates substitutability within the living and partnership arrangements ( $s_{LA}$ ) dimension. The fourth digit indicates substitutability within the social activities ( $s_{SA}$ ) dimension. A s = 1 stands for full substitutability, a s = 0 for partial substitutability. ^d^Results which we highlighted in grey are based on cell sizes for social isolation of less than 50. Bayes factors are provided by the numbers in the table on the right. The strength of the posterior model probability is indicated by green cells in the table. The darker the green, the stronger relative support for specific hypotheses.

Results from the BEIH analysis are presented in Figure 2, containing two sets of results over the 16 codings of social isolation on the Y-axis in terms of SD. On the left, the posterior mean and 95%-credible interval of the regression coefficients of social isolation are plotted (averaged across specifications in Supplementary Figures S7, S8—for a list with detailed posterior means and credible intervals consult Supplementary Table S8). On the right, the table reports the BF and PMP. The darker the box, the higher the PMP for the hypotheses corresponding to the coding of social isolation. We also would like to note the lowest absolute number of observations counted as socially isolated in some of the codings. They naturally lead to a smaller number of cases per cell in the analysis, in some cases with fewer than 50 observations per cell, marked in grey. We consider codings with fewer than 50 cases as unreliable for interpretation and focus on the results from models with sufficient cases of social isolation in all three groups.

Regardless of the coding of social isolation, we see a substantial association of social isolation with loneliness in all three groups (Figure 2, Supplementary Table S8). Still, the posterior mean of the migrant population is persistently lower than that of the host population and that of refugees. It ranges between 0.149 [SI Coding: 0010, 95%-CI: 0.017; 0.271] and 0.298 [SI Coding: 1000, 95%-CI 0.014; 0.558]. The posterior means of hosts and refugees are larger and similar in size, with an effect size of 0.50 SD and ranging from 0.376 [SI Coding: 0000, 95%-CI: 0.315; 0.442] to 0.655 [SI Coding: 0101, 95%-CI: 0.548; 0.772] for the host population and for refugees from 0.403 [SI Coding: 0000, 95%-CI: 0.271; 0.546] to 0.668 [SI Coding: 0110, 95%-CI: 0.452; 0.888]. This means being socially isolated is associated with about a 0.20 SD higher loneliness score for migrants and about a 0.50 SD higher score of loneliness for the host population and refugees in Germany. These comparisons are always made with respect to those individuals who are not socially isolated within their respective group.

The first column from the left of the table in Figure 2 reflects the evaluation of hypothesis $H_{1}$ . It reports the probability of the maximum difference between the three groups in effect size being above the threshold of 0.20 SD [15]. We can see that, with sufficient observations for social isolation, the probability of the absolute differences being substantial is high. It is above 69% in all codings and in many codings above 90%. Our data and model therefore yield strong support for the contextual relevance hypothesis H_1_.

Figure 2 also shows the BF from the evaluation of the competing hypotheses H2_a-d_. The PMP for hypothesis H_2b_ (refugee exceptionalism) is largest compared to the other four hypotheses, as indicated by the darker green background of the BFs. The BF, however, remains below one in three of the codings with sufficient variation on the independent variable of interest. Hence, despite being favored relative to other hypotheses, it is less supported by our data than would be expected by chance alone, which is a poor absolute performance.

In the other cases, H_2b_ receives the highest BF, with 2.89 times more support for the hypothesis than expected by chance in the SI Coding: 0110 (Figure 2). Hence, we find most support for the hypothesis that the association between social isolation and loneliness is larger for refugees than for the host population and migrants. H2_b_, the increased need hypothesis, comes closest to the data pattern.

This result of the evaluation is at odds with the observations of posterior means and the group level credible intervals. A focus on this output shows that the association between social isolation and loneliness is lower for migrants and about equal for refugees and the host population across different social isolation codings. This phenomenon is an indication that we yet have not correctly identified the most suitable hypothesis given the data.

## Discussion

We hypothesized migration status shaping the susceptibility to social isolation. Among the set of hypotheses, we saw that the “refugee exceptionalism” hypothesis H_2b_ received most support relative to the other hypotheses in the data. The finding that the association between social isolation and loneliness is, overall, weaker for migrants than for the host and refugee groups conflicts with the results of our formal procedure for testing hypotheses. This contradiction indicates that the set of hypotheses did not include the most relevant proposition about the relative strength of association between social isolation and loneliness. The hypothesis that migrants do indeed show a lesser susceptibility to social isolation should be tested in future studies on independent samples.

Looking for an explanation for this finding, we turn to the composition of the migrant group and to alternative hypotheses we equally postulated. The weaker association between social isolation and loneliness for migrants can be attributed to positive self-selection and anticipation (H_2d_). Migrants might more readily accept their social circumstances, as they actively chose their destination based on social network considerations [26]. Meanwhile, refugees have less choice of destination. Their self-selection to migrate is less linked to a well-functioning diaspora than to a need to survive.

One puzzle is the similar pattern of susceptibility to social isolation among refugees and the host population. Deviations from social norms are perhaps more awkward for individuals from the host population, who compare themselves to members of the host community whom they should ostensibly resemble [20]. Meanwhile, a migrant in the same age group is aware of his or her situation and can evaluate it in a positive light. This sensitivity to social deviation from the norm in the host population would then be equal in strength compared to the susceptibility previously postulated for the refugee population.

Overall, refugees show the highest exposure to social isolation and loneliness. Given that they also have a high susceptibility to social isolation, they can be regarded as the most vulnerable group in this analysis, leaving aside their capacity to respond to the exposure [16].

One limitation of our analysis is that data did not allow us to test the underlying mechanisms directly. This also makes it difficult to assess why the data showed a clear but unexpected result in migrants being less susceptible to social isolation. Further research needs to examine the proposed mechanisms separately. We assume the external validity of results, as the prevailing mechanisms should not differ by country. However, the refugee policies of host countries can influence self-selection, for instance through visa sponsorship. Despite the large data set used, a more precise differentiation within the migrant and refugee group is not implemented. It is possible that country of origin, reason for migration, or duration of stay explain how social isolation are linked to loneliness in varying degrees. Last, we argued that social isolation is deprivation in social contacts across domains. While our data cover three domains and several indicators, there are aspects we do not measure, for example, closeness of the social network or integration into transnational networks [39]. More specific data would be necessary for the last two considerations to be further explored.

As established in previous research, loneliness is particularly strongly linked to negative mental health outcomes. This cost to individuals and society should be avoided where possible [2, 13]. Our study suggests that interventions to lessen the risks of mental ill-health can start with the prevention of social isolation. The groups at stake might be best stimulated in different ways in terms of environmental prevention. Nevertheless, the present analysis cannot infer direct policy guidance. Particularly interesting would be a closer examination of whether a substitution effect among different social activities exists. This is just one way forward in studying the association between social isolation and loneliness in the context of migration.

## Editors Note

This manuscript was accepted without revision, after an informal review by one co-editor in chief. For eleven months we were unable to find reviewers. Please read our editorial about the reviewer crisis: https://www.ssph-journal.org/articles/10.3389/phrs.2022.1605407/full.
