# Supplementary material for: How Migration Status Shapes Susceptibility of Individuals’ Loneliness to Social Isolation
Source: Int J Public Health. 2022 Dec 6;67:1604576. doi: 10.3389/ijph.2022.1604576 (PMC9763294; doi:10.3389/ijph.2022.1604576)
Supplement: Supplementary file 1 [file DataSheet2.pdf]

International Journal of Public Health

Supplementary Material II
(online appendix)
It presents the results of all 48 specification curves for all three groups for all 16 codings of social isolation.

How migration status shapes susceptibility of individuals’ loneliness to social isolation

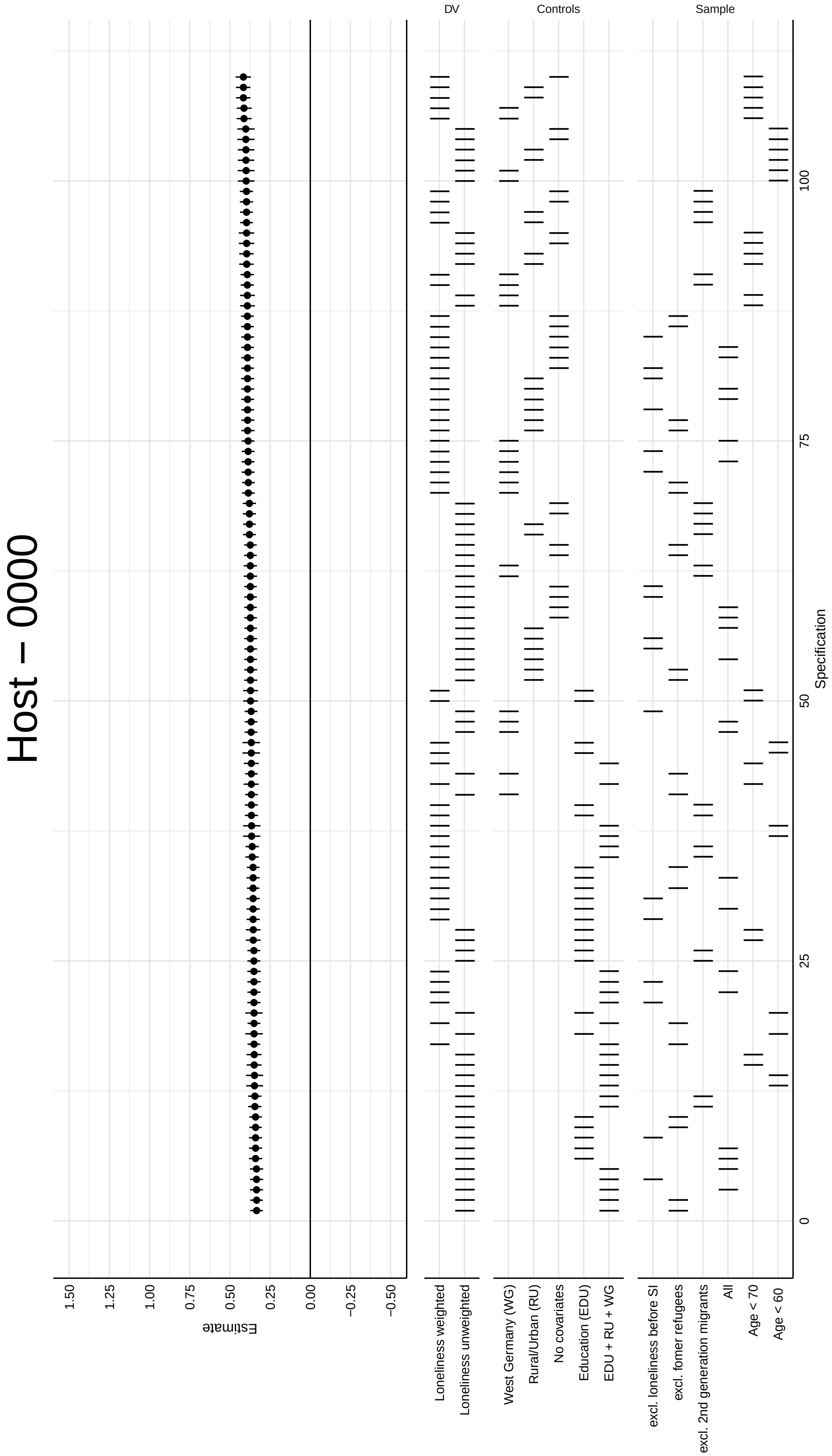
Host – 0000
Estimate
1.50
1.25
1.00
0.75
0.50
0.25
0.00
−0.25
−0.50
DV
Loneliness weighted
Loneliness unweighted
Controls
West Germany (WG)
Rural/Urban (RU)
No covariates
Education (EDU)
EDU + RU + WG
Sample
excl. loneliness before SI
excl. fomer refugees
excl. 2nd generation migrants
All
Age < 70
Age < 60
0
25
50
75
100
Specification

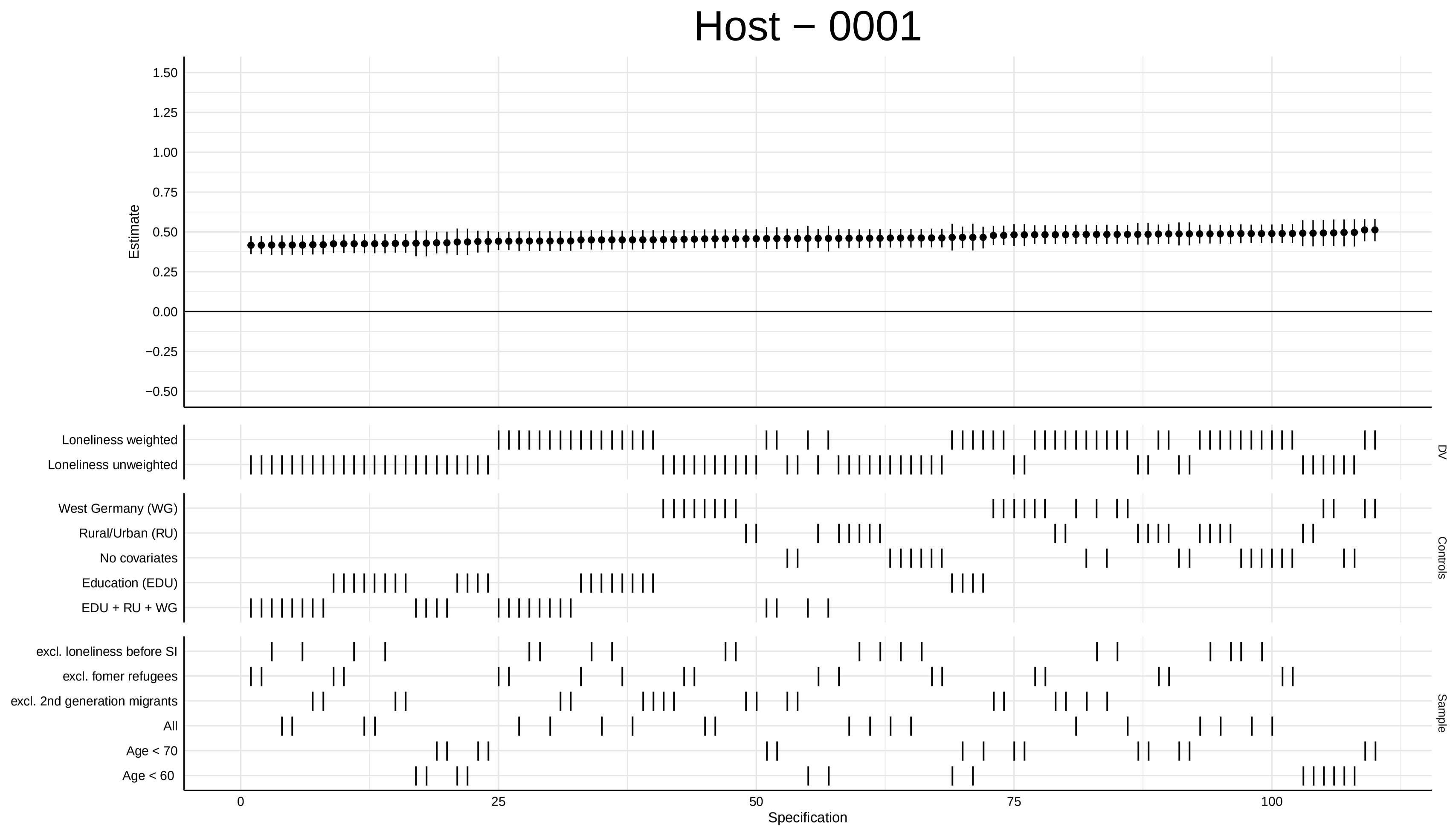
Host − 0001
Estimate
1.50
1.25
1.00
0.75
0.50
0.25
0.00
−0.25
−0.50
Loneliness weighted
Loneliness unweighted
DV
West Germany (WG)
Rural/Urban (RU)
No covariates
Education (EDU)
EDU + RU + WG
Controls
excl. loneliness before SI
excl. fomer refugees
excl. 2nd generation migrants
All
Age < 70
Age < 60
Sample
0
25
50
75
100
Specification

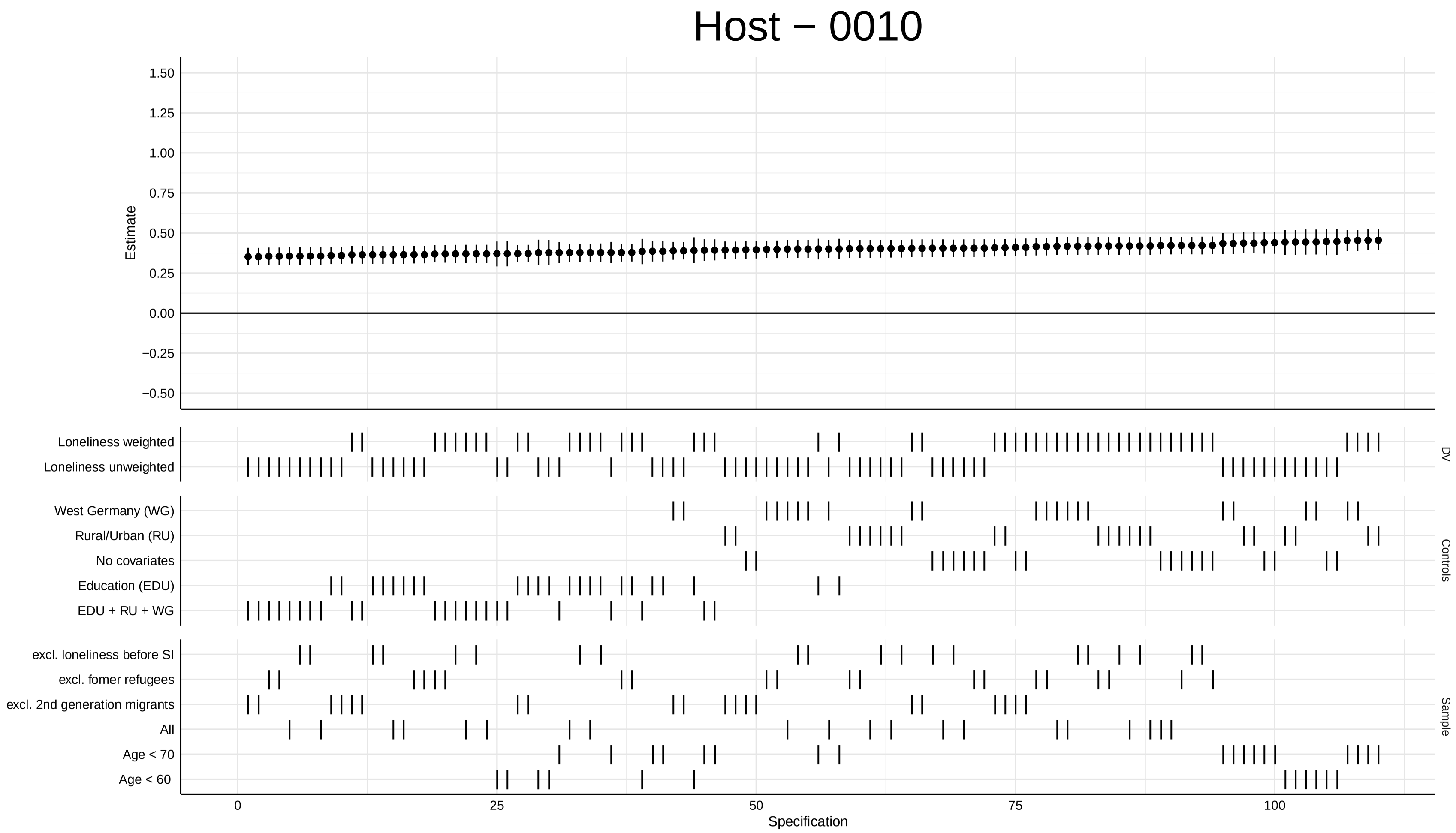
Host − 0010
Estimate
1.50
1.25
1.00
0.75
0.50
0.25
0.00
−0.25
−0.50
Loneliness weighted
Loneliness unweighted
DV
West Germany (WG)
Rural/Urban (RU)
No covariates
Education (EDU)
EDU + RU + WG
Controls
excl. loneliness before SI
excl. fomer refugees
excl. 2nd generation migrants
All
Age < 70
Age < 60
Sample
0
25
50
75
100
Specification

Host – 0011

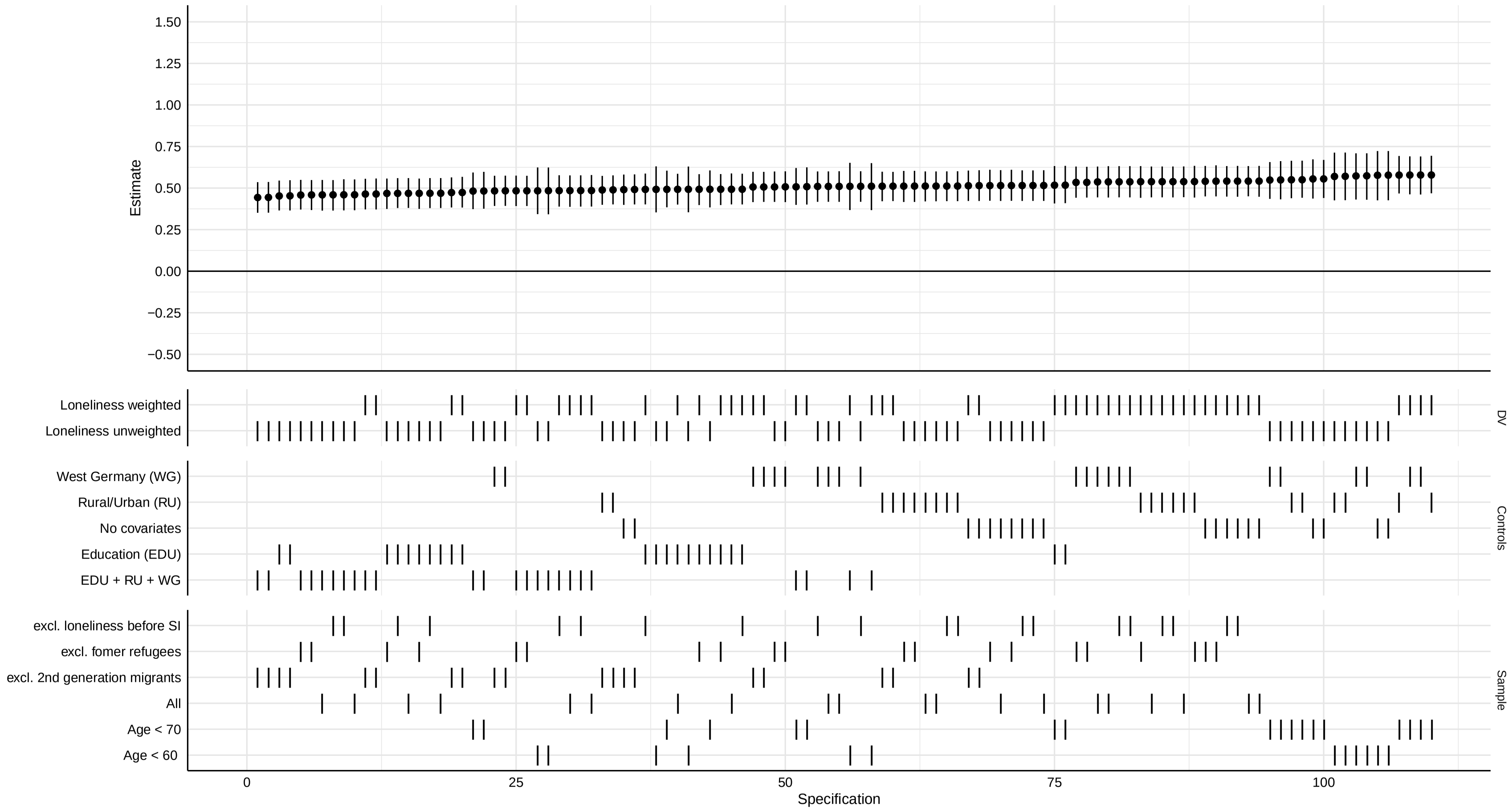

Estimate
1.50
1.25
1.00
0.75
0.50
0.25
0.00
−0.25
−0.50
Loneliness weighted
Loneliness unweighted
DV
West Germany (WG)
Rural/Urban (RU)
No covariates
Education (EDU)
EDU + RU + WG
Controls
excl. loneliness before SI
excl. fomer refugees
excl. 2nd generation migrants
All
Age < 70
Age < 60
Sample
0
25
50
75
100
Specification

Host – 0100

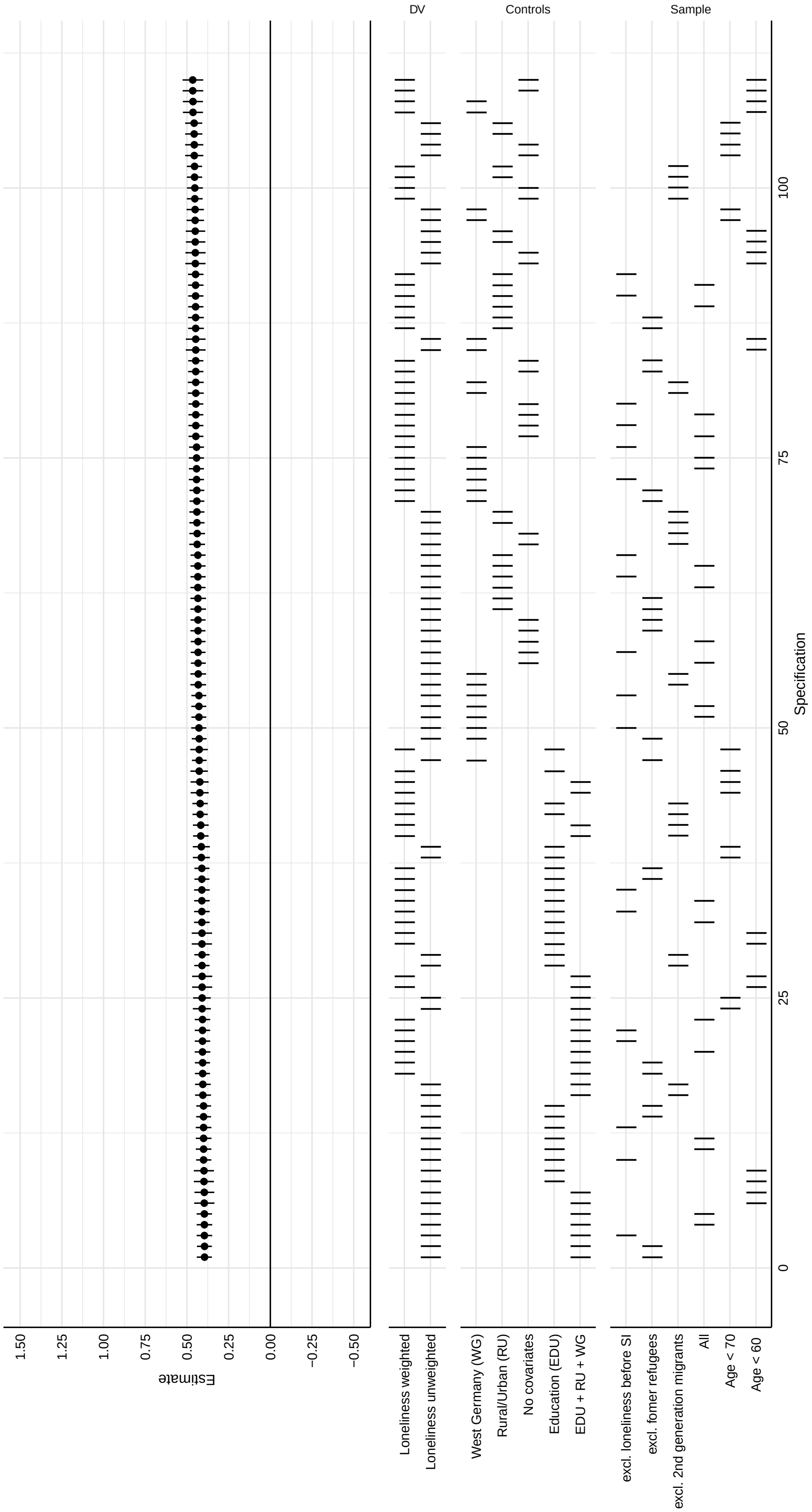

Estimate
1.50
1.25
1.00
0.75
0.50
0.25
0.00
−0.25
−0.50
DV
Loneliness weighted
Loneliness unweighted
Controls
West Germany (WG)
Rural/Urban (RU)
No covariates
Education (EDU)
EDU + RU + WG
Sample
excl. loneliness before SI
excl. fomer refugees
excl. 2nd generation migrants
All
Age < 70
Age < 60
0
25
50
75
100
Specification

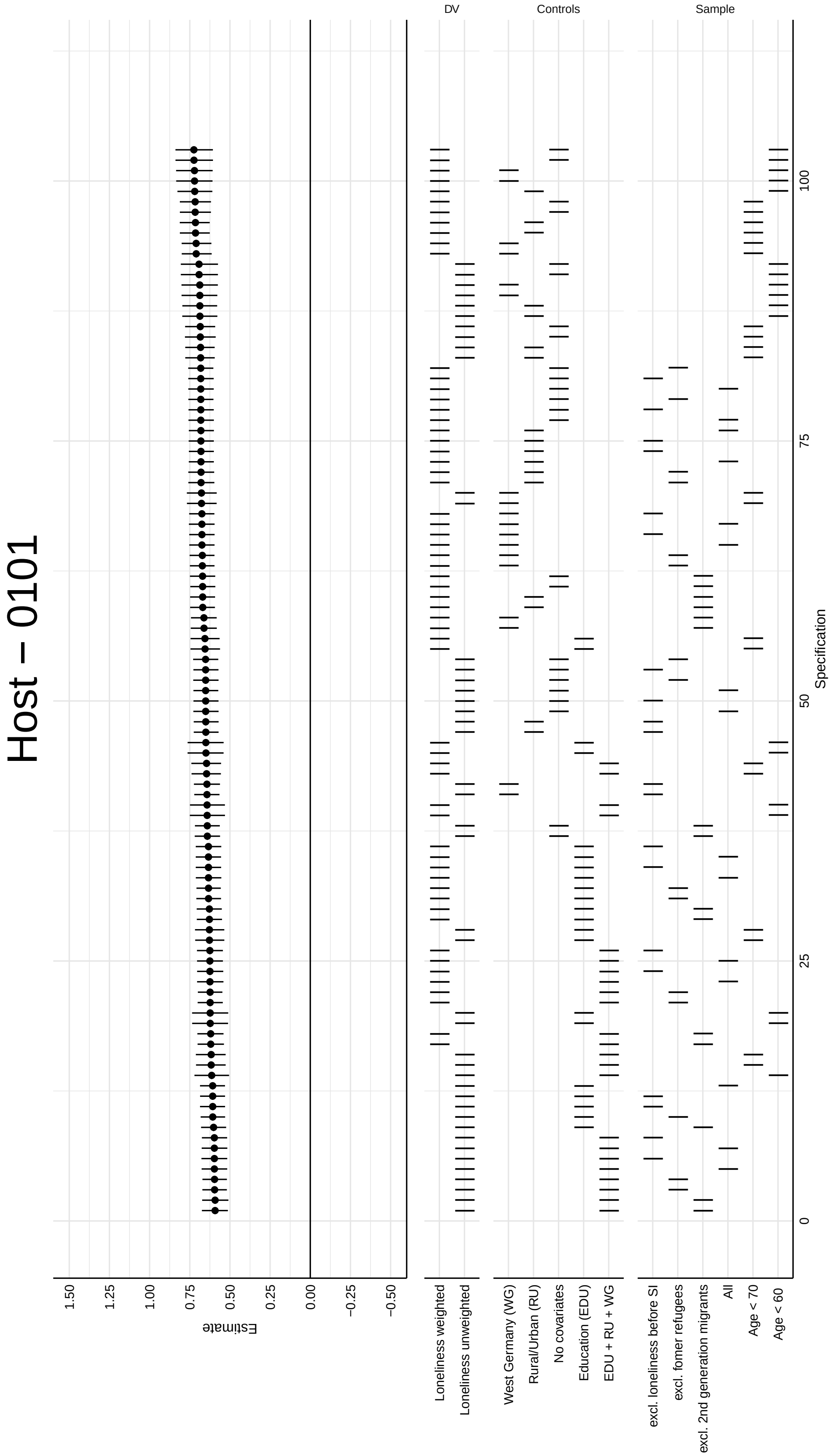
Host – 0101
Estimate
1.50
1.25
1.00
0.75
0.50
0.25
0.00
−0.25
−0.50
DV
Loneliness weighted
Loneliness unweighted
Controls
West Germany (WG)
Rural/Urban (RU)
No covariates
Education (EDU)
EDU + RU + WG
Sample
excl. loneliness before SI
excl. fomer refugees
excl. 2nd generation migrants
All
Age < 70
Age < 60
0
25
50
75
100
Specification

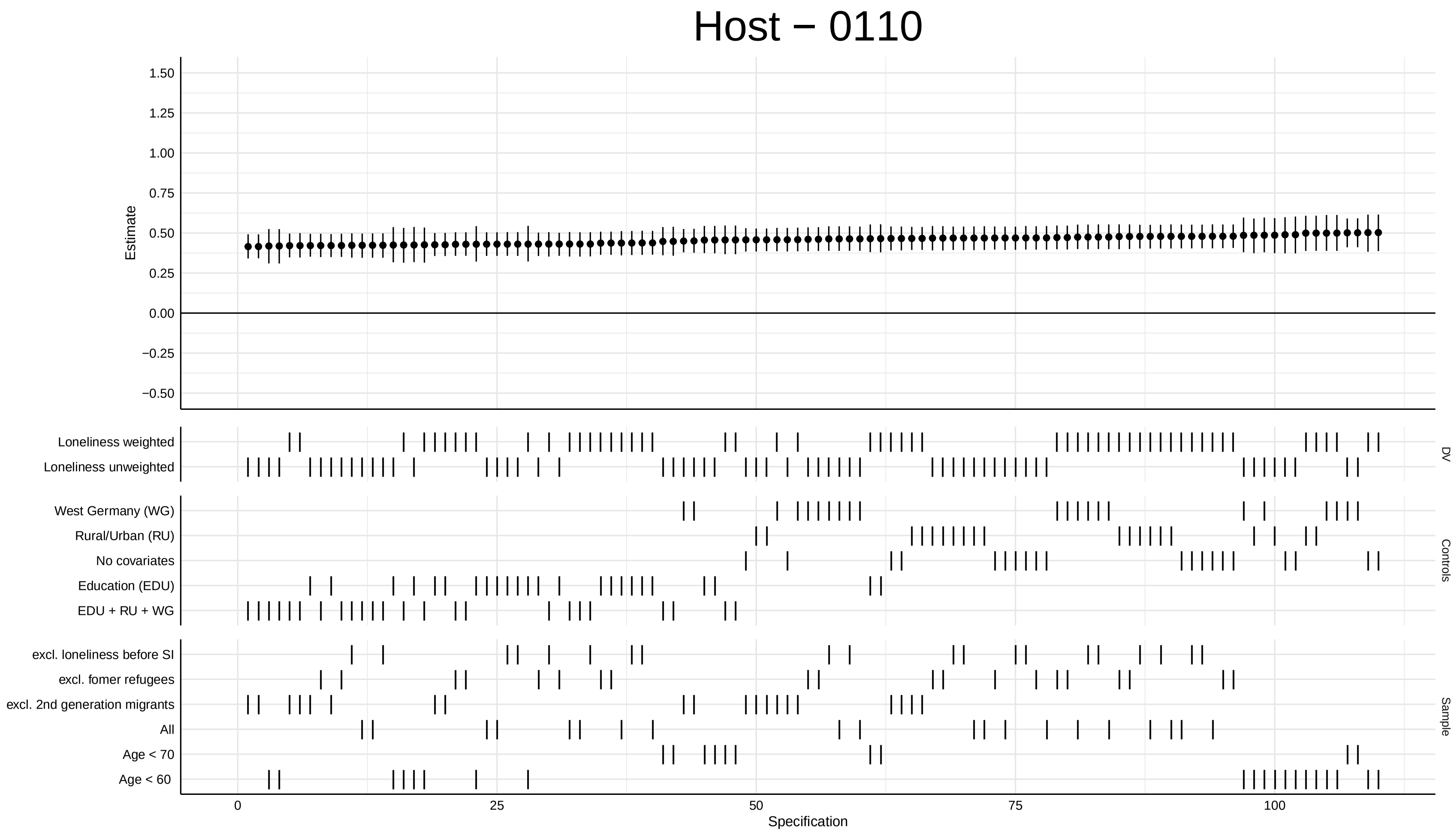
Host − 0110
Estimate
1.50
1.25
1.00
0.75
0.50
0.25
0.00
−0.25
−0.50
Loneliness weighted
Loneliness unweighted
DV
West Germany (WG)
Rural/Urban (RU)
No covariates
Education (EDU)
EDU + RU + WG
Controls
excl. loneliness before SI
excl. fomer refugees
excl. 2nd generation migrants
All
Age < 70
Age < 60
Sample
0
25
50
75
100
Specification

Host – 0111

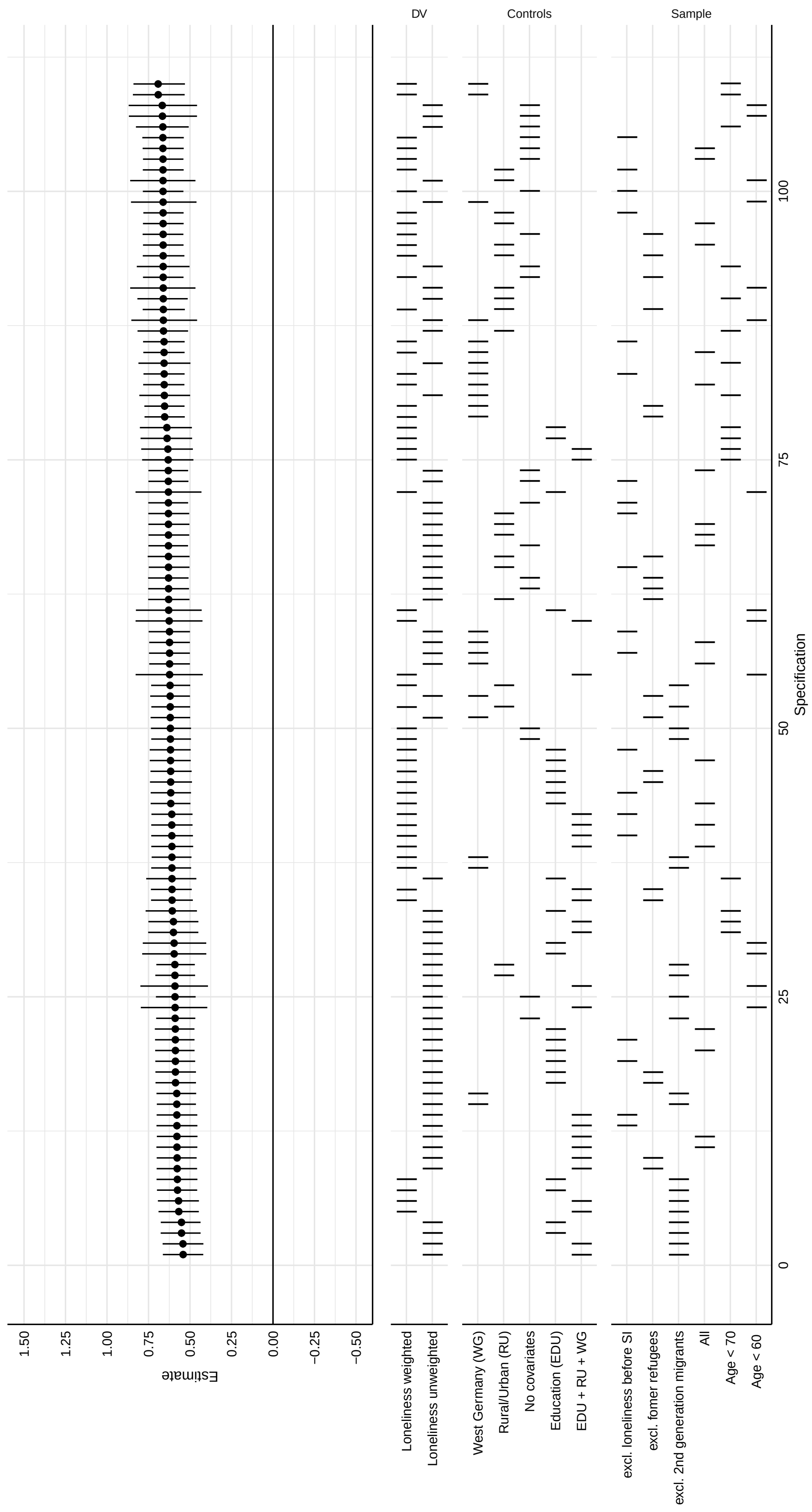

Estimate
1.50
1.25
1.00
0.75
0.50
0.25
0.00
−0.25
−0.50
DV
Loneliness weighted
Loneliness unweighted
Controls
West Germany (WG)
Rural/Urban (RU)
No covariates
Education (EDU)
EDU + RU + WG
Sample
excl. loneliness before SI
excl. fomer refugees
excl. 2nd generation migrants
All
Age < 70
Age < 60
Specification
0
25
50
75
100

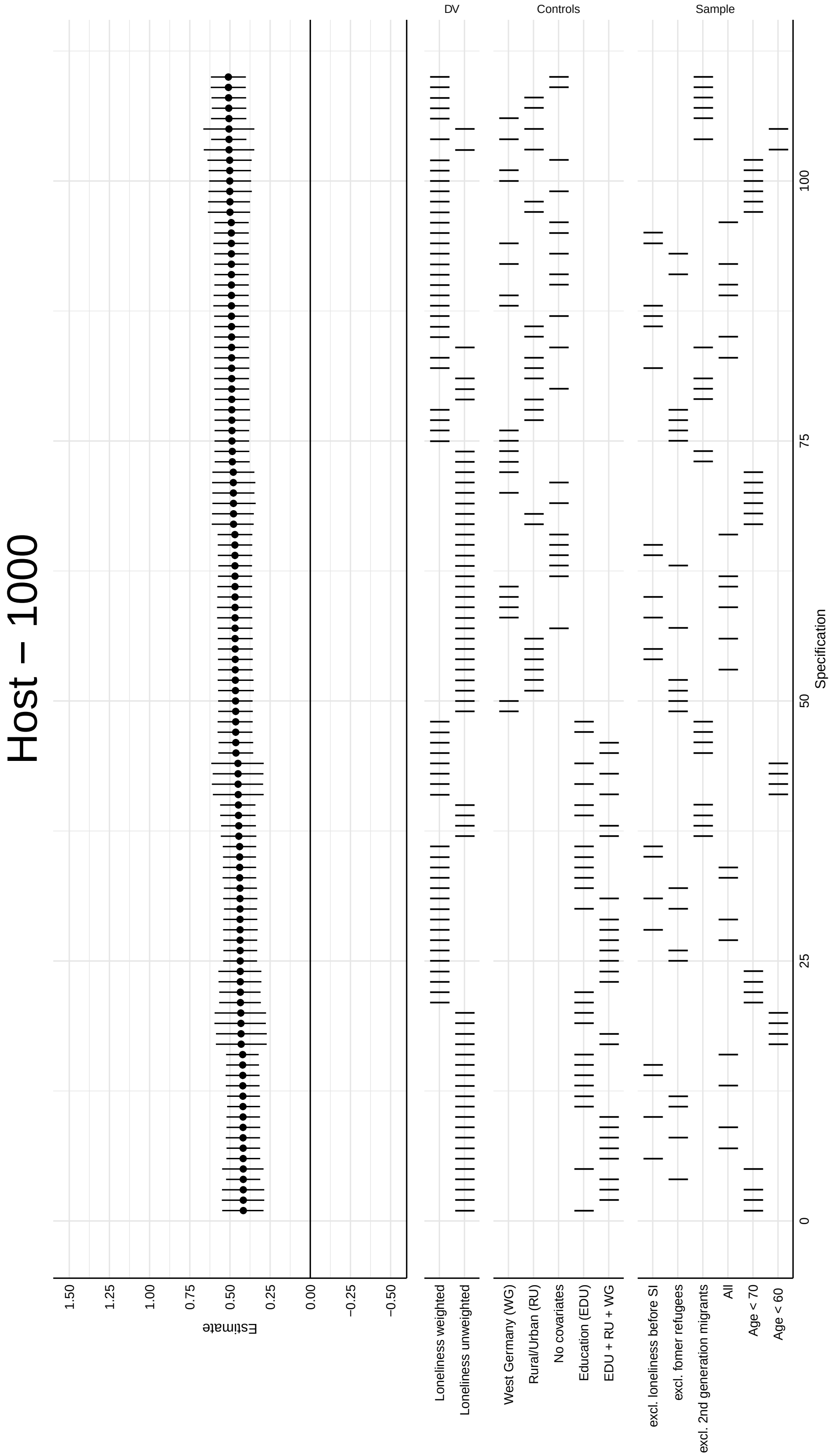

Host – 1000
Estimate
1.50
1.25
1.00
0.75
0.50
0.25
0.00
−0.25
−0.50
DV
Loneliness weighted
Loneliness unweighted
Controls
West Germany (WG)
Rural/Urban (RU)
No covariates
Education (EDU)
EDU + RU + WG
Sample
excl. loneliness before SI
excl. fomer refugees
excl. 2nd generation migrants
All
Age < 70
Age < 60
0
25
50
75
100
Specification

Host – 1001

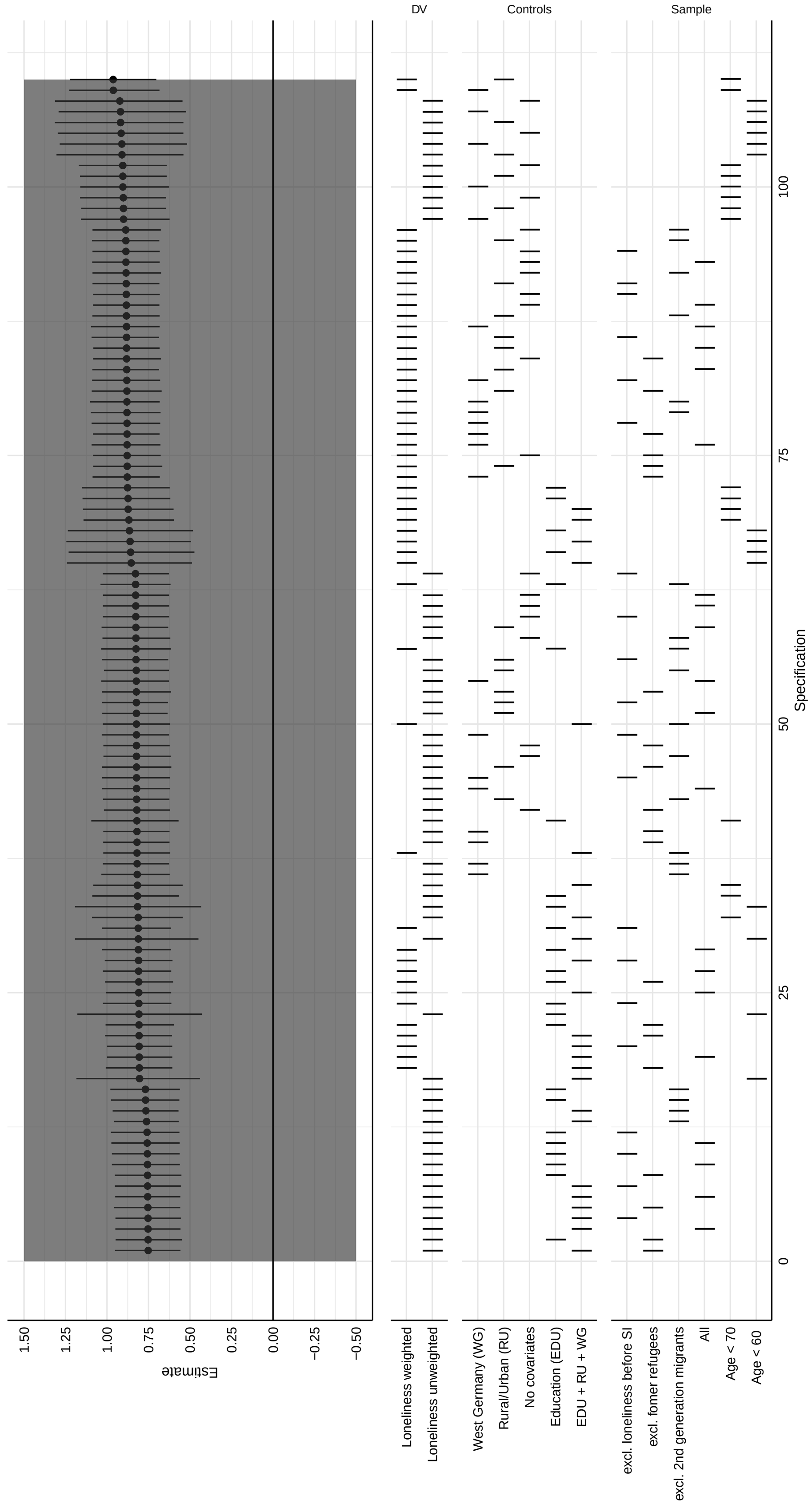

DV
Controls
Sample
Estimate
1.50
1.25
1.00
0.75
0.50
0.25
0.00
−0.25
−0.50
Loneliness weighted
Loneliness unweighted
West Germany (WG)
Rural/Urban (RU)
No covariates
Education (EDU)
EDU + RU + WG
excl. loneliness before SI
excl. fomer refugees
excl. 2nd generation migrants
All
Age < 70
Age < 60
Specification
0
25
50
75
100

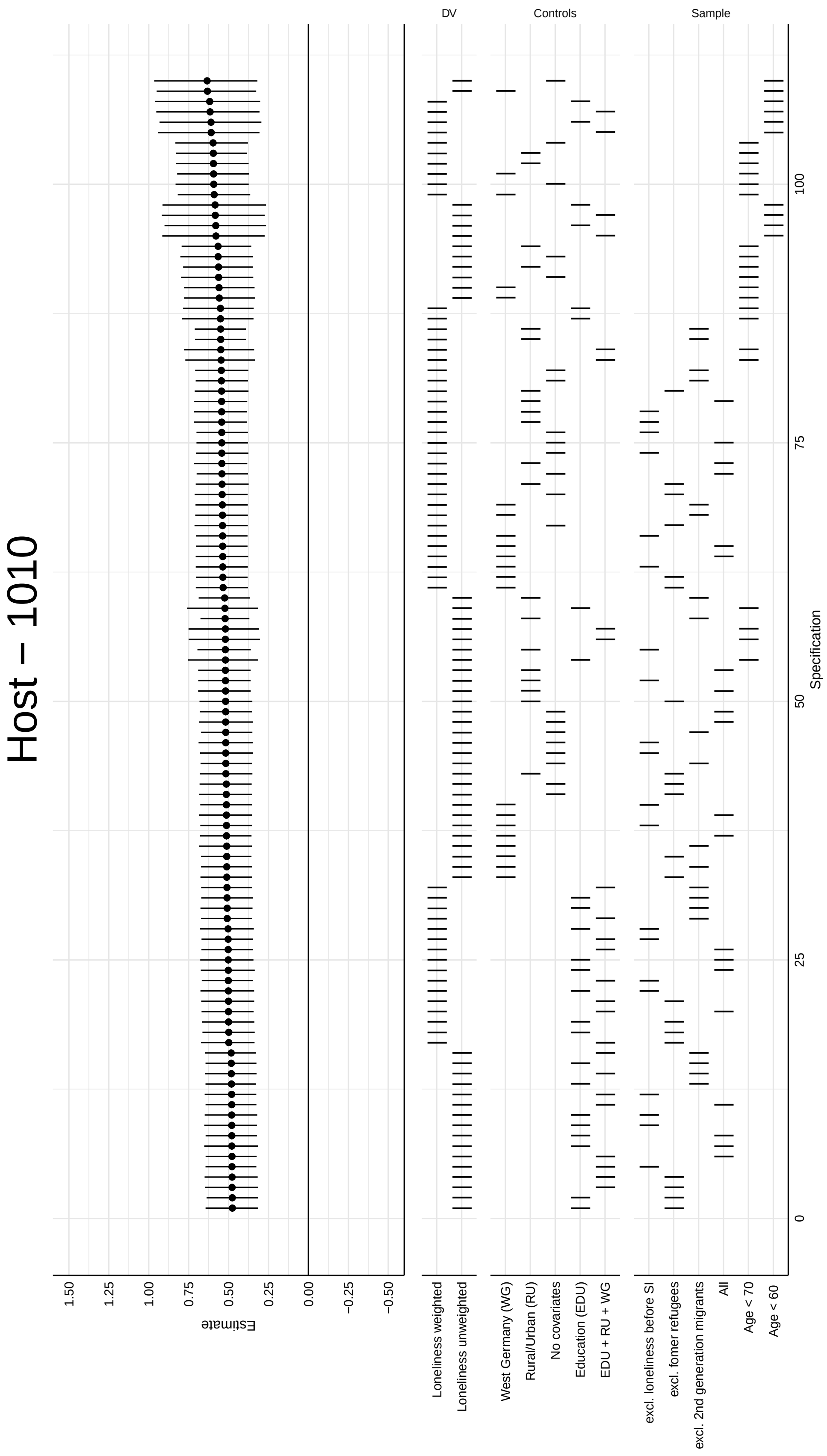
Host – 1010
Estimate
1.50
1.25
1.00
0.75
0.50
0.25
0.00
−0.25
−0.50
DV
Loneliness weighted
Loneliness unweighted
Controls
West Germany (WG)
Rural/Urban (RU)
No covariates
Education (EDU)
EDU + RU + WG
Sample
excl. loneliness before SI
excl. fomer refugees
excl. 2nd generation migrants
All
Age < 70
Age < 60
0
25
50
75
100
Specification

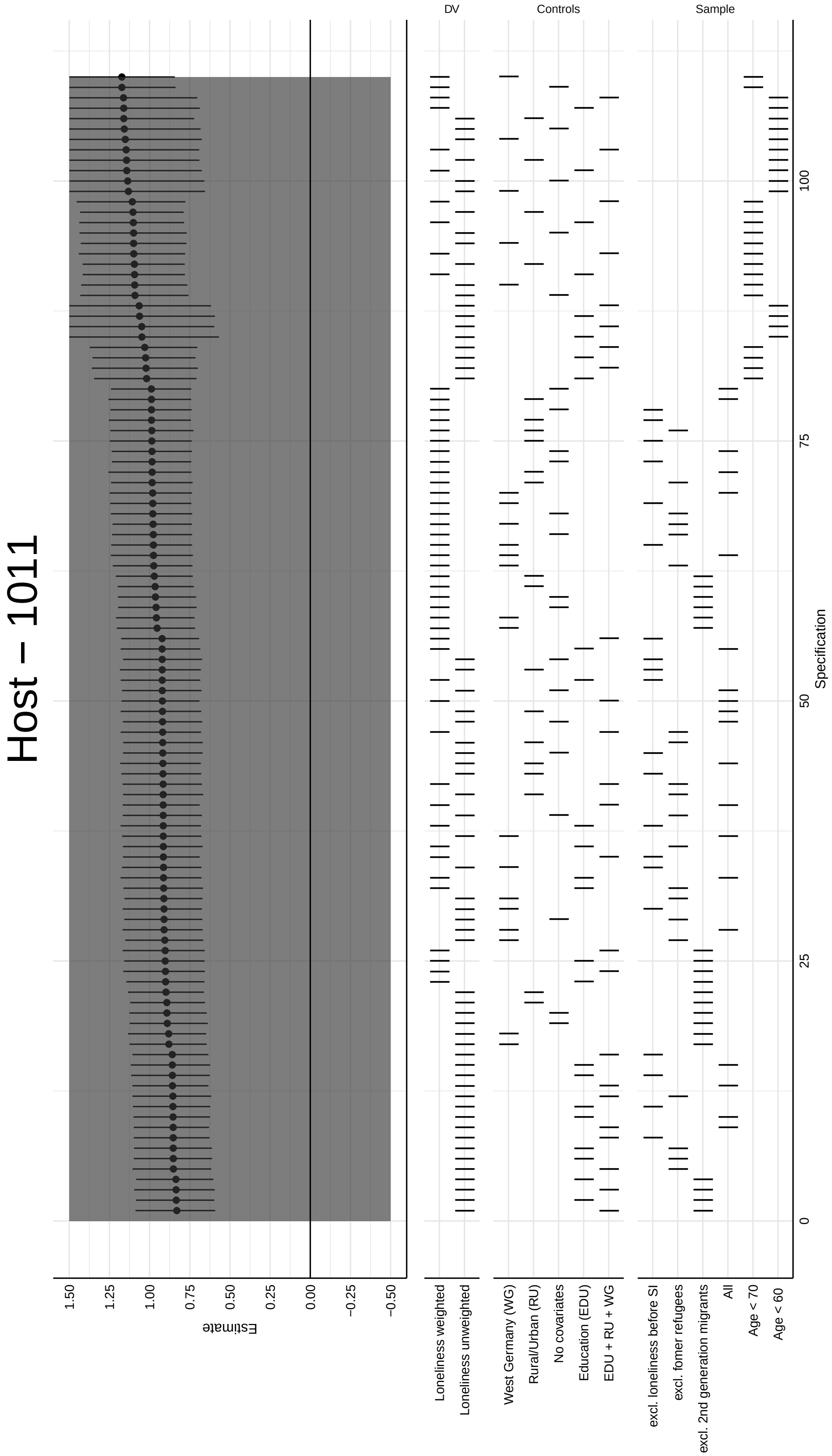
Host – 1011
Estimate
1.50
1.25
1.00
0.75
0.50
0.25
0.00
−0.25
−0.50
DV
Loneliness weighted
Loneliness unweighted
Controls
West Germany (WG)
Rural/Urban (RU)
No covariates
Education (EDU)
EDU + RU + WG
Sample
excl. loneliness before SI
excl. fomer refugees
excl. 2nd generation migrants
All
Age < 70
Age < 60
Specification
0
25
50
75
100

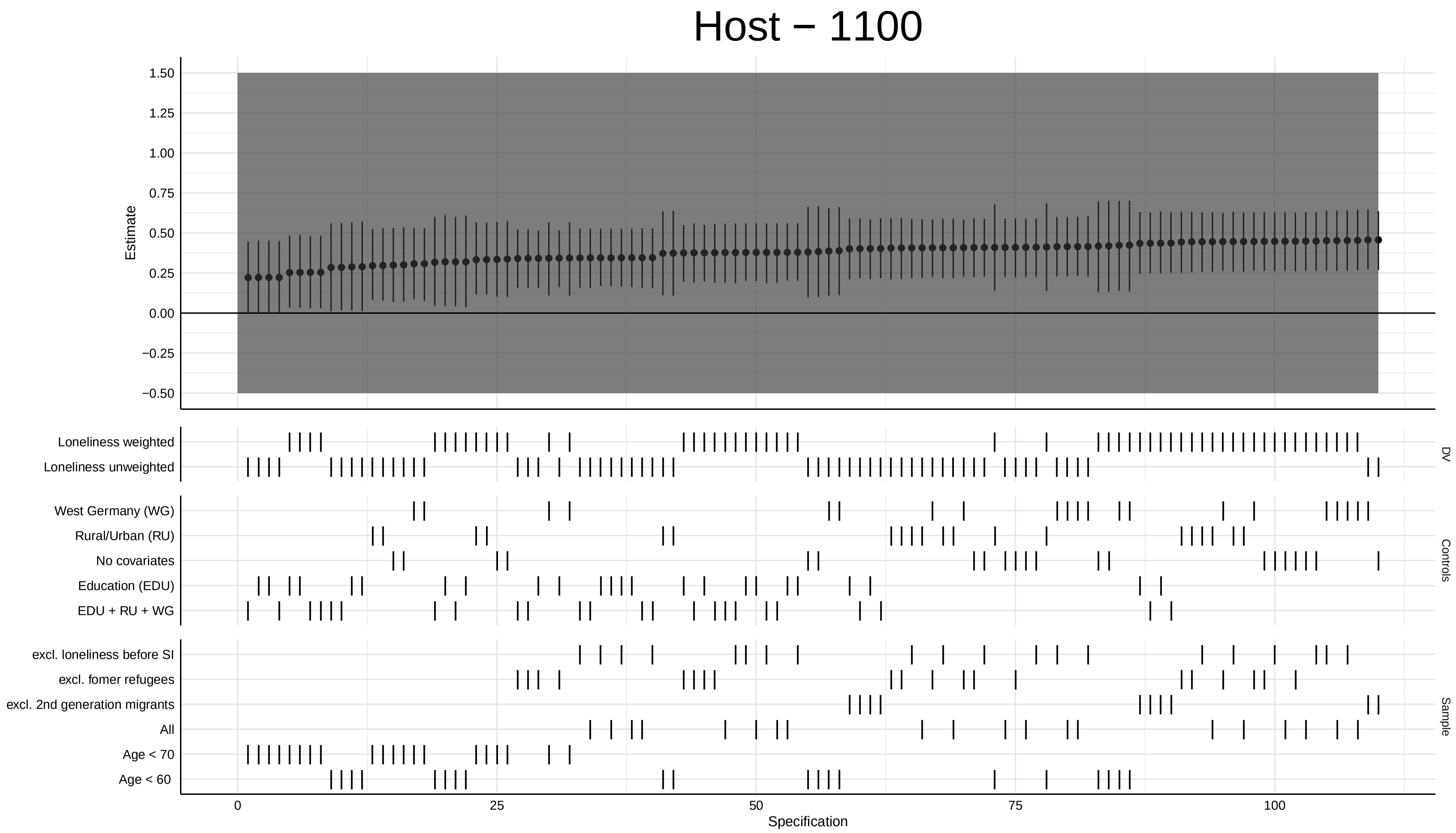
Host − 1100
Estimate
1.50
1.25
1.00
0.75
0.50
0.25
0.00
−0.25
−0.50
DV
Loneliness weighted
Loneliness unweighted
Controls
West Germany (WG)
Rural/Urban (RU)
No covariates
Education (EDU)
EDU + RU + WG
Sample
excl. loneliness before SI
excl. fomer refugees
excl. 2nd generation migrants
All
Age < 70
Age < 60
0
25
50
75
100
Specification

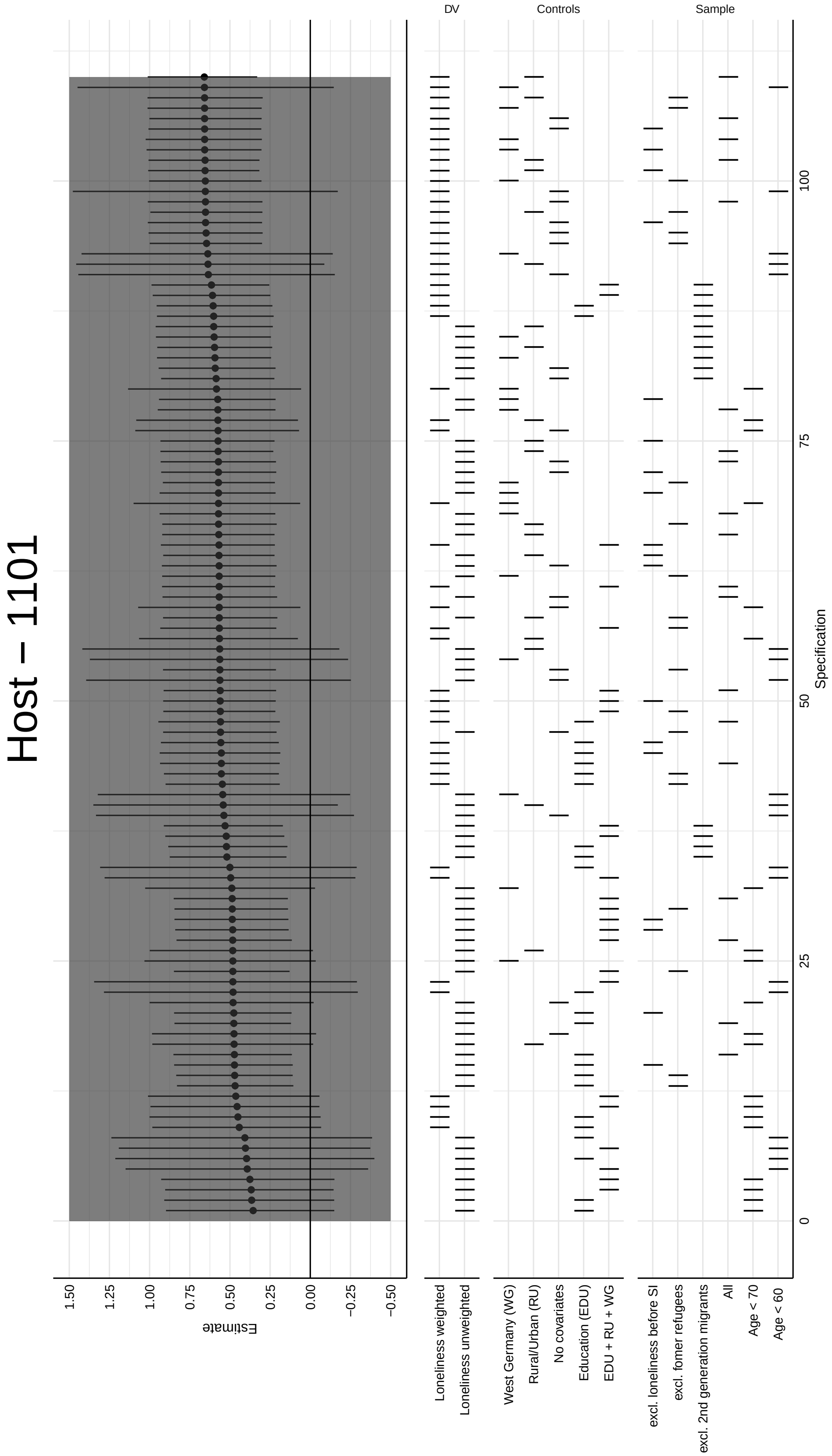

Host – 1101
Estimate
1.50
1.25
1.00
0.75
0.50
0.25
0.00
−0.25
−0.50
DV
Loneliness weighted
Loneliness unweighted
Controls
West Germany (WG)
Rural/Urban (RU)
No covariates
Education (EDU)
EDU + RU + WG
Sample
excl. loneliness before SI
excl. fomer refugees
excl. 2nd generation migrants
All
Age < 70
Age < 60
0
25
50
75
100
Specification

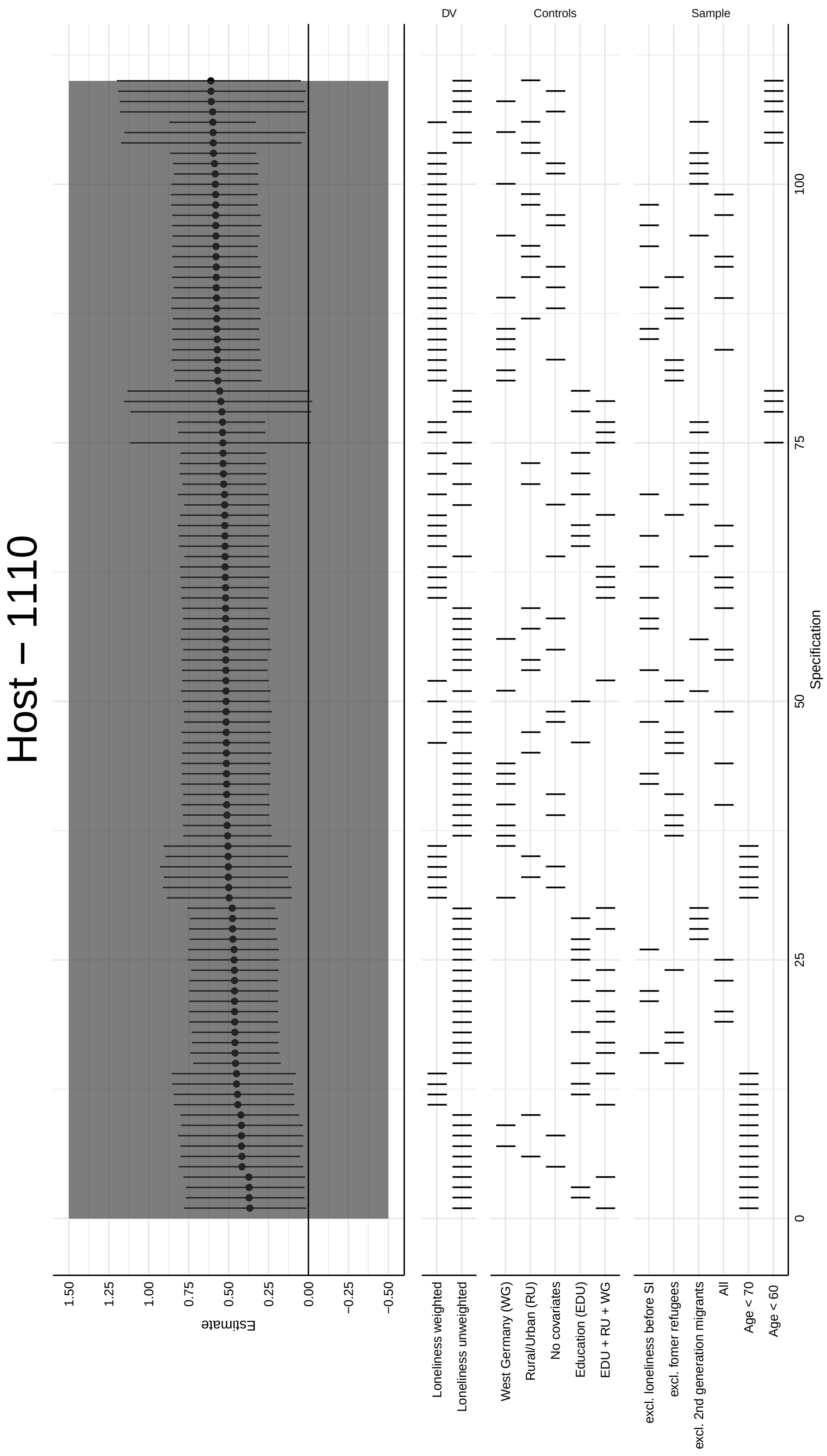

Host – 1110
Estimate
1.50
1.25
1.00
0.75
0.50
0.25
0.00
−0.25
−0.50
DV
Loneliness weighted
Loneliness unweighted
Controls
West Germany (WG)
Rural/Urban (RU)
No covariates
Education (EDU)
EDU + RU + WG
Sample
excl. loneliness before SI
excl. fomer refugees
excl. 2nd generation migrants
All
Age < 70
Age < 60
0
25
50
75
100
Specification

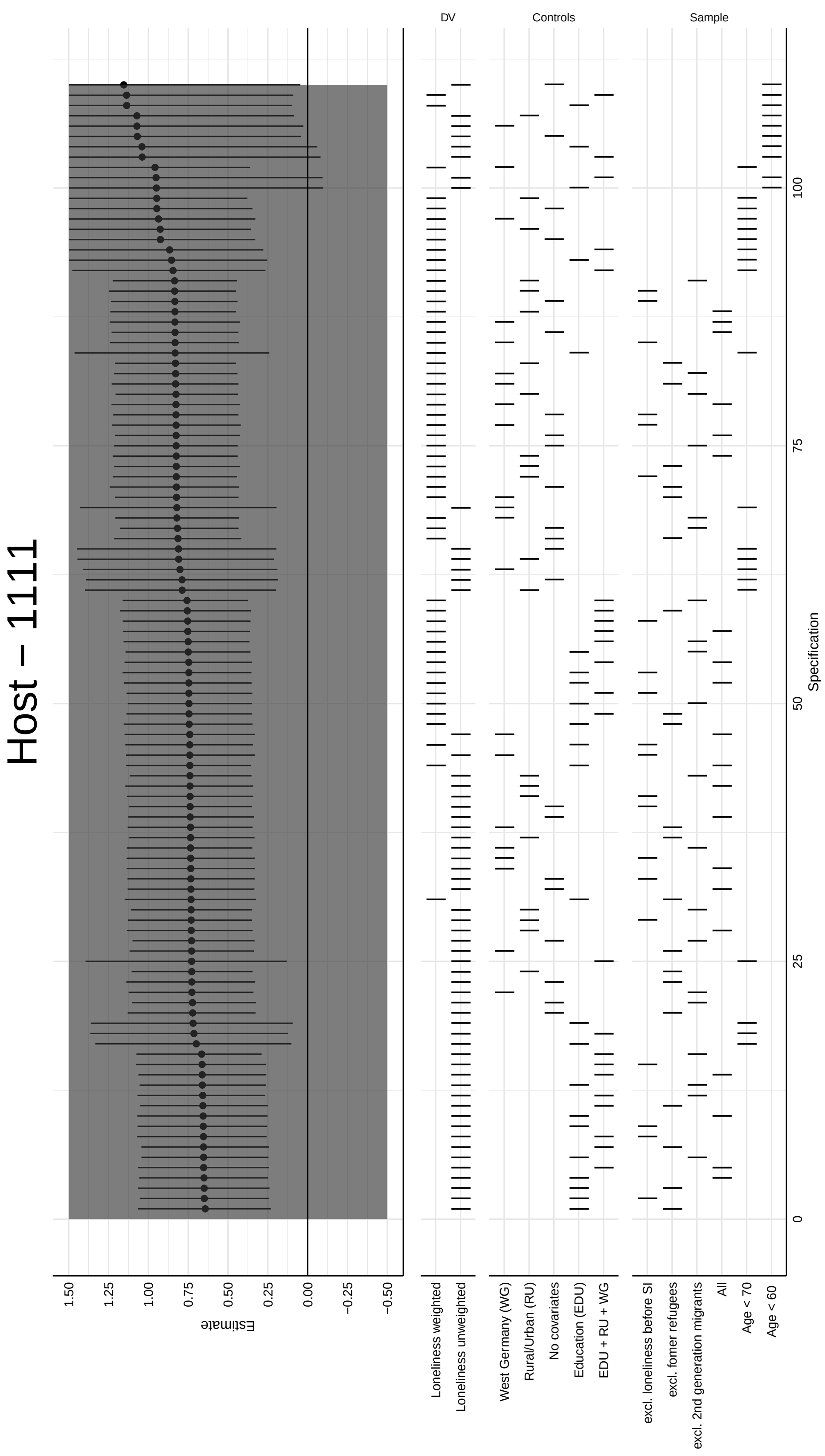

Host – 1111
Estimate
1.50
1.25
1.00
0.75
0.50
0.25
0.00
−0.25
−0.50
DV
Loneliness weighted
Loneliness unweighted
Controls
West Germany (WG)
Rural/Urban (RU)
No covariates
Education (EDU)
EDU + RU + WG
Sample
excl. loneliness before SI
excl. fomer refugees
excl. 2nd generation migrants
All
Age < 70
Age < 60
Specification
0
25
50
75
100

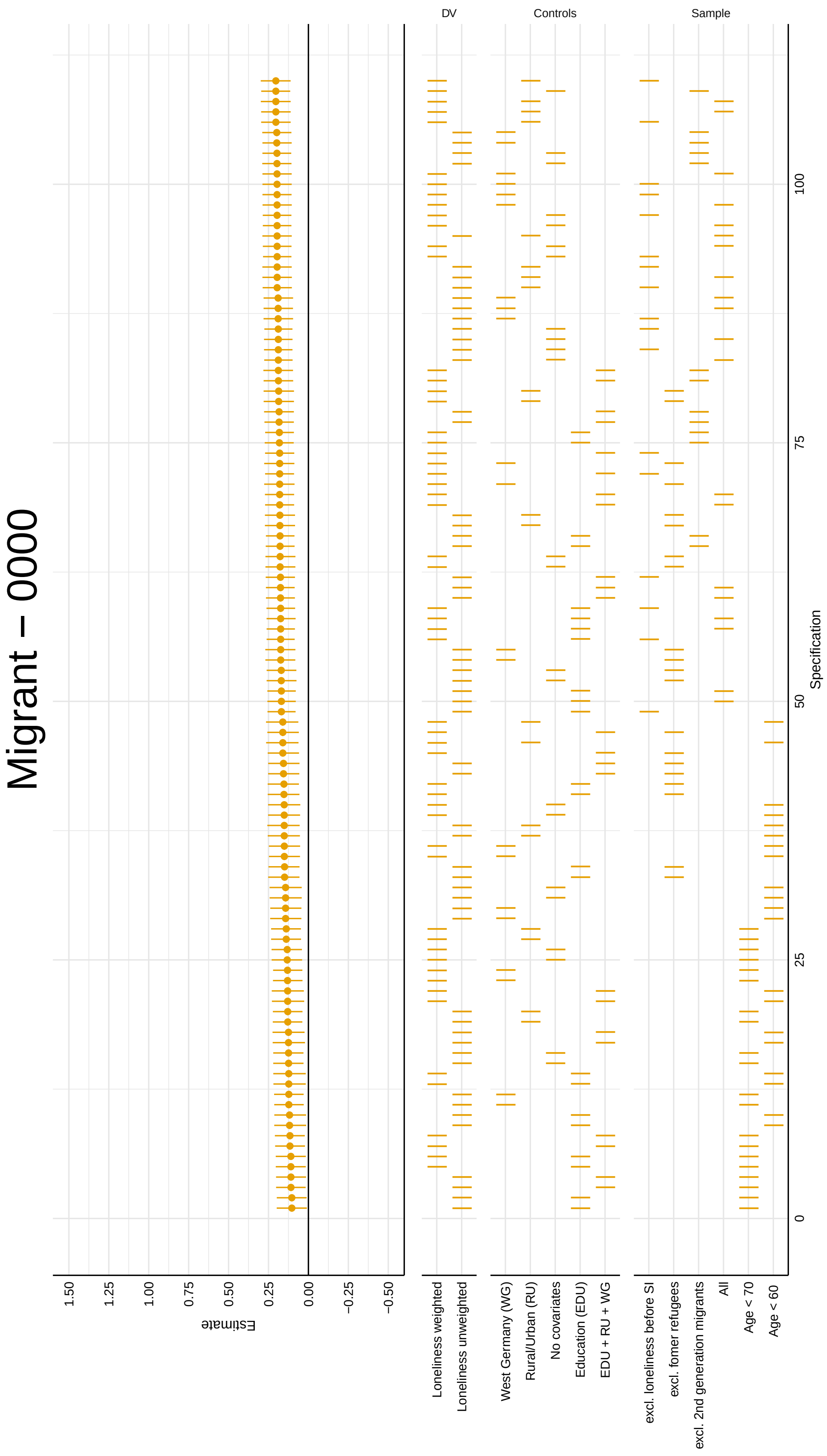
Migrant – 0000
Estimate
1.50
1.25
1.00
0.75
0.50
0.25
0.00
−0.25
−0.50
DV
Loneliness weighted
Loneliness unweighted
Controls
West Germany (WG)
Rural/Urban (RU)
No covariates
Education (EDU)
EDU + RU + WG
Sample
excl. loneliness before SI
excl. fomer refugees
excl. 2nd generation migrants
All
Age < 70
Age < 60
0
25
50
75
100
Specification

Migrant – 0001

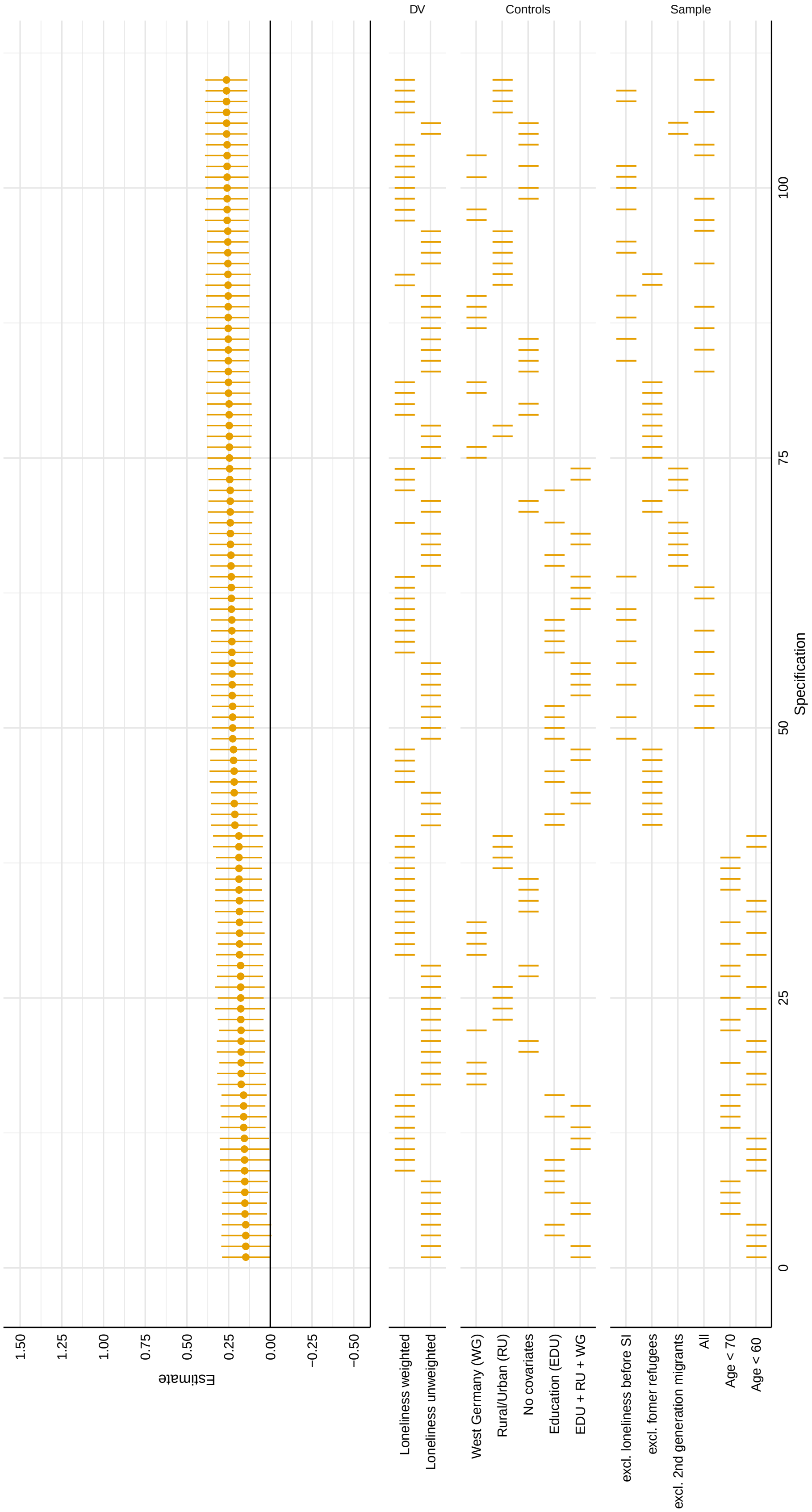

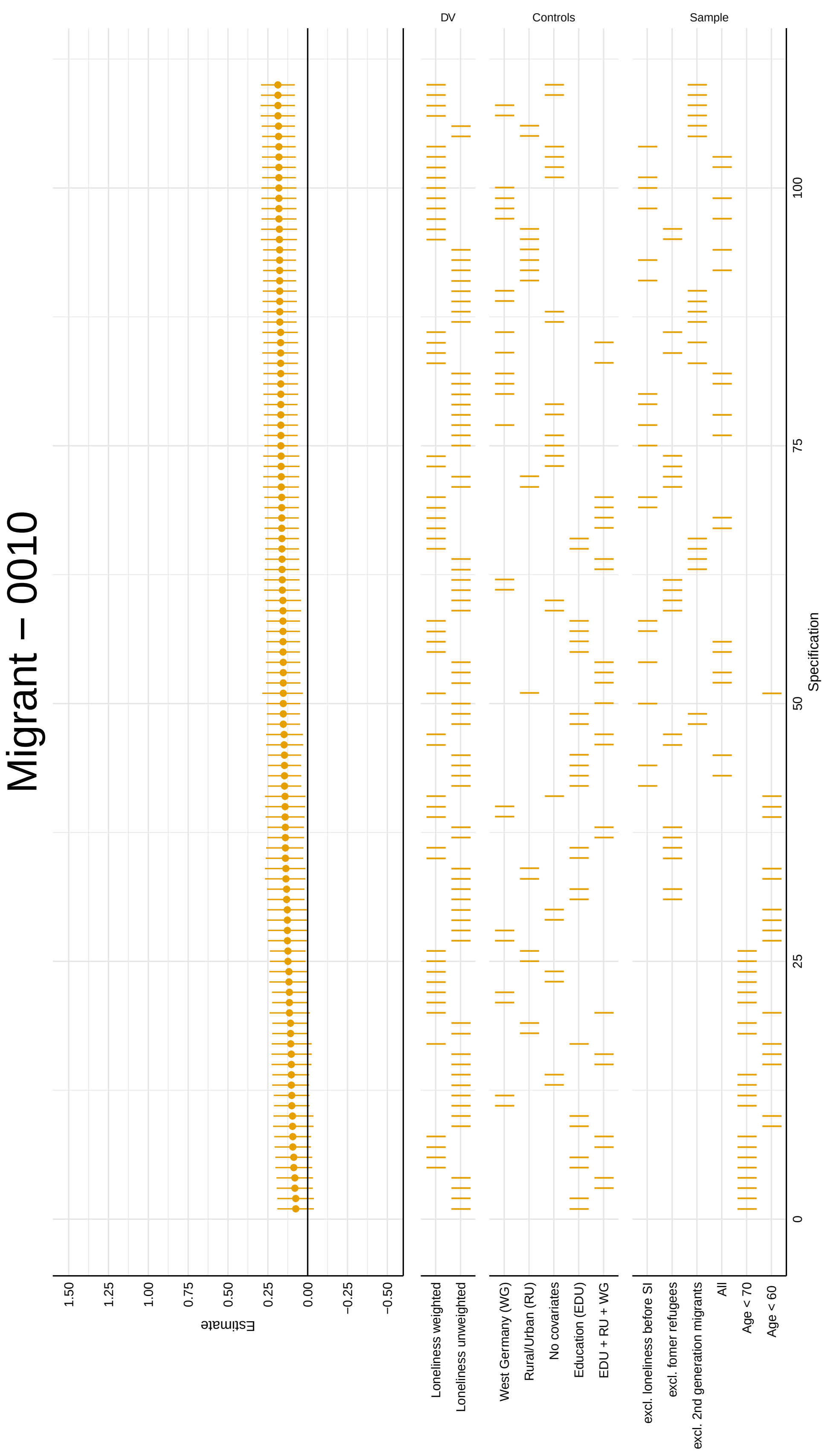

Migrant – 0010
Estimate
1.50
1.25
1.00
0.75
0.50
0.25
0.00
−0.25
−0.50
DV
Loneliness weighted
Loneliness unweighted
Controls
West Germany (WG)
Rural/Urban (RU)
No covariates
Education (EDU)
EDU + RU + WG
Sample
excl. loneliness before SI
excl. fomer refugees
excl. 2nd generation migrants
All
Age < 70
Age < 60
0
25
50
75
100
Specification

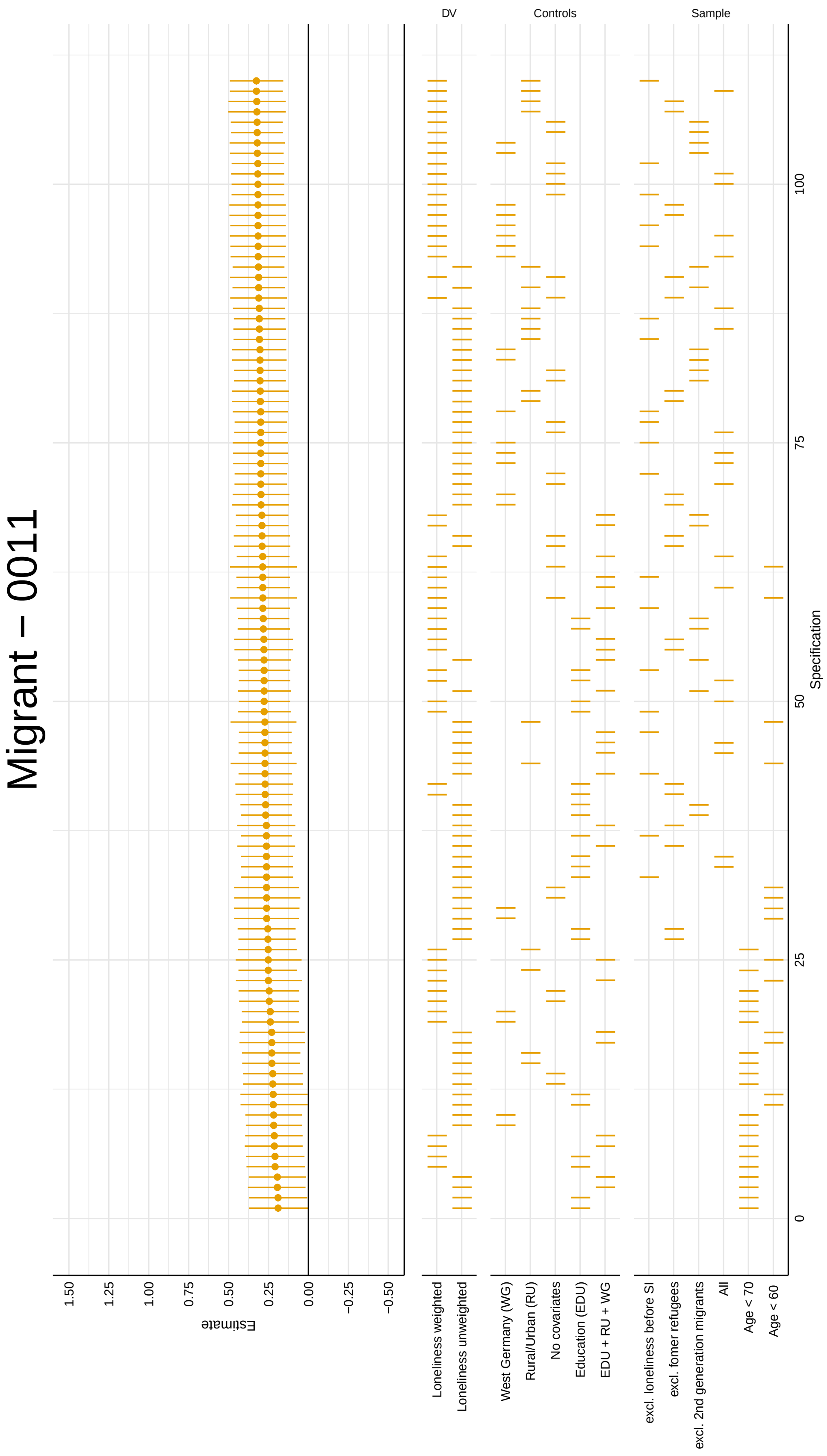
Migrant – 0011
Estimate
1.50
1.25
1.00
0.75
0.50
0.25
0.00
−0.25
−0.50
DV
Loneliness weighted
Loneliness unweighted
Controls
West Germany (WG)
Rural/Urban (RU)
No covariates
Education (EDU)
EDU + RU + WG
Sample
excl. loneliness before SI
excl. fomer refugees
excl. 2nd generation migrants
All
Age < 70
Age < 60
0
25
50
75
100
Specification

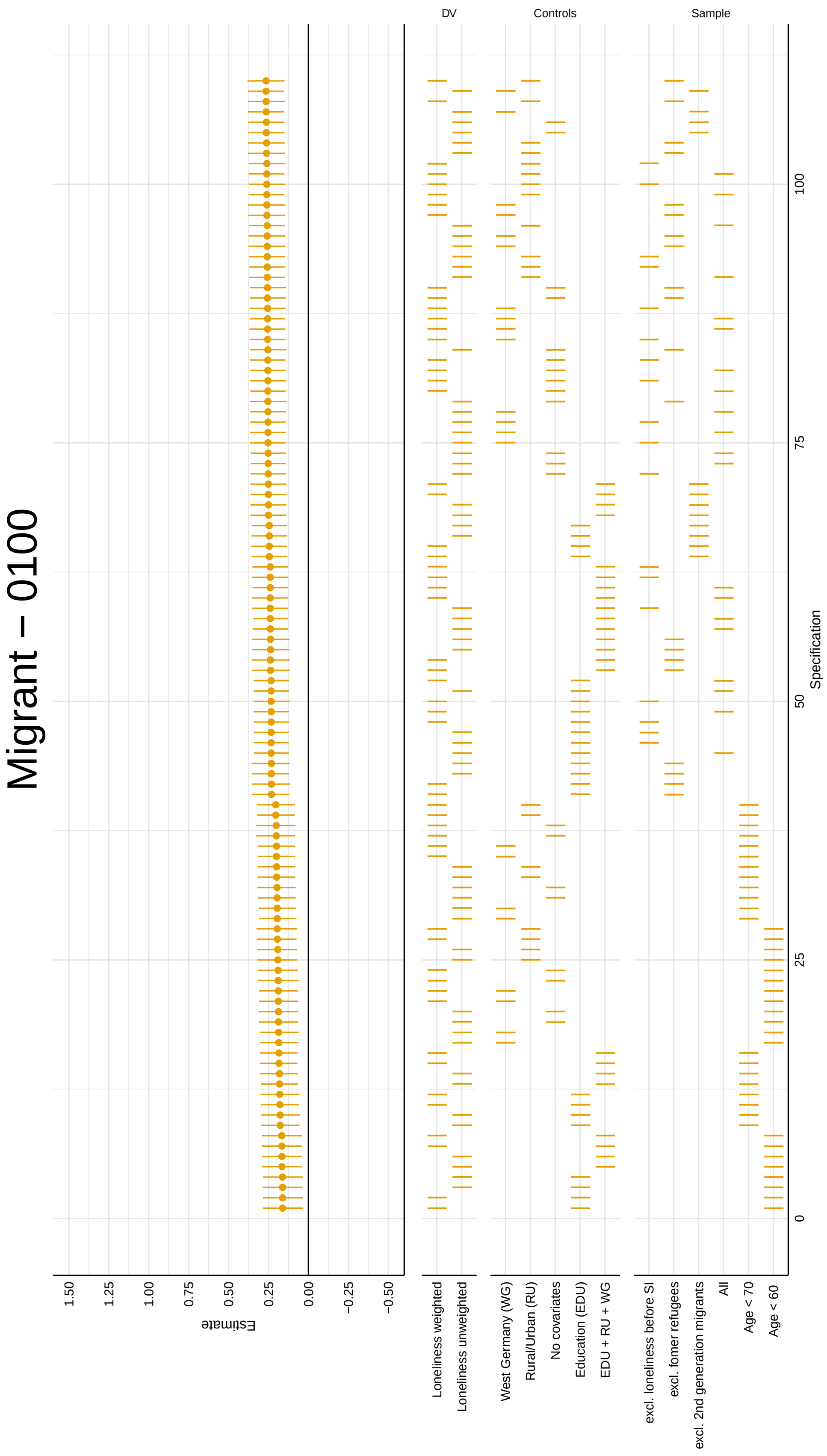

Migrant – 0100
Estimate
1.50
1.25
1.00
0.75
0.50
0.25
0.00
−0.25
−0.50
DV
Loneliness weighted
Loneliness unweighted
Controls
West Germany (WG)
Rural/Urban (RU)
No covariates
Education (EDU)
EDU + RU + WG
Sample
excl. loneliness before SI
excl. fomer refugees
excl. 2nd generation migrants
All
Age < 70
Age < 60
0
25
50
75
100
Specification

Migrant – 0101

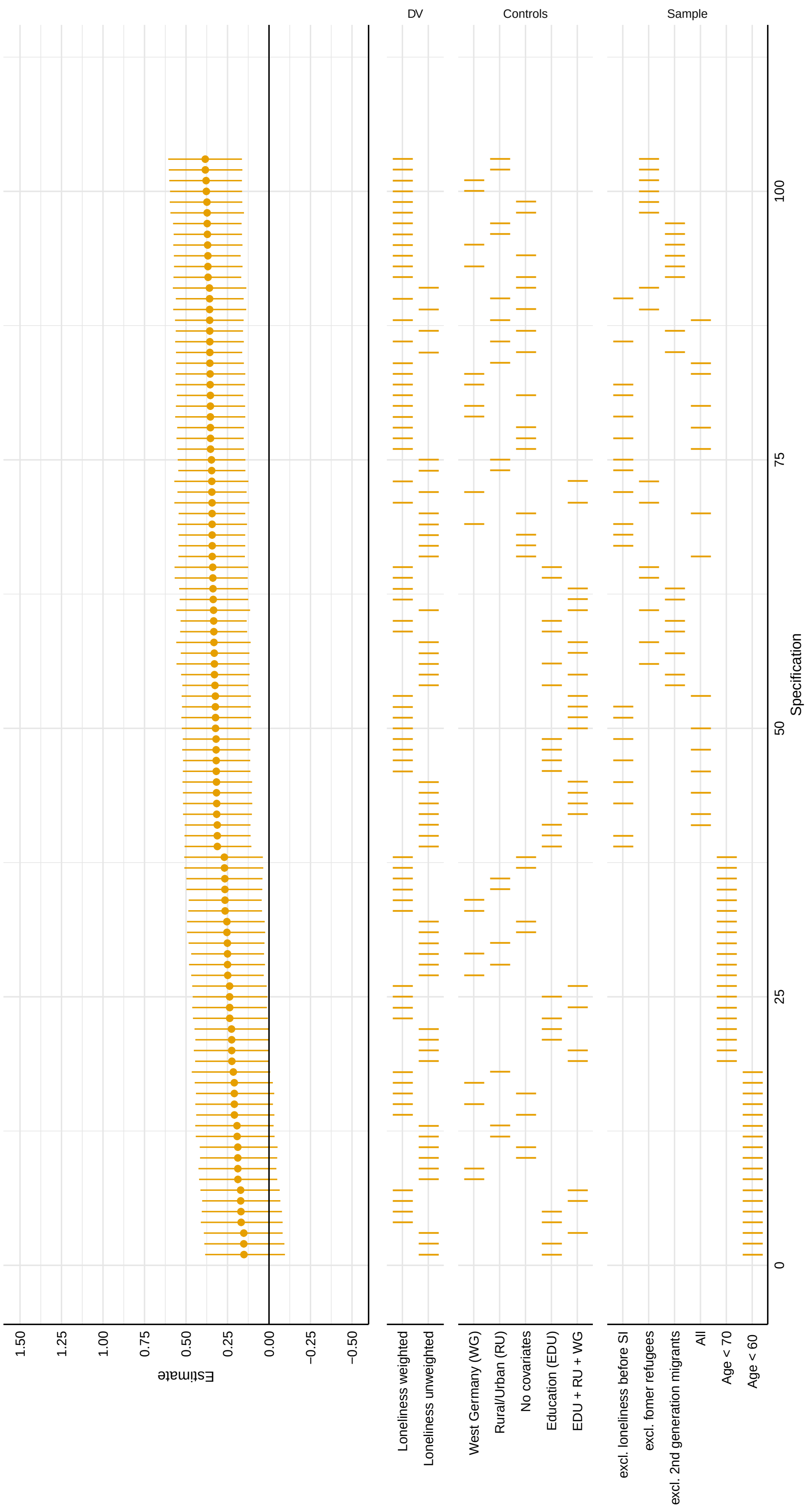

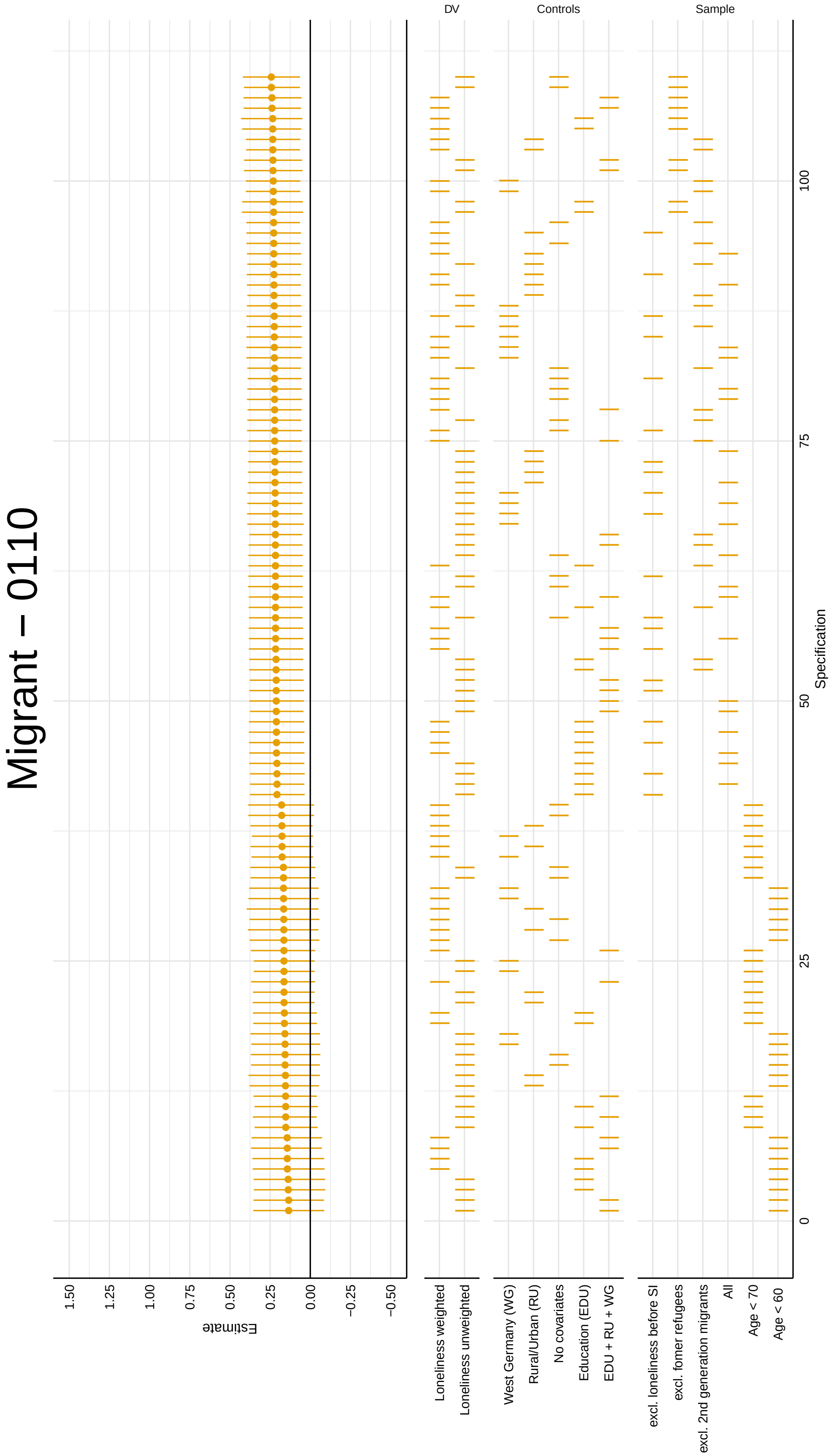
Migrant – 0110
Estimate
1.50
1.25
1.00
0.75
0.50
0.25
0.00
−0.25
−0.50
DV
Loneliness weighted
Loneliness unweighted
Controls
West Germany (WG)
Rural/Urban (RU)
No covariates
Education (EDU)
EDU + RU + WG
Sample
excl. loneliness before SI
excl. fomer refugees
excl. 2nd generation migrants
All
Age < 70
Age < 60
Specification
0
25
50
75
100

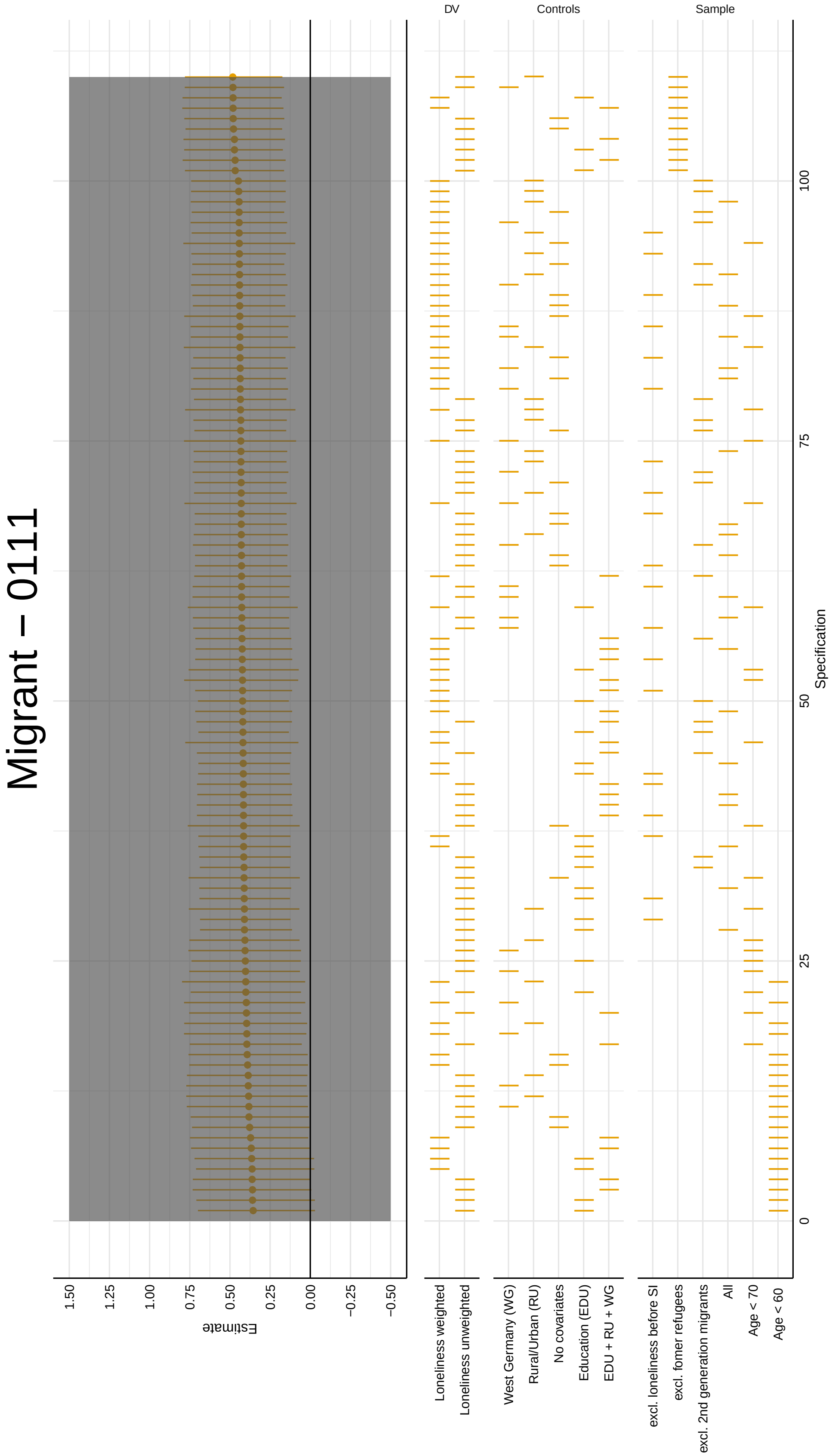
Migrant – 0111
Estimate
1.50
1.25
1.00
0.75
0.50
0.25
0.00
−0.25
−0.50
DV
Loneliness weighted
Loneliness unweighted
Controls
West Germany (WG)
Rural/Urban (RU)
No covariates
Education (EDU)
EDU + RU + WG
Sample
excl. loneliness before SI
excl. fomer refugees
excl. 2nd generation migrants
All
Age < 70
Age < 60
0
25
50
75
100
Specification

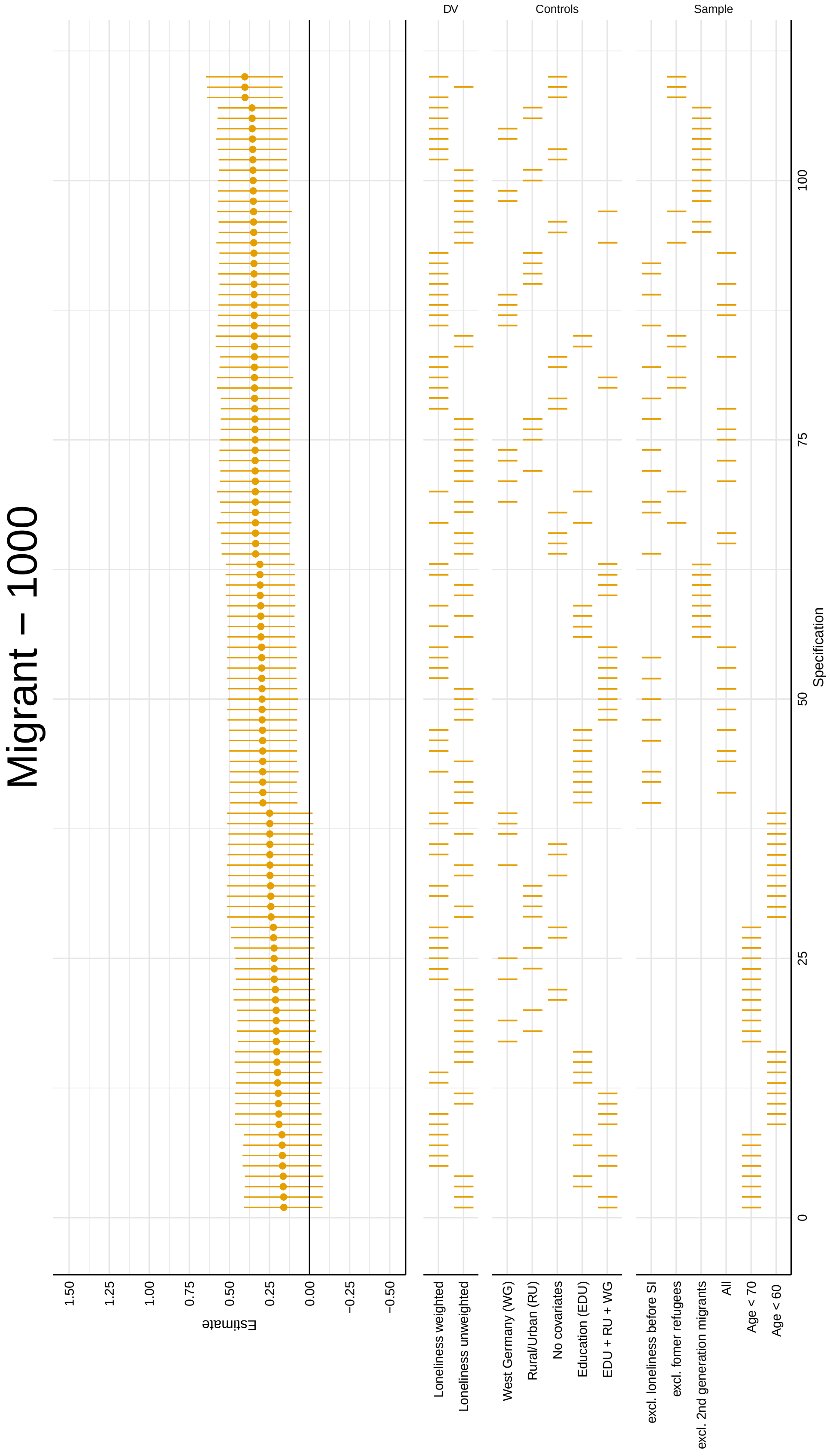
Migrant – 1000
Estimate
1.50
1.25
1.00
0.75
0.50
0.25
0.00
−0.25
−0.50
DV
Loneliness weighted
Loneliness unweighted
Controls
West Germany (WG)
Rural/Urban (RU)
No covariates
Education (EDU)
EDU + RU + WG
Sample
excl. loneliness before SI
excl. fomer refugees
excl. 2nd generation migrants
All
Age < 70
Age < 60
0
25
50
75
100
Specification

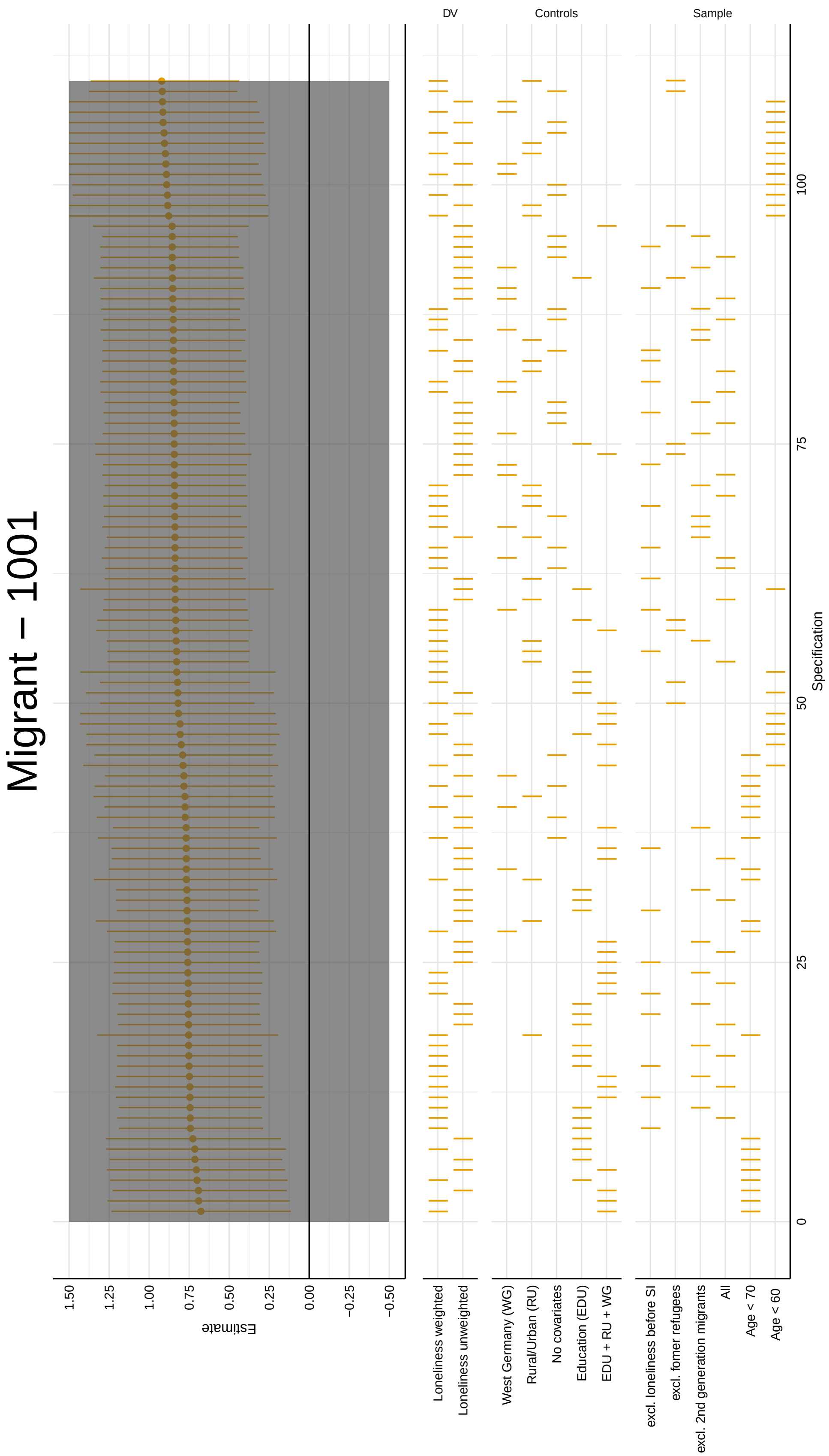

Migrant – 1001
Estimate
1.50
1.25
1.00
0.75
0.50
0.25
0.00
−0.25
−0.50
DV
Loneliness weighted
Loneliness unweighted
Controls
West Germany (WG)
Rural/Urban (RU)
No covariates
Education (EDU)
EDU + RU + WG
Sample
excl. loneliness before SI
excl. fomer refugees
excl. 2nd generation migrants
All
Age < 70
Age < 60
0
25
50
75
100
Specification

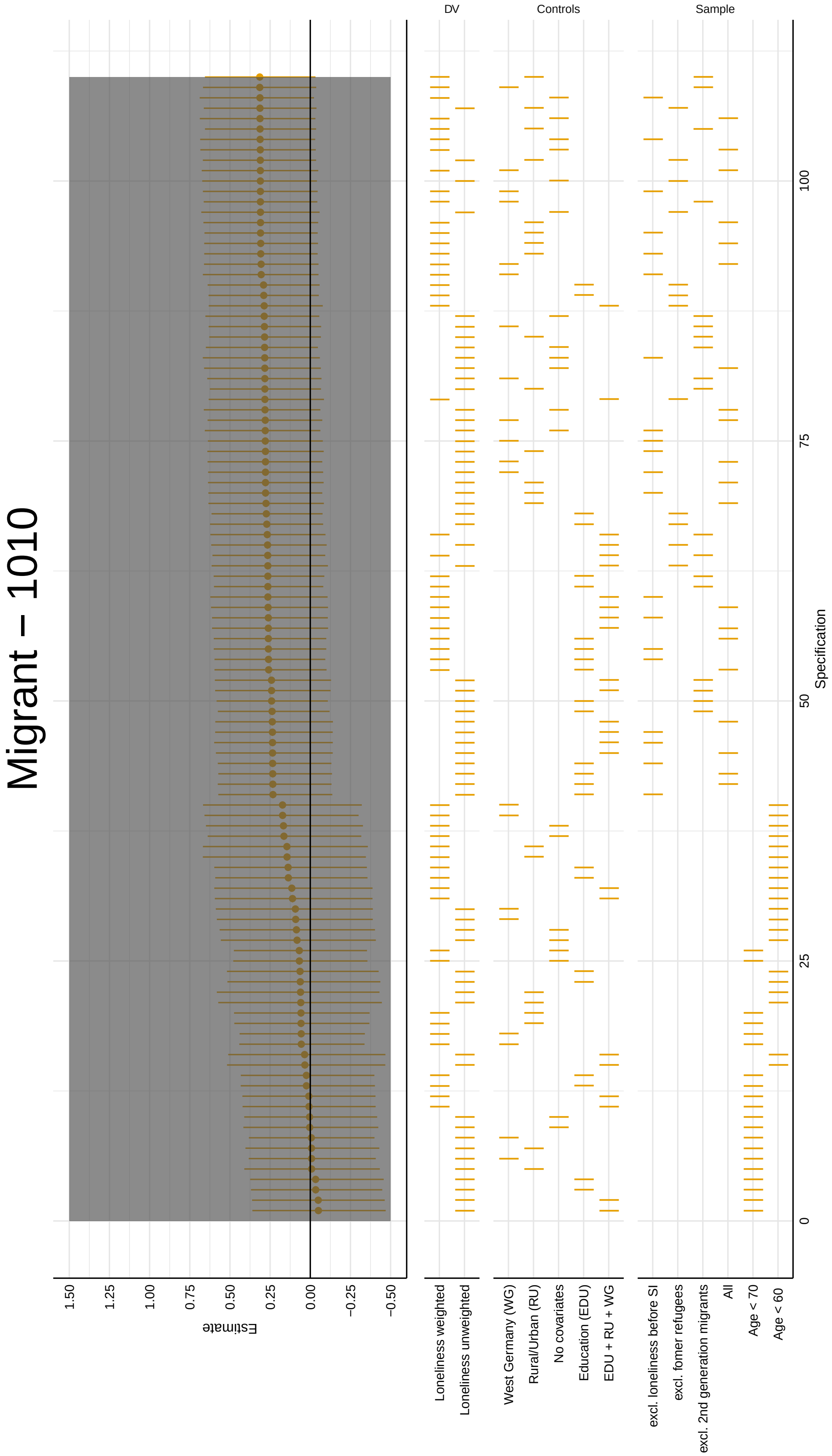

Migrant – 1010
Estimate
1.50
1.25
1.00
0.75
0.50
0.25
0.00
−0.25
−0.50
DV
Loneliness weighted
Loneliness unweighted
Controls
West Germany (WG)
Rural/Urban (RU)
No covariates
Education (EDU)
EDU + RU + WG
Sample
excl. loneliness before SI
excl. fomer refugees
excl. 2nd generation migrants
All
Age < 70
Age < 60
Specification
0
25
50
75
100

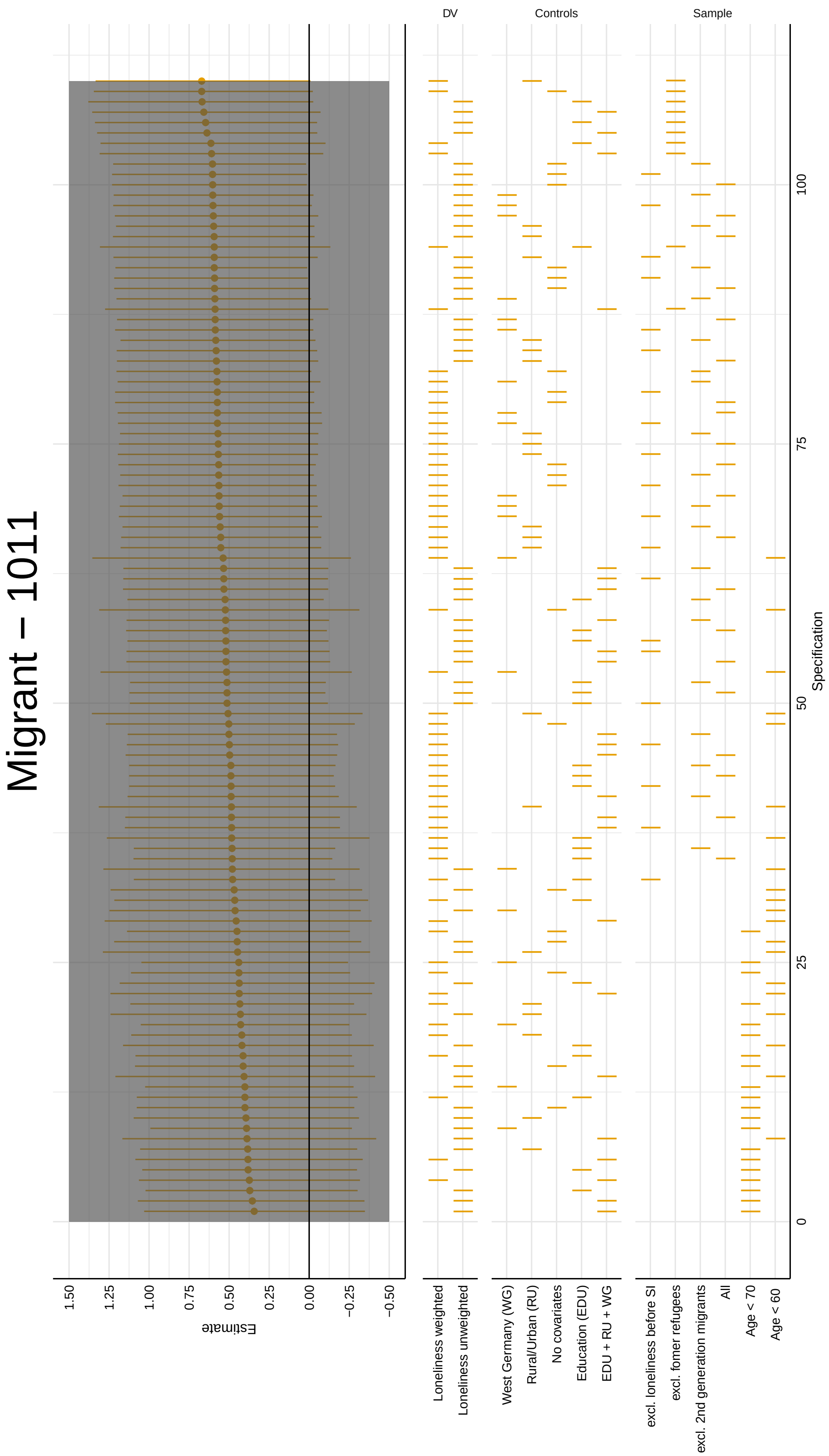

Migrant – 1011
Estimate
1.50
1.25
1.00
0.75
0.50
0.25
0.00
−0.25
−0.50
DV
Loneliness weighted
Loneliness unweighted
Controls
West Germany (WG)
Rural/Urban (RU)
No covariates
Education (EDU)
EDU + RU + WG
Sample
excl. loneliness before SI
excl. fomer refugees
excl. 2nd generation migrants
All
Age < 70
Age < 60
Specification
0
25
50
75
100

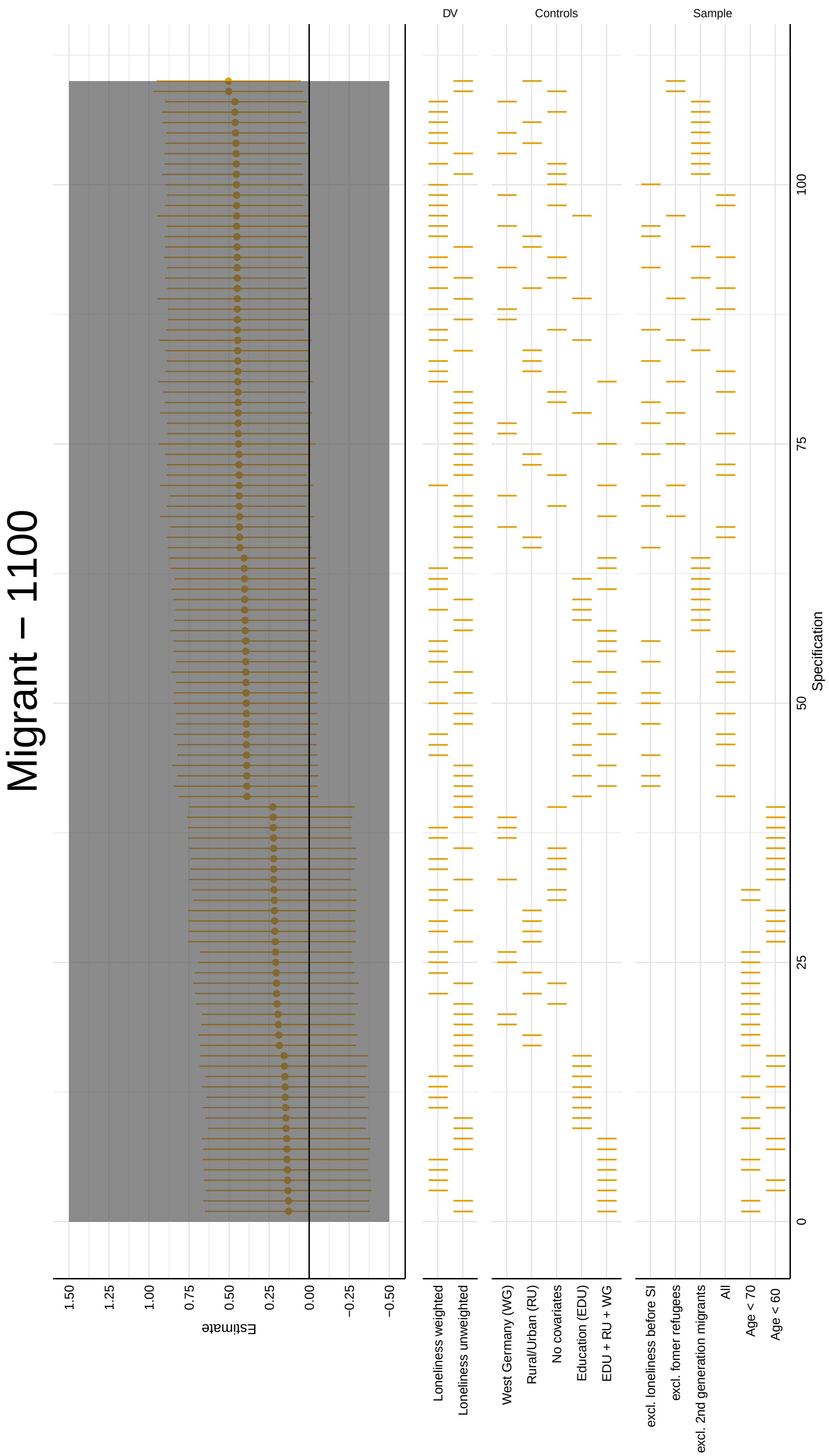
Migrant – 1100
Estimate
1.50
1.25
1.00
0.75
0.50
0.25
0.00
−0.25
−0.50
DV
Loneliness weighted
Loneliness unweighted
Controls
West Germany (WG)
Rural/Urban (RU)
No covariates
Education (EDU)
EDU + RU + WG
Sample
excl. loneliness before SI
excl. fomer refugees
excl. 2nd generation migrants
All
Age < 70
Age < 60
0
25
50
75
100
Specification

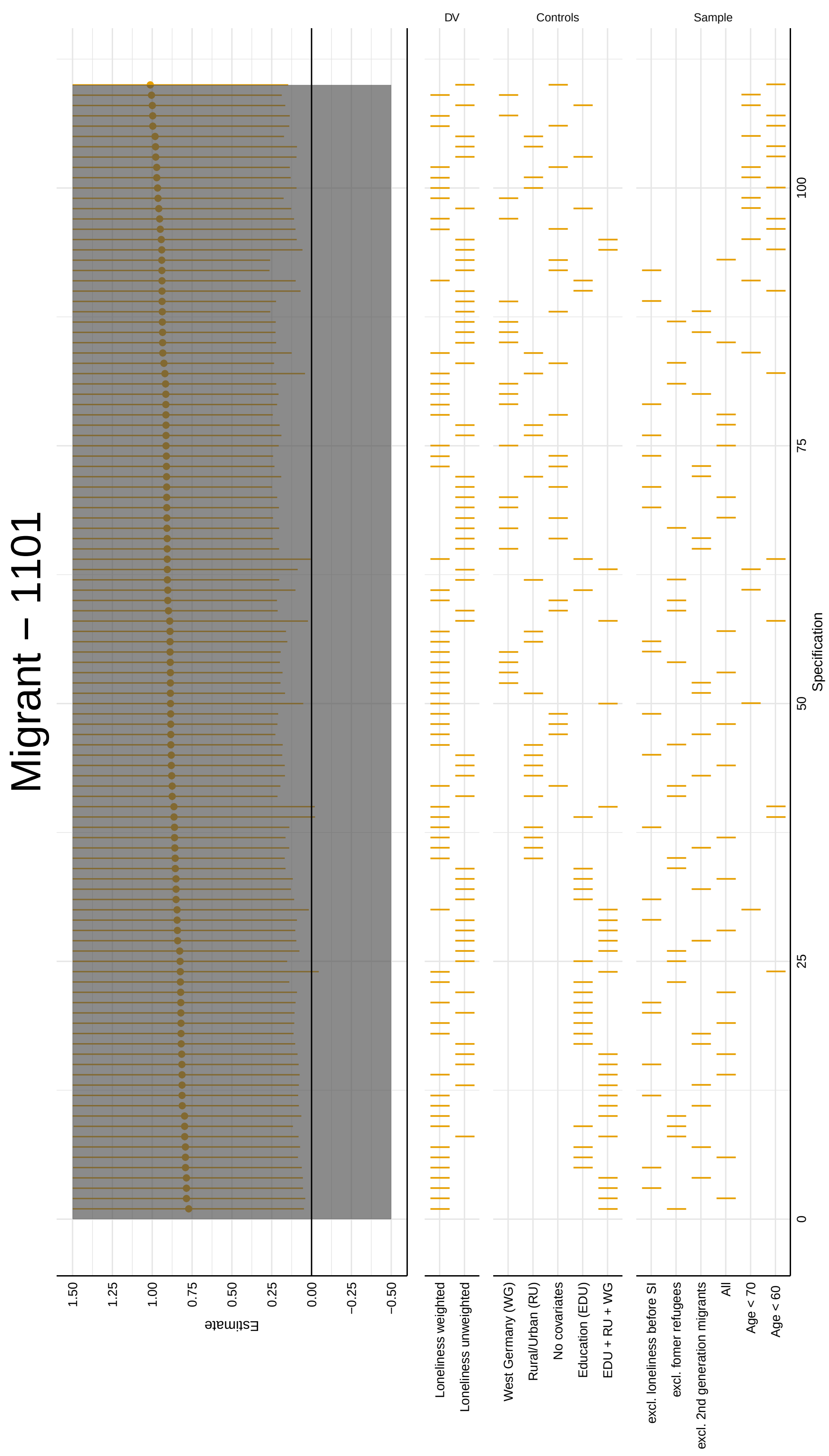
Migrant – 1101
Estimate
1.50
1.25
1.00
0.75
0.50
0.25
0.00
−0.25
−0.50
DV
Controls
Sample
Loneliness weighted
Loneliness unweighted
West Germany (WG)
Rural/Urban (RU)
No covariates
Education (EDU)
EDU + RU + WG
excl. loneliness before SI
excl. fomer refugees
excl. 2nd generation migrants
All
Age < 70
Age < 60
0
25
50
75
100
Specification

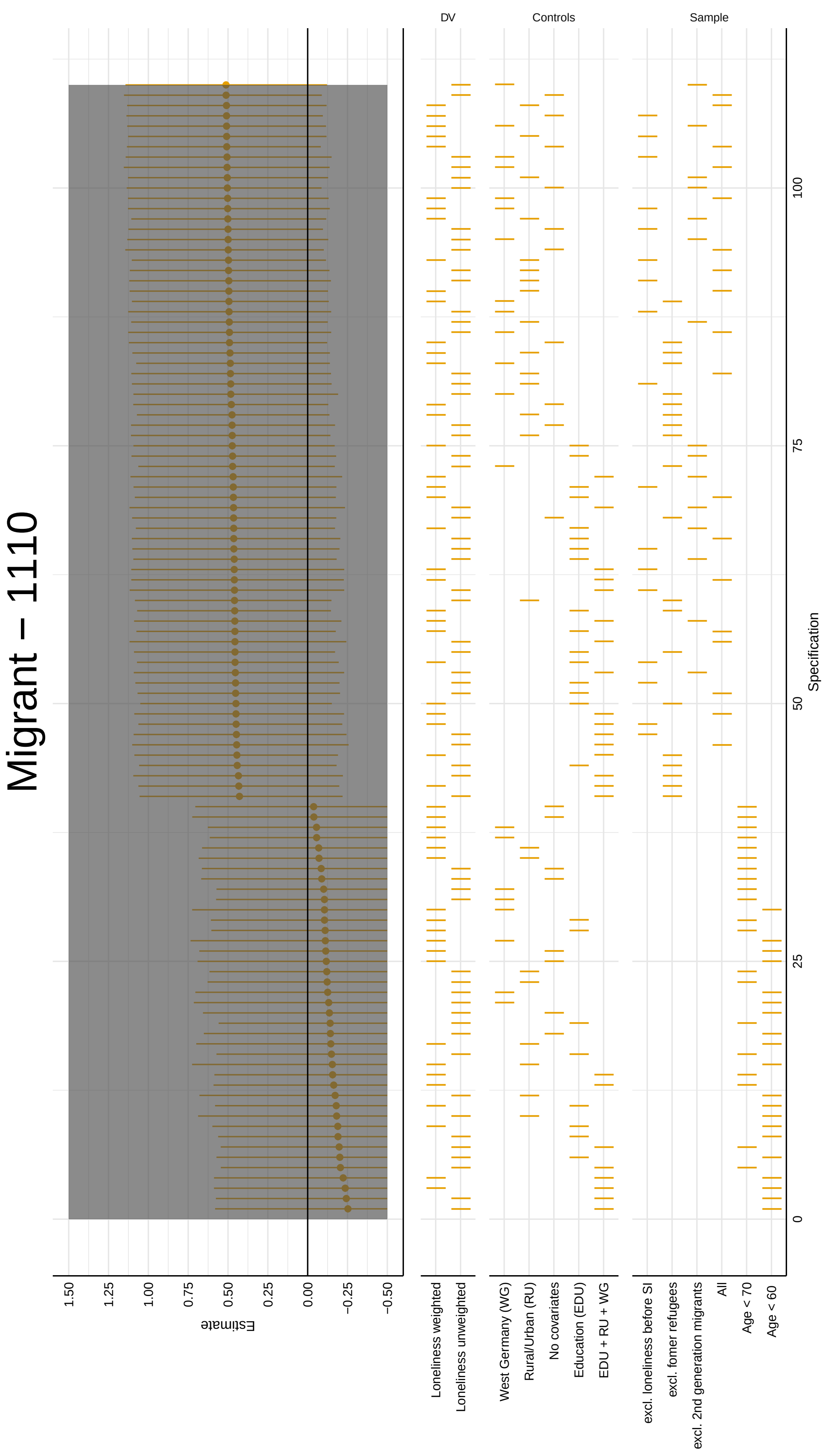

Migrant – 1110
Estimate
1.50
1.25
1.00
0.75
0.50
0.25
0.00
−0.25
−0.50
DV
Loneliness weighted
Loneliness unweighted
Controls
West Germany (WG)
Rural/Urban (RU)
No covariates
Education (EDU)
EDU + RU + WG
Sample
excl. loneliness before SI
excl. fomer refugees
excl. 2nd generation migrants
All
Age < 70
Age < 60
0
25
50
75
100
Specification

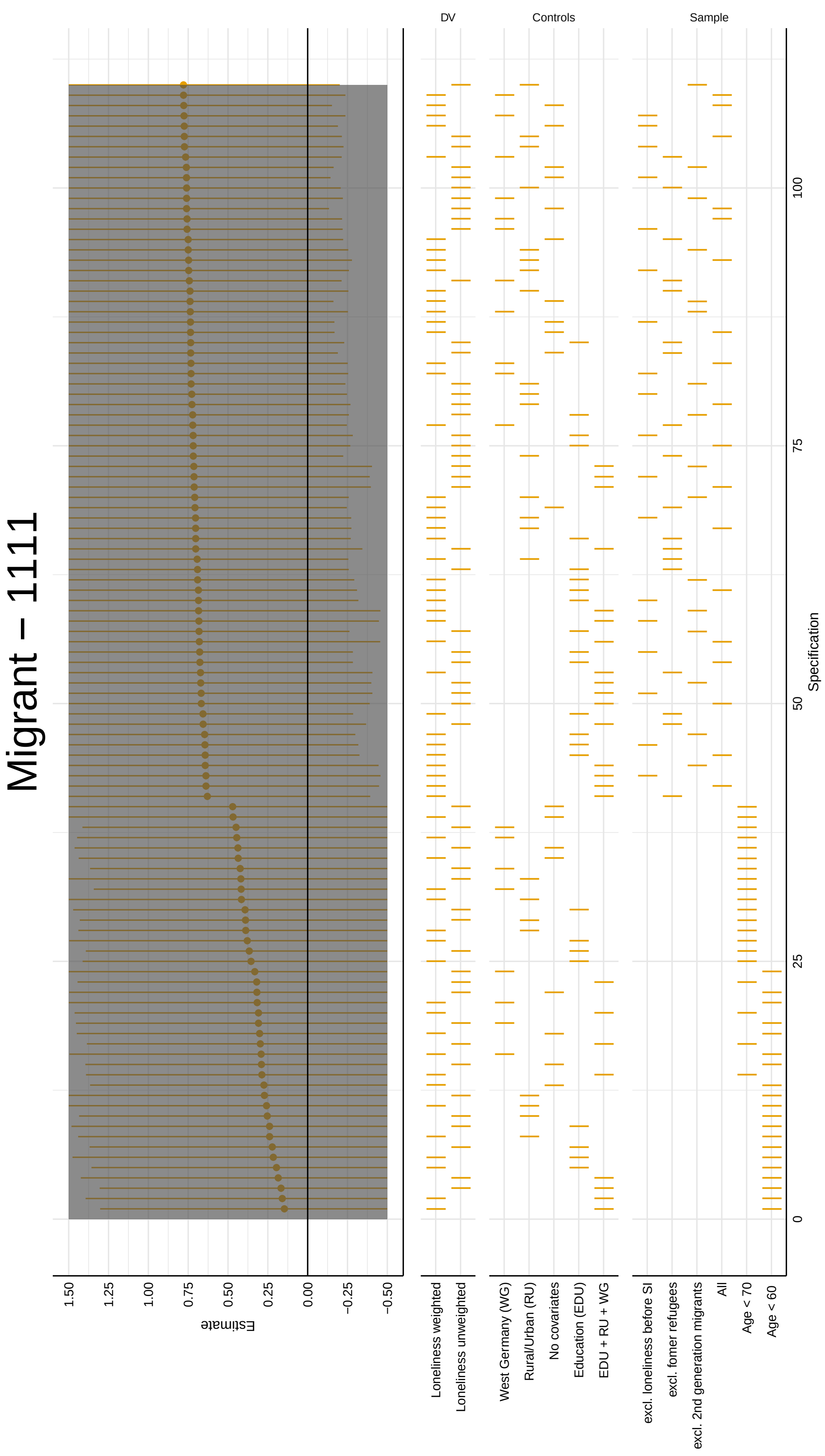
Migrant – 1111
Estimate
1.50
1.25
1.00
0.75
0.50
0.25
0.00
−0.25
−0.50
DV
Loneliness weighted
Loneliness unweighted
Controls
West Germany (WG)
Rural/Urban (RU)
No covariates
Education (EDU)
EDU + RU + WG
Sample
excl. loneliness before SI
excl. fomer refugees
excl. 2nd generation migrants
All
Age < 70
Age < 60
Specification
0
25
50
75
100

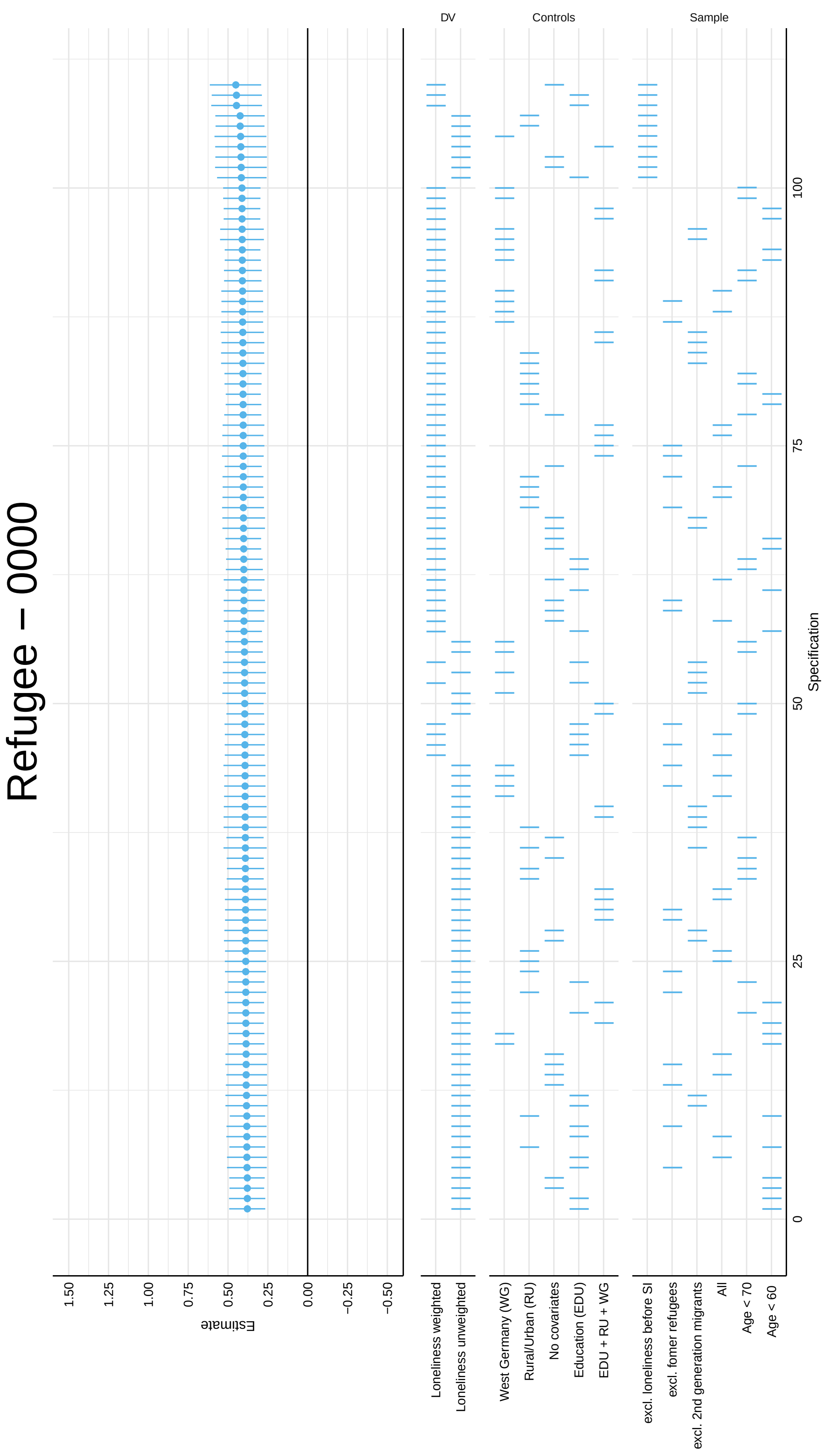

Refugee – 0000
Estimate
1.50
1.25
1.00
0.75
0.50
0.25
0.00
−0.25
−0.50
DV
Loneliness weighted
Loneliness unweighted
Controls
West Germany (WG)
Rural/Urban (RU)
No covariates
Education (EDU)
EDU + RU + WG
Sample
excl. loneliness before SI
excl. fomer refugees
excl. 2nd generation migrants
All
Age < 70
Age < 60
0
25
50
75
100
Specification

Refugee – 0001

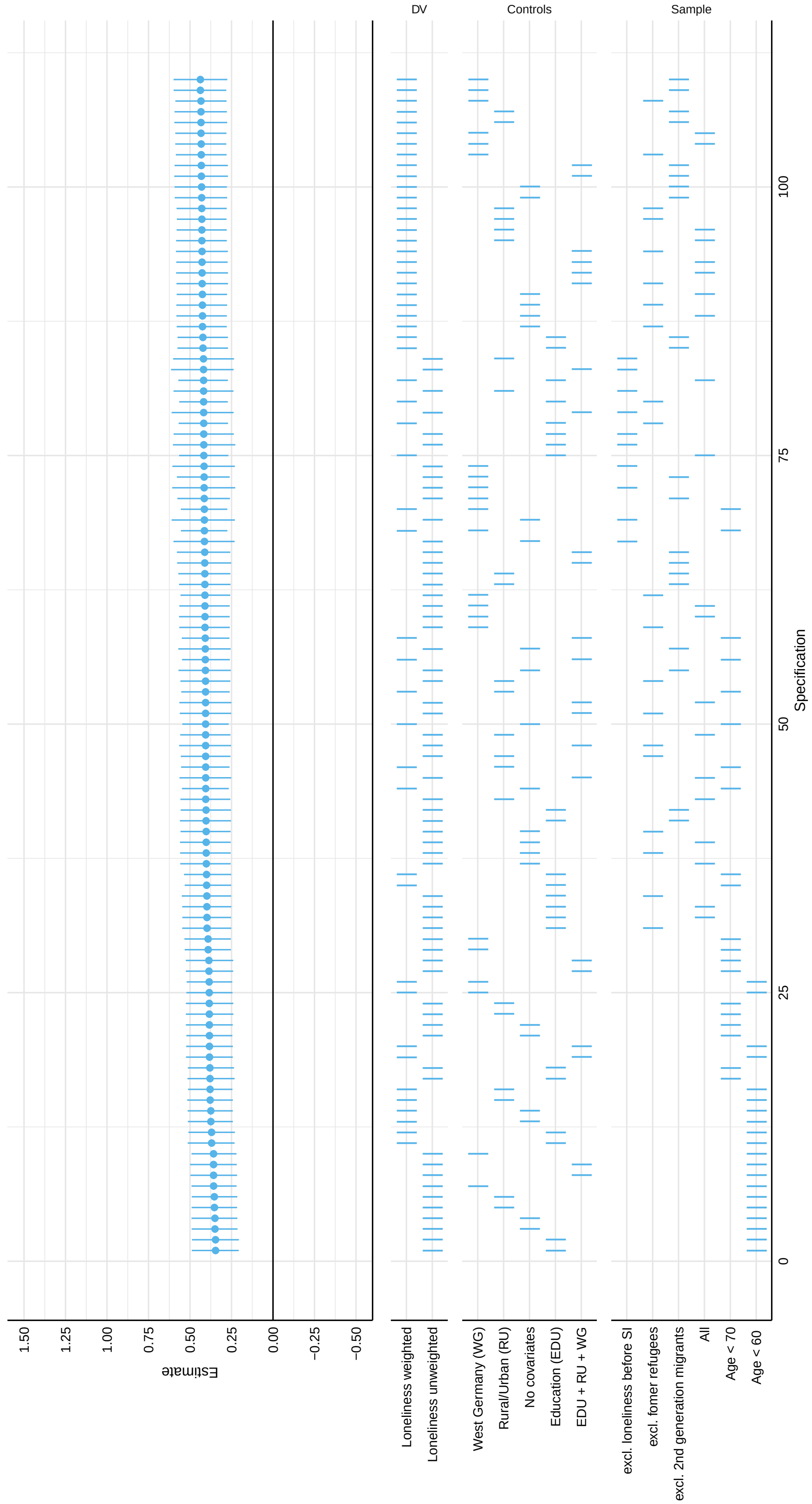

Refugee – 0010

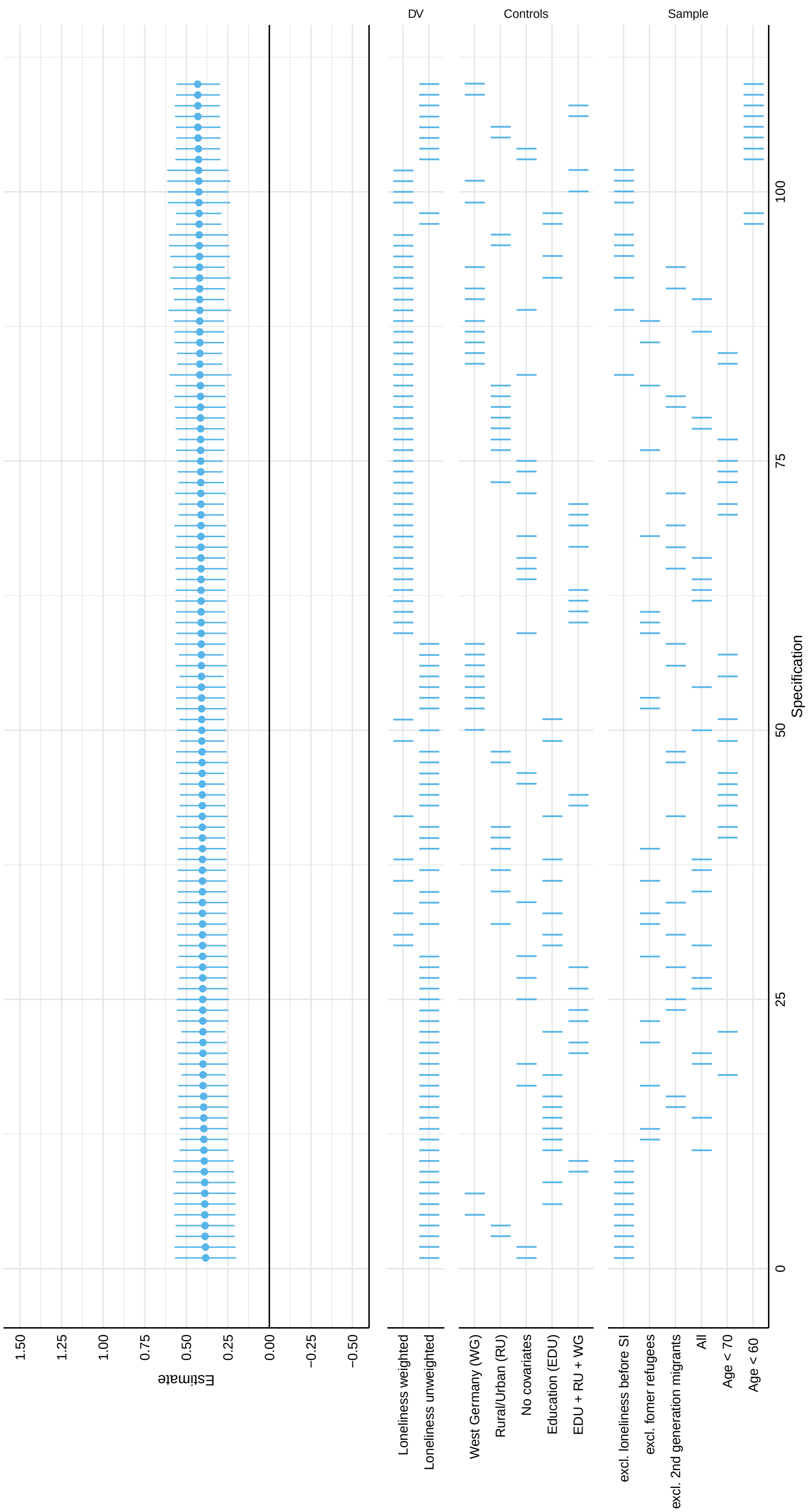

Refugee – 0011

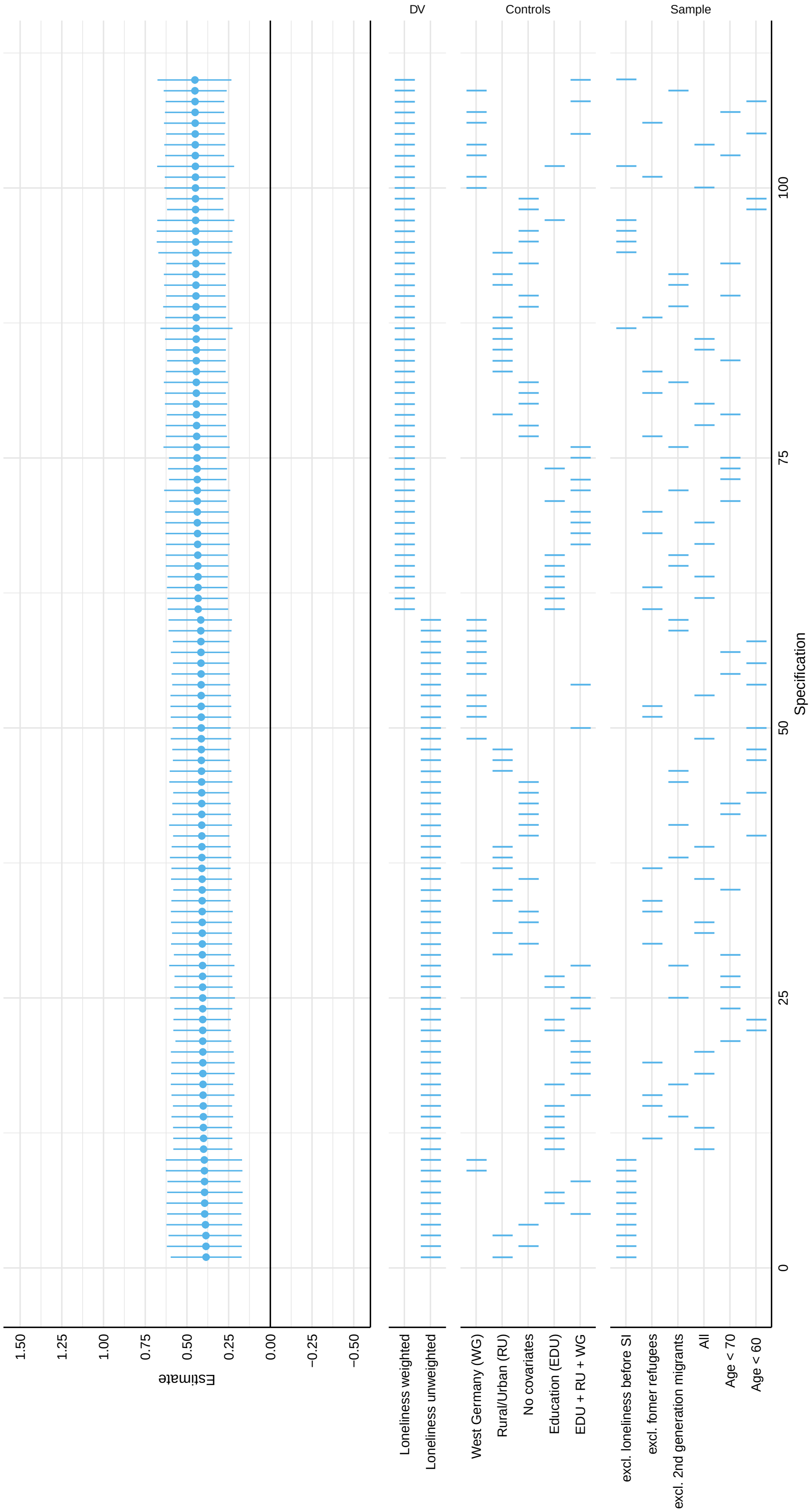

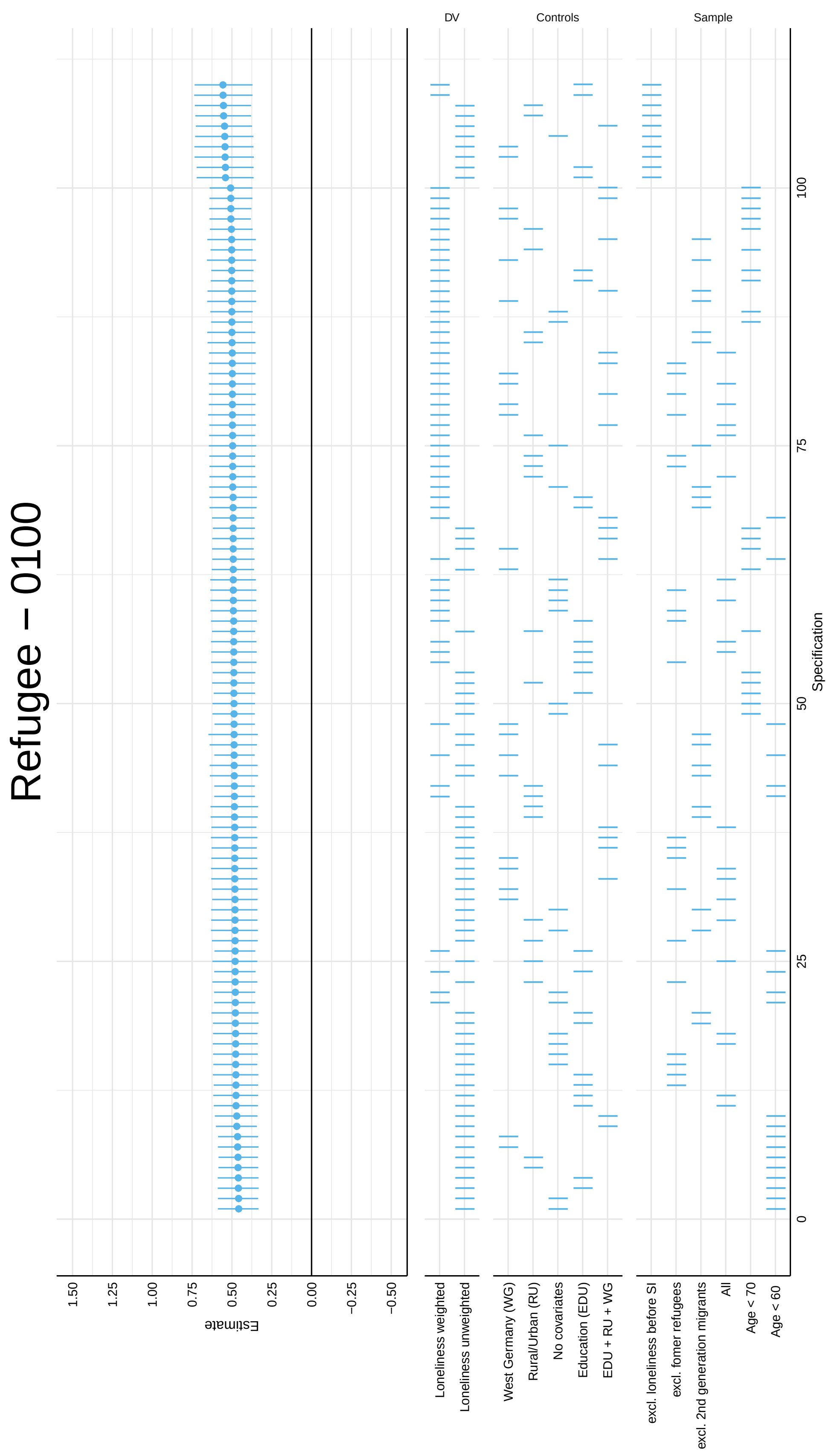

Refugee – 0100
Estimate
1.50
1.25
1.00
0.75
0.50
0.25
0.00
−0.25
−0.50
DV
Loneliness weighted
Loneliness unweighted
Controls
West Germany (WG)
Rural/Urban (RU)
No covariates
Education (EDU)
EDU + RU + WG
Sample
excl. loneliness before SI
excl. fomer refugees
excl. 2nd generation migrants
All
Age < 70
Age < 60
0
25
50
75
100
Specification

Refugee − 0101

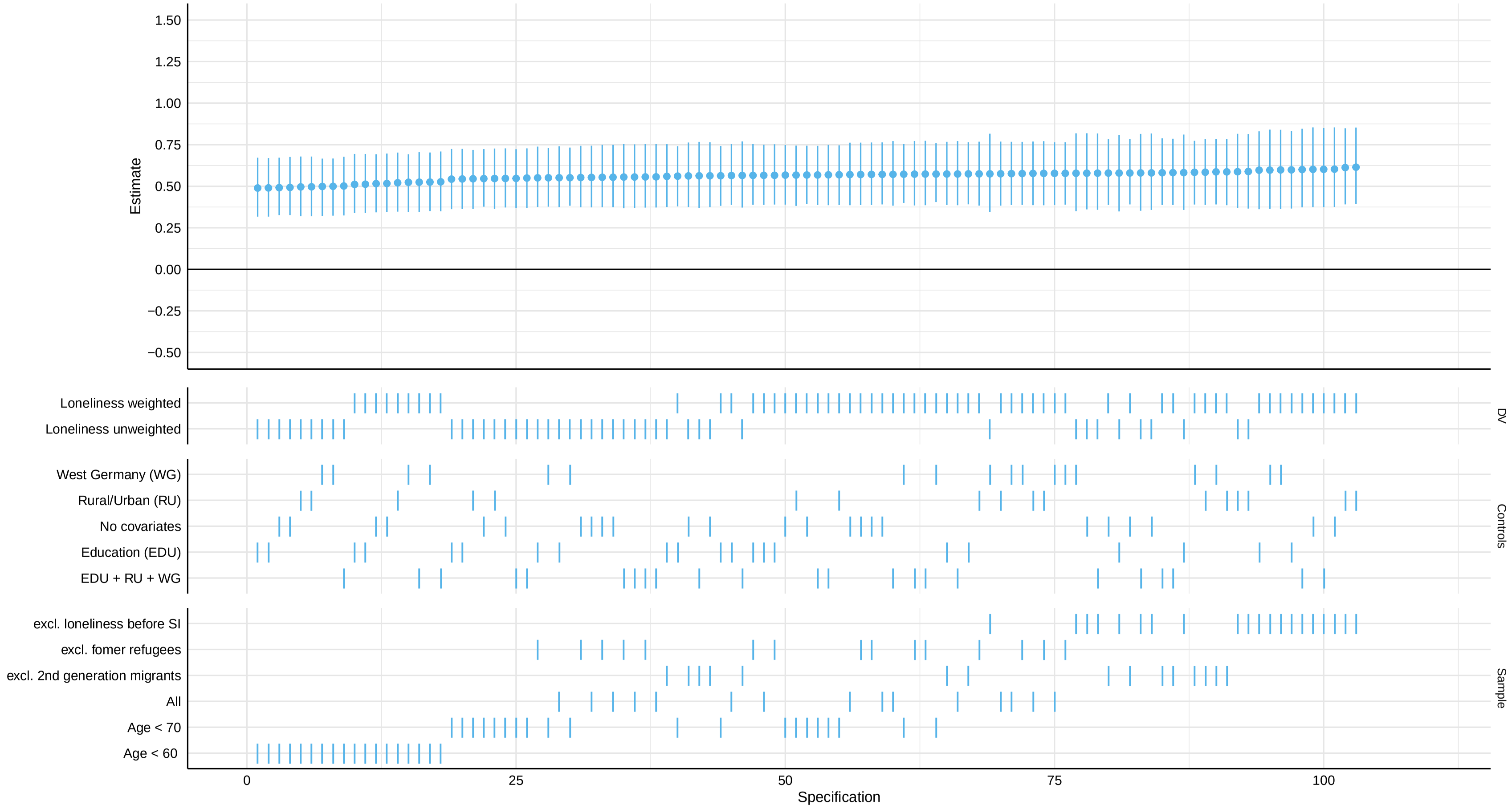

Refugee – 0110

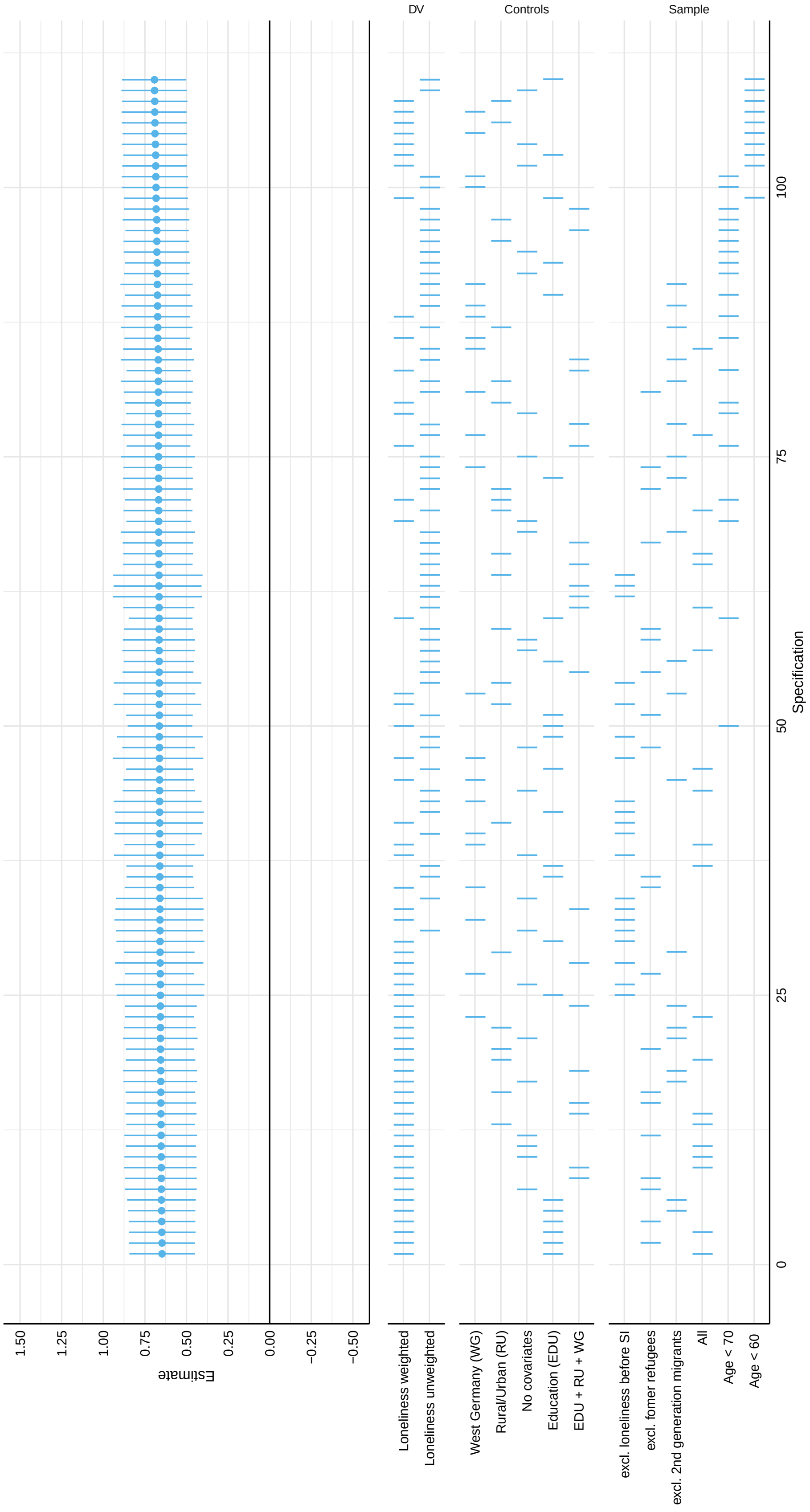

Refugee – 0111

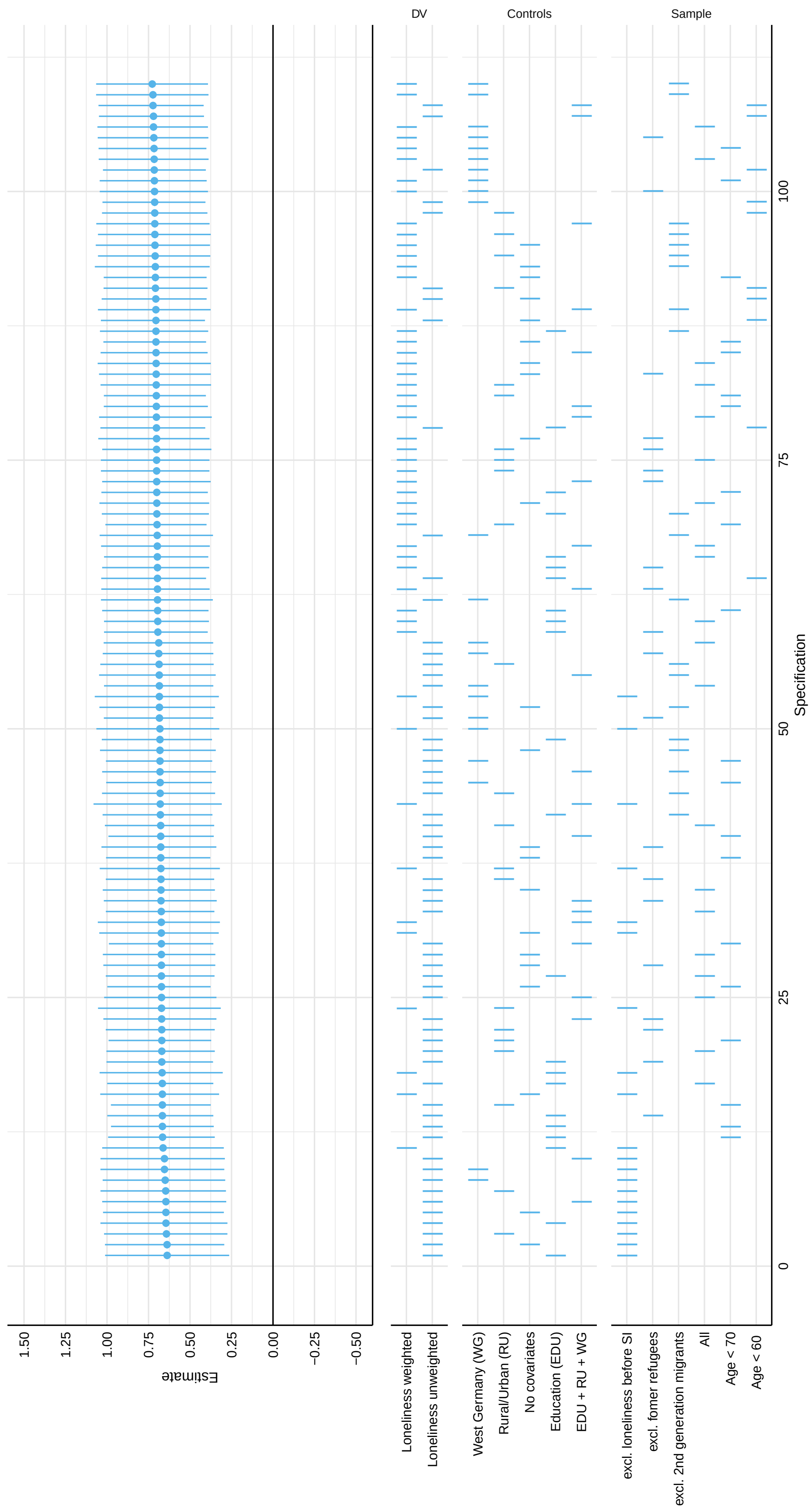

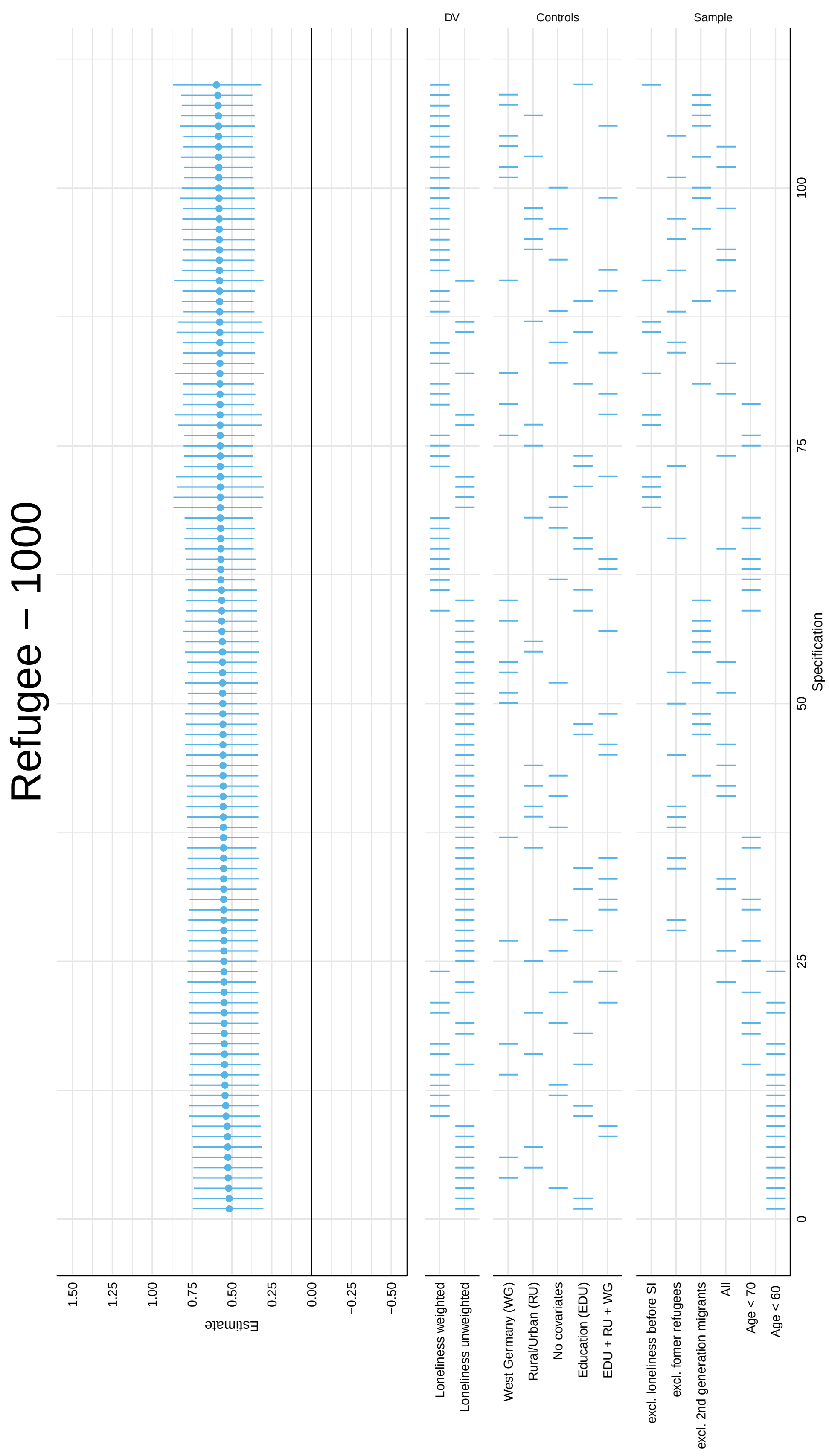
Refugee – 1000
Estimate
1.50
1.25
1.00
0.75
0.50
0.25
0.00
−0.25
−0.50
DV
Loneliness weighted
Loneliness unweighted
Controls
West Germany (WG)
Rural/Urban (RU)
No covariates
Education (EDU)
EDU + RU + WG
Sample
excl. loneliness before SI
excl. fomer refugees
excl. 2nd generation migrants
All
Age < 70
Age < 60
0
25
50
75
100
Specification

Refugee − 1001

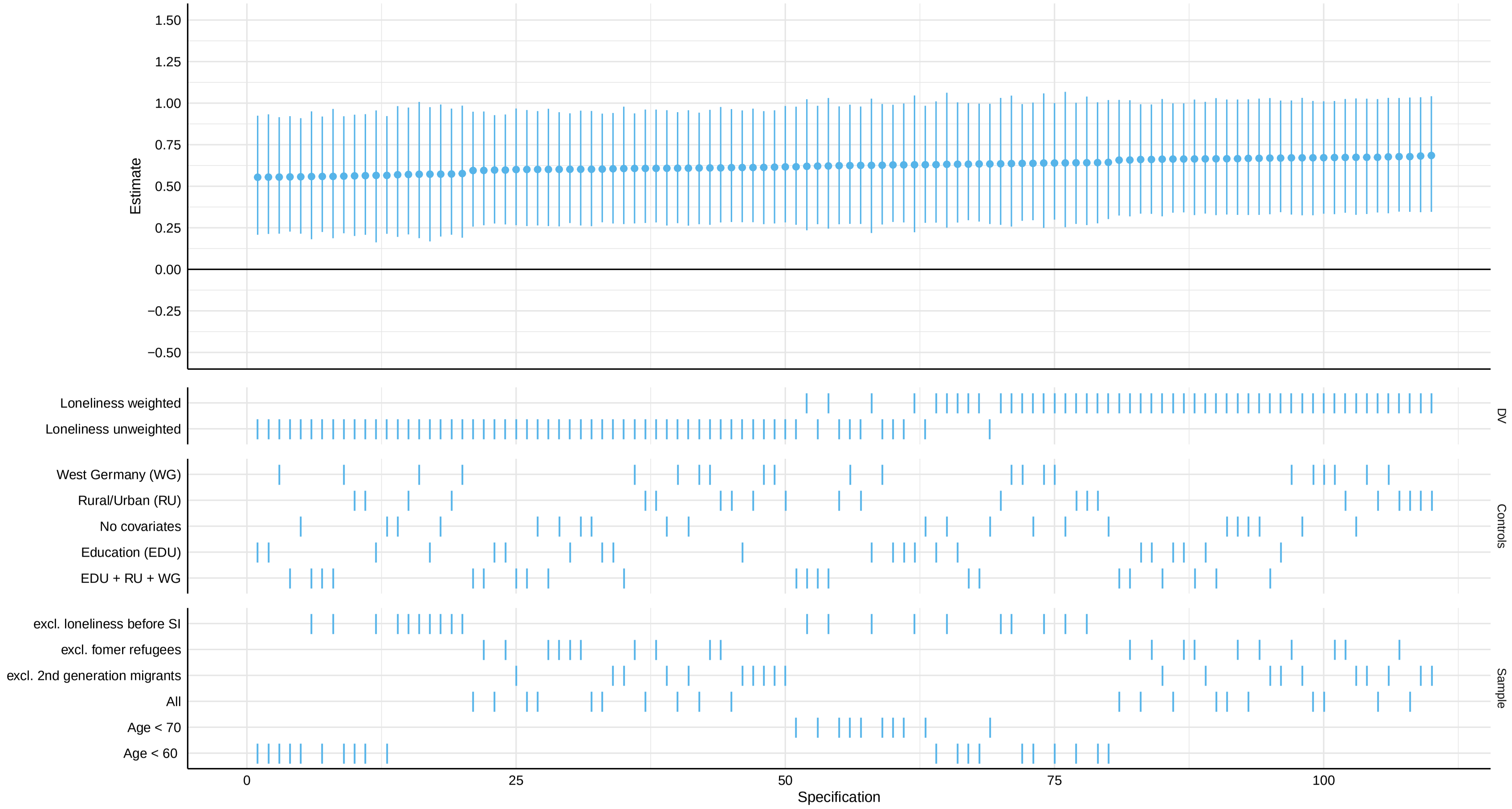

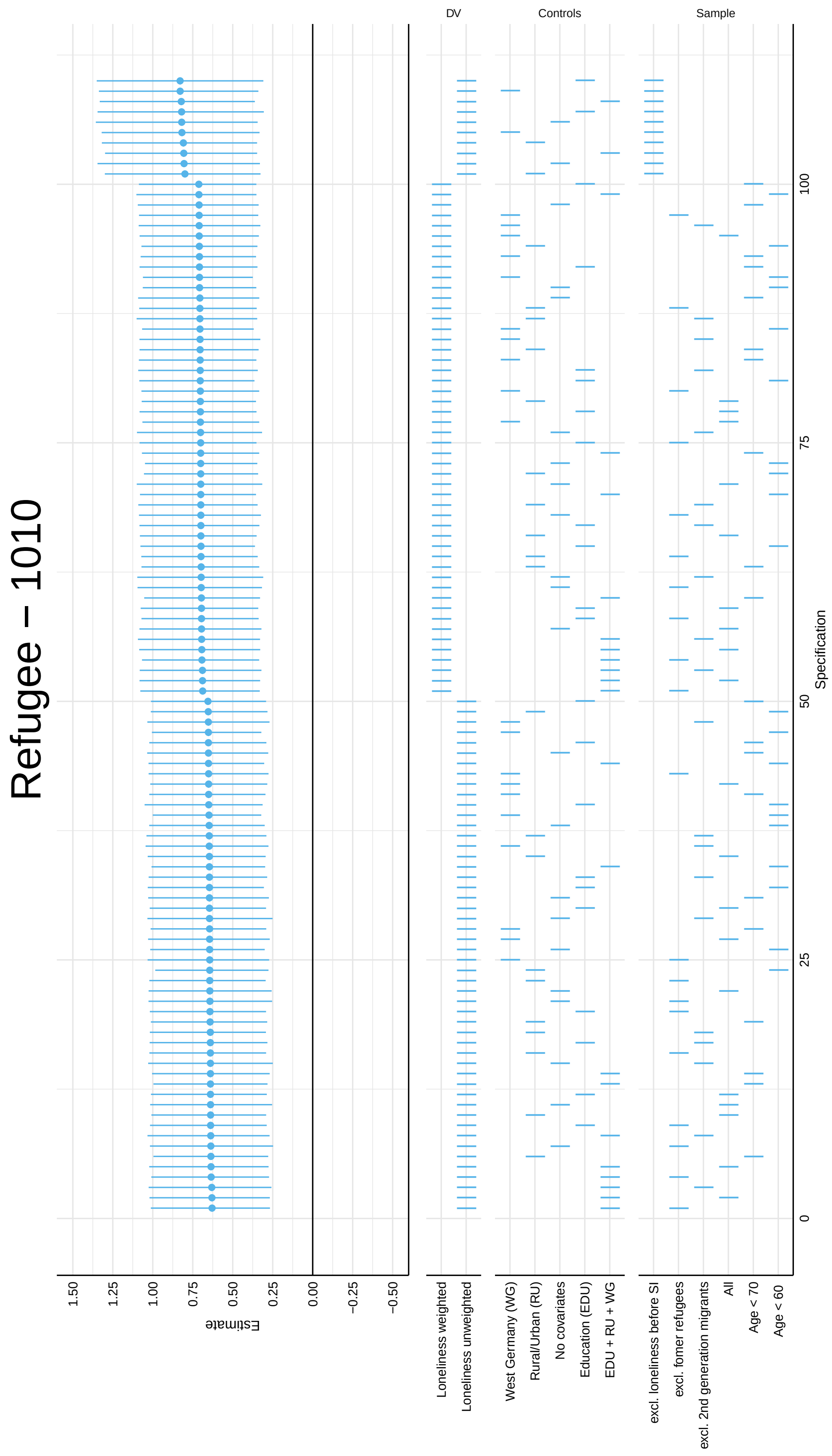
Refugee – 1010
DV
Controls
Sample
Estimate
1.50
1.25
1.00
0.75
0.50
0.25
0.00
−0.25
−0.50
Loneliness weighted
Loneliness unweighted
West Germany (WG)
Rural/Urban (RU)
No covariates
Education (EDU)
EDU + RU + WG
excl. loneliness before SI
excl. fomer refugees
excl. 2nd generation migrants
All
Age < 70
Age < 60
0
25
50
75
100
Specification

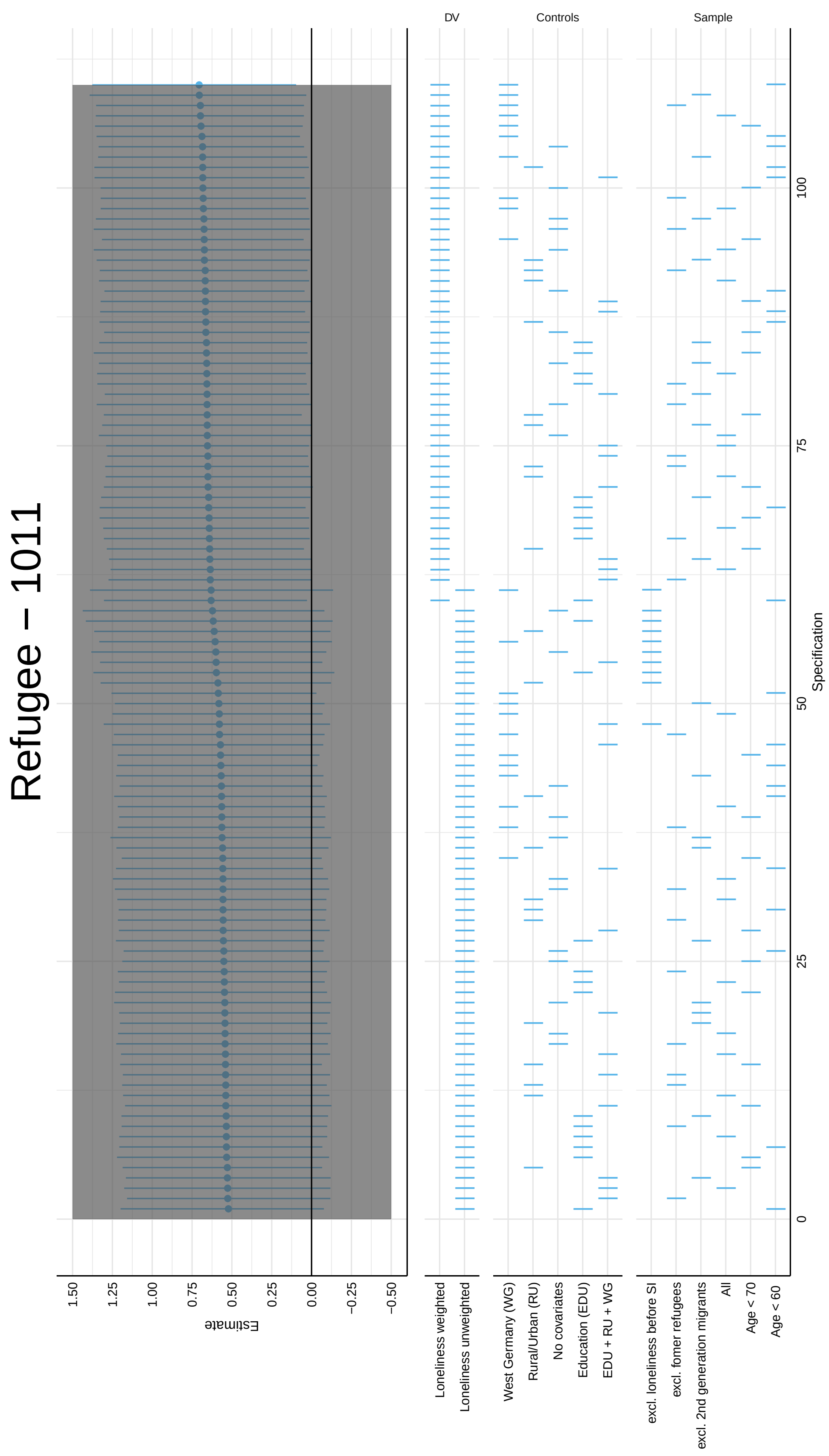

Refugee – 1011
Estimate
1.50
1.25
1.00
0.75
0.50
0.25
0.00
−0.25
−0.50
DV
Loneliness weighted
Loneliness unweighted
Controls
West Germany (WG)
Rural/Urban (RU)
No covariates
Education (EDU)
EDU + RU + WG
Sample
excl. loneliness before SI
excl. fomer refugees
excl. 2nd generation migrants
All
Age < 70
Age < 60
Specification
0
25
50
75
100

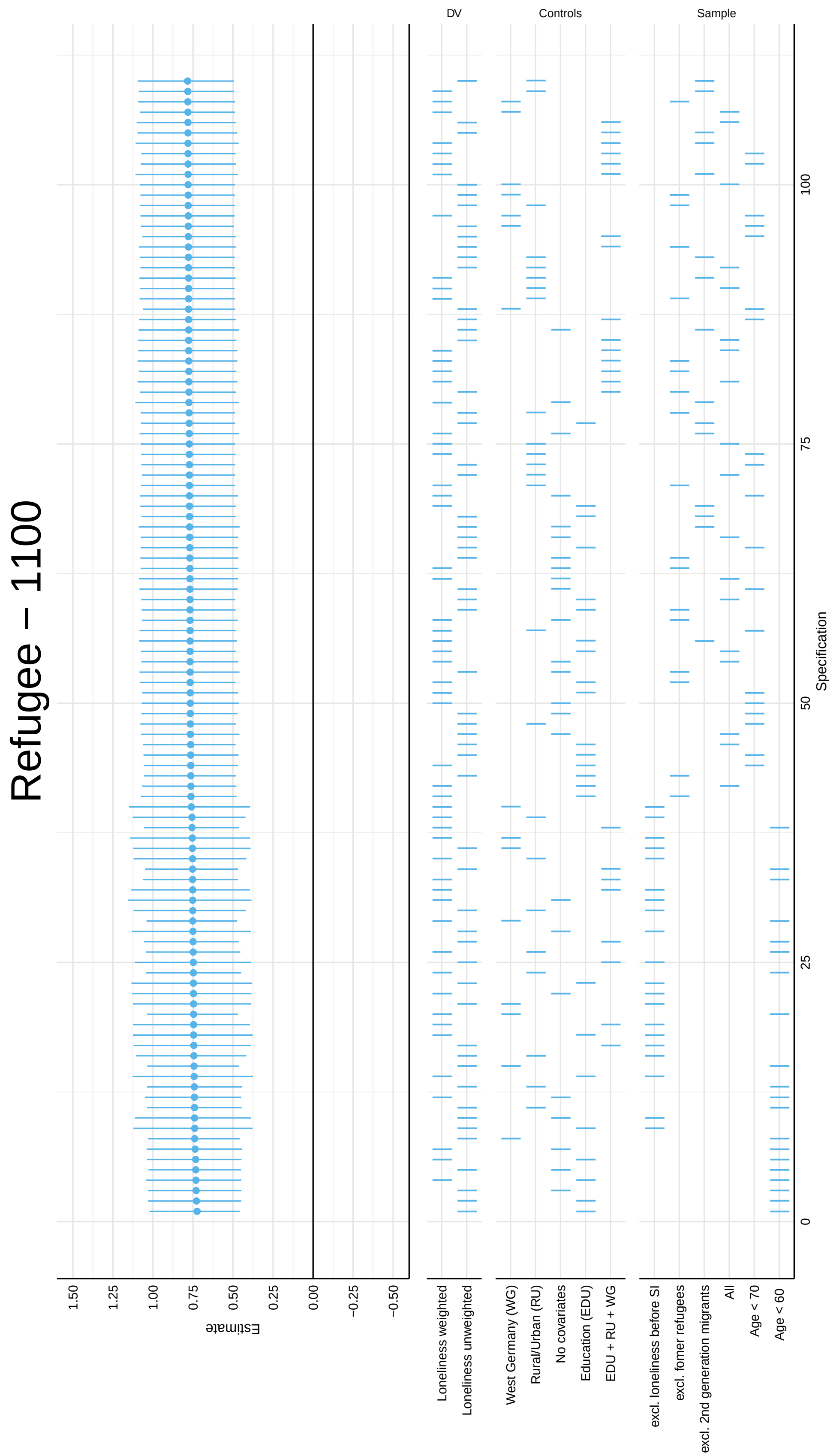
Refugee – 1100
Estimate
1.50
1.25
1.00
0.75
0.50
0.25
0.00
−0.25
−0.50
DV
Loneliness weighted
Loneliness unweighted
Controls
West Germany (WG)
Rural/Urban (RU)
No covariates
Education (EDU)
EDU + RU + WG
Sample
excl. loneliness before SI
excl. fomer refugees
excl. 2nd generation migrants
All
Age < 70
Age < 60
Specification
0
25
50
75
100

Refugee – 1101

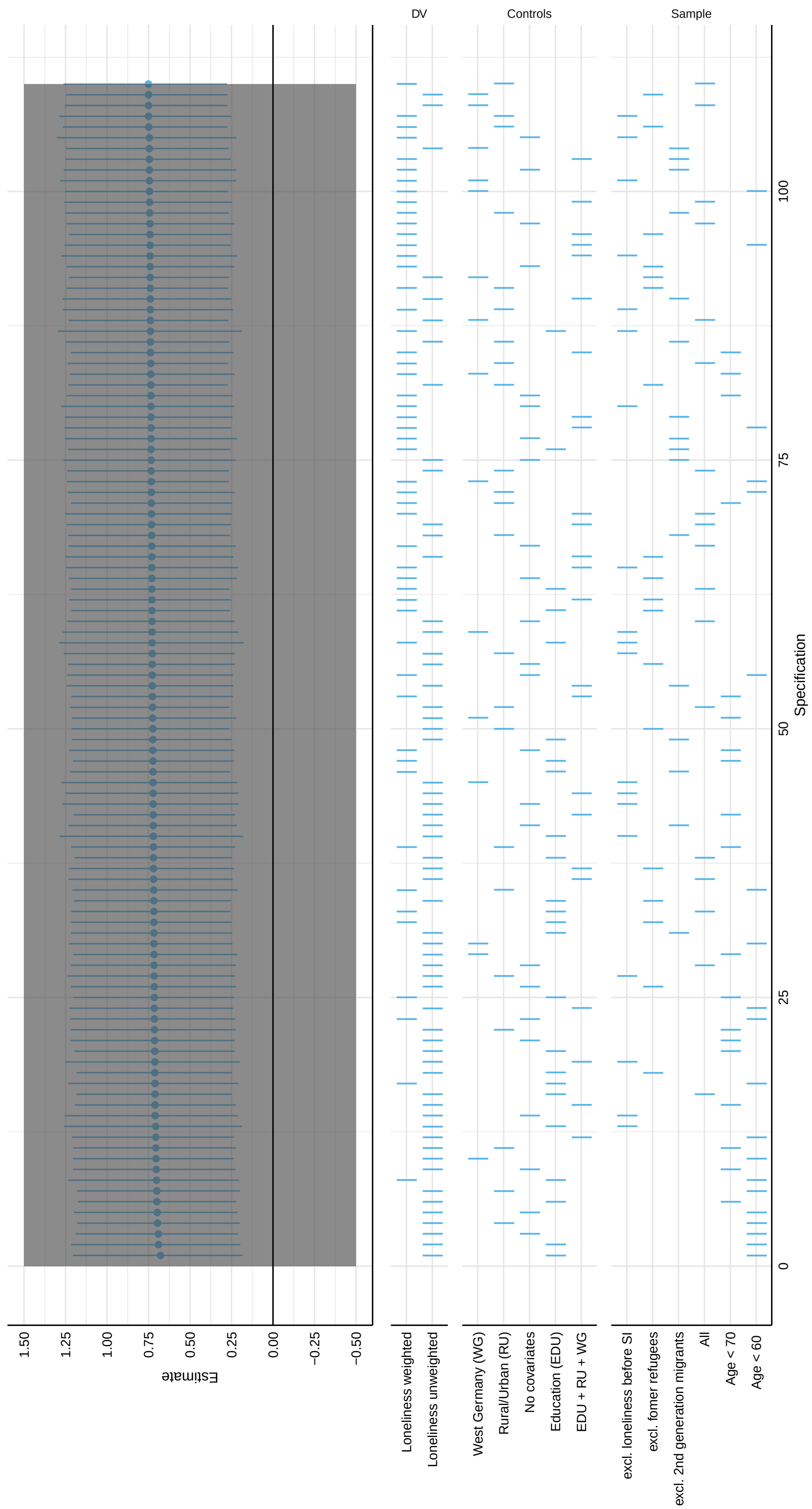

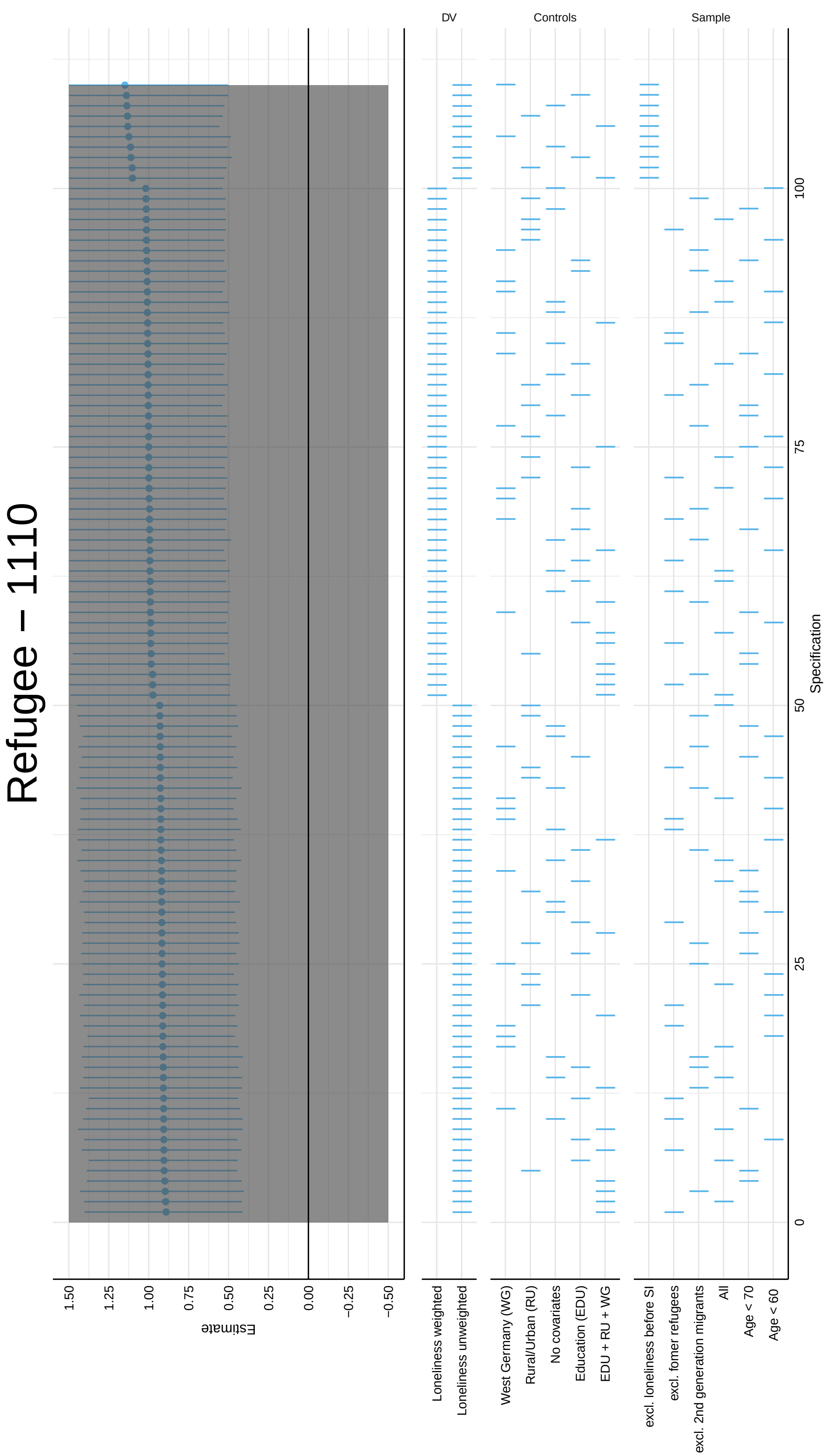
Refugee – 1110
Estimate
1.50
1.25
1.00
0.75
0.50
0.25
0.00
−0.25
−0.50
DV
Loneliness weighted
Loneliness unweighted
Controls
West Germany (WG)
Rural/Urban (RU)
No covariates
Education (EDU)
EDU + RU + WG
Sample
excl. loneliness before SI
excl. fomer refugees
excl. 2nd generation migrants
All
Age < 70
Age < 60
0
25
50
75
100
Specification

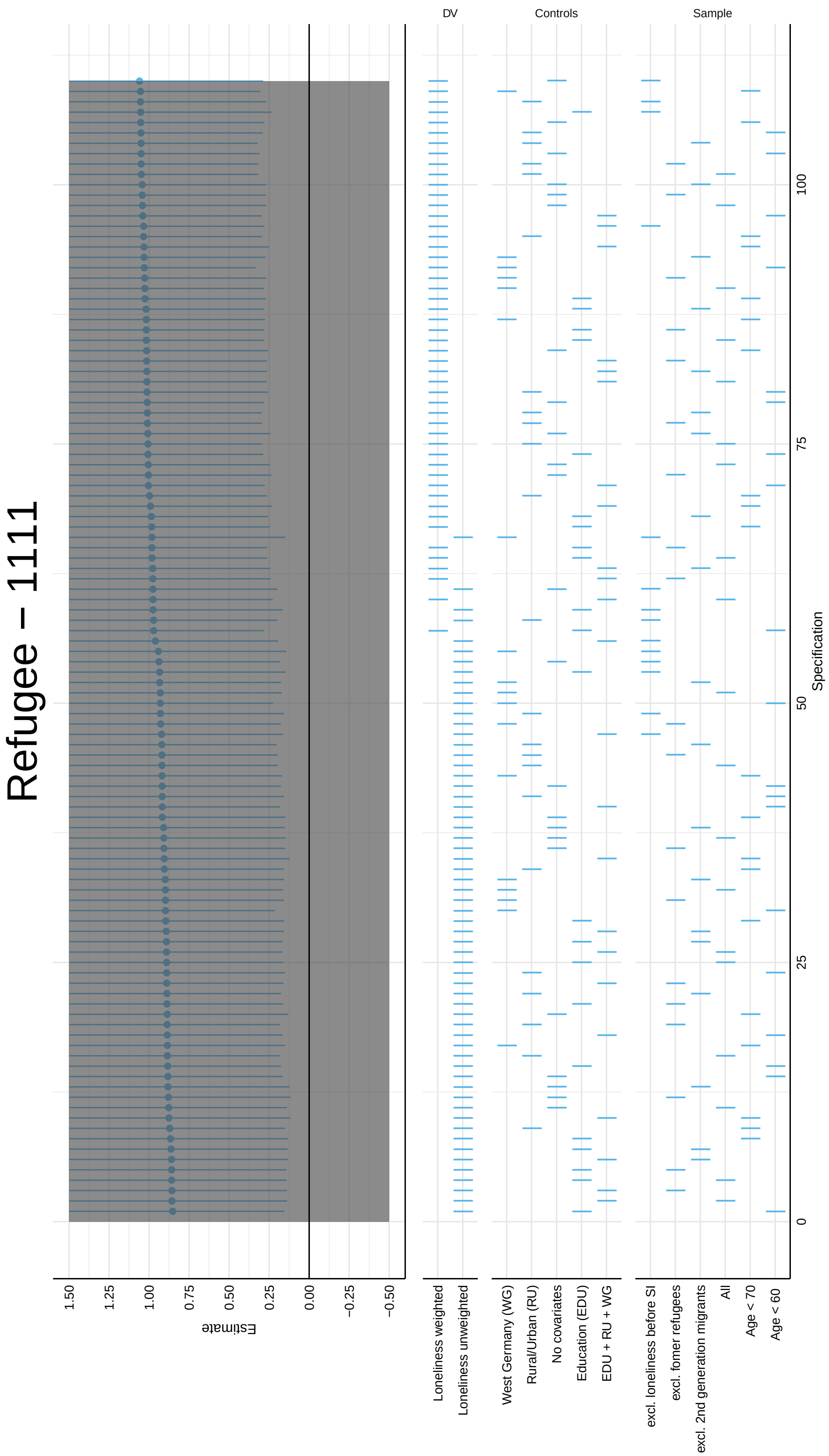

Refugee – 1111
Estimate
1.50
1.25
1.00
0.75
0.50
0.25
0.00
−0.25
−0.50
DV
Loneliness weighted
Loneliness unweighted
Controls
West Germany (WG)
Rural/Urban (RU)
No covariates
Education (EDU)
EDU + RU + WG
Sample
excl. loneliness before SI
excl. fomer refugees
excl. 2nd generation migrants
All
Age < 70
Age < 60
Specification
0
25
50
75
100
