# Supplementary material for: How Migration Status Shapes Susceptibility of Individuals’ Loneliness to Social Isolation
Source: Int J Public Health. 2022 Dec 6;67:1604576. doi: 10.3389/ijph.2022.1604576 (PMC9763294; doi:10.3389/ijph.2022.1604576)
Supplement: Supplementary file 2 [file DataSheet1.pdf]

International Journal of Public Health

Supplementary Material I

(online appendix)

It presents the results of all 48 specification curves for all three groups for all 16 codings of social isolation.

How migration status shapes susceptibility of individuals' loneliness to social isolation

Table of content

SM 1 - Loneliness – Measurement Invariance 3

Table S1a - Loneliness overall model fit by migration status 3

Table S1b - Loneliness factor loadings by migration status 4

Table S1c - LONE item intercepts by migration status 4

Table S2 - Test for measurement invariance of the loneliness across sex and migration status 4

Figure S1 – Loneliness (LONE) questionnaires used in the SOEP 6

Figure S2 - Factor structure for Loneliness (LONE). Refer to Figure S1 for item formulation in the survey and to Table S2 for summary statistics of the items 7

SM 2 - Coding decisions – general overview 8

Table S3 - Variable Coding Scheme and Survey Years 8

Table S4: Overview of years of measurement for social isolation and loneliness 11

Table S5: Sample restrictions 11

Table S6: Country of origin by migration status 11

Figure S3 – Distribution of the year of immigration within migrant and refugee group 12

Table S7: Descriptive statistics - control variables 13

SM 3 - Social isolation index cut off choices – in detail 14

Figure S4 – Coding scheme for social isolation 16

Figure S5 –Distribution of loneliness score by migration status 19

Figure S6 – Distribution of loneliness score by migration status 20

SM 4 – Choice of effect size threshold for contextual relevance hypothesis 21

Figure S7 – Relationship between support for H1 and effect size threshold 21

SM 5 - Multiverse analysis and discussion 23

Figure S8: Overview of specifications 24

SM 6 – Specification curve analysis 25

Figure S9: Bayesian model averaging over specifications for the estimates of standardized posterior mean and credible interval of the association between social isolation and loneliness – an illustrative example 26

SM 7 – Definition of the statistical model 27

SM 8 - Bayesian Evaluation of Informative Hypotheses (BEIH) 29

Table S8: Main results from figure 2 – Association of social isolation with loneliness 32

SM 9 – Theoretical considerations: The hypothesis under investigation and their theoretical origin 33

Competing hypotheses 34

Bibliography 38

SM 1 - Loneliness – Measurement Invariance

We ran measurement invariance analyses over the three groups of analyses in our study. Overall model fit is very good (based on CFI > 0.85 and RMSEA below < 0.1 criterion) in all three groups (Table S1a, for measurement quality adjusted cut-offs, see [1]). The model used is represented in figure S1. Comparing different models based on subsequently stronger restrictions, we can see that scalar measurement invariance holds across sex, age, and migration status (based on the *ΔCFI* and *ΔRMSEA* < 0.01 criterion [2,3]). This means that both the association of social isolation and loneliness as well as the levels of loneliness can be meaningfully compared across the three migration groups. The difference we find are therefore unlikely to derive from substantial differences in the way the three-item loneliness scale works in the three groups.

Table S1a - Loneliness overall model fit by migration status

	χ^2	*df*	*p*	*CFI*	*RMSEA*	*RMSEA-LB*	*RMSEA-UB*	*SRMR*
				**Loneliness**				
Overall	60.643	1.000	0.000	0.998	0.049	0.039	0.059	0.014
Migration status								
Host population	153.237	1.000	0.000	0.990	0.096	0.083	0.109	0.026
Migrant	29.033	1.000	0.000	0.992	0.086	0.061	0.114	0.024
Refugee	19.439	1.000	0.000	0.995	0.062	0.040	0.088	0.017

Table S1b - Loneliness factor loadings by migration status

	LONE-1	*LONE-2*	*LONE-3*
Overall	0.647	0.822	0.796
Migration status			
Host population	0.633	0.796	0.791
Migrant	0.640	0.825	0.787
Refugee	0.584	0.822	0.792

Table S1c - LONE item intercepts by migration status

	LONE-1	*LONE-2*	*LONE-3*
Overall	1.373	1.120	0.744
Migration status			
Host population	1.288	1.006	0.591
Migrant	1.457	1.142	0.821
Refugee	2.002	1.475	1.332

Table S2 - Test for measurement invariance of the loneliness across sex and migration status

						**Loneliness**						
						**Sex**						
	χ^2	*df*	*p*	*CFI*	*RMSEA*	*RMSEA-LB*	*RMSEA-UB*	*SRMR*	$\Delta\chi^2$	Δdf	*ΔCFI*	*ΔRMSEA*
configural	58.834	2.000	0.000	0.998	0.047	0.036	0.060	0.013	NA	NA	NA	NA
metric	62.894	3.000	0.000	0.998	0.040	0.030	0.050	0.015	4.060	1.000	0.000	-0.008
scalar	97.942	4.000	0.000	0.996	0.043	0.035	0.051	0.018	35.049	1.000	0.001	0.003
strict	111.969	5.000	0.000	0.996	0.041	0.034	0.048	0.018	14.027	1.000	0.001	-0.002

						**Migration status**						
configural	201.708	3.000	0.000	0.991	0.089	0.078	0.100	0.024	NA	NA	NA	NA
metric	301.836	5.000	0.000	0.987	0.084	0.075	0.093	0.038	100.128	2.000	0.004	-0.005
scalar	484.551	7.000	0.000	0.979	0.090	0.083	0.097	0.045	182.715	2.000	0.008	0.006
strict	925.451	9.000	0.000	0.959	0.110	0.104	0.116	0.051	440.901	2.000	0.020	0.020
						**Age categories**						
configural	137.446	12.000	0.000	0.995	0.069	0.058	0.082	0.015	NA	NA	NA	NA
metric	244.748	23.000	0.000	0.991	0.067	0.058	0.076	0.034	107.302	11.000	0.004	-0.003
scalar	298.632	34.000	0.000	0.989	0.060	0.053	0.067	0.037	53.884	11.000	0.002	-0.007
strict	382.531	45.000	0.000	0.986	0.059	0.053	0.065	0.039	83.899	11.000	0.003	-0.001

[a] *df* = degrees of freedom; *CFI* = comparative fit index; *RMSEA* = root mean square error of approximation; *LB* = lower bound; *UB* = upper bound. BRCS= Brief Resilience Coping Scale. n/a = not applicable.

Figure S1 – Loneliness (LONE) questionnaires used in the SOEP

321 How often do you feel that you miss the company of others?

Very often	1
Often	2
Sometimes	3
Occasionally	4
Never	5
No details	99

LONE-1

321:pgef11	bgp	bgpr321	Company of Others is missing
321:pgef11	bgp_refugees	bgpr321	Company of Others is missing
321:pgef11	pl	plj0587	Company of Others is missing

322 How often do you feel like an outsider?

Very often	1
Often	2
Sometimes	3
Occasionally	4
Never	5
No details	99

LONE-2

322:pgef12	bgp	bgpr322	Feeling Left Out
322:pgef12	bgp_refugees	bgpr322	Feeling Left Out
322:pgef12	pl	plj0588	Feeling Left Out

323 How often do you feel socially isolated?

Very often	1
Often	2
Sometimes	3
Occasionally	4
Never	5
No details	99

LONE-3

323:pgef13	bgp	bgpr323	Feeling Socially Isolated
323:pgef13	bgp_refugees	bgpr323	Feeling Socially Isolated
323:pgef13	pl	plj0589	Feeling Socially Isolated

[a] The questionnaires of SOEP are not copyrighted and free of charge [4]. Presented are the English translations of the German original questionnaires. Source: [5]

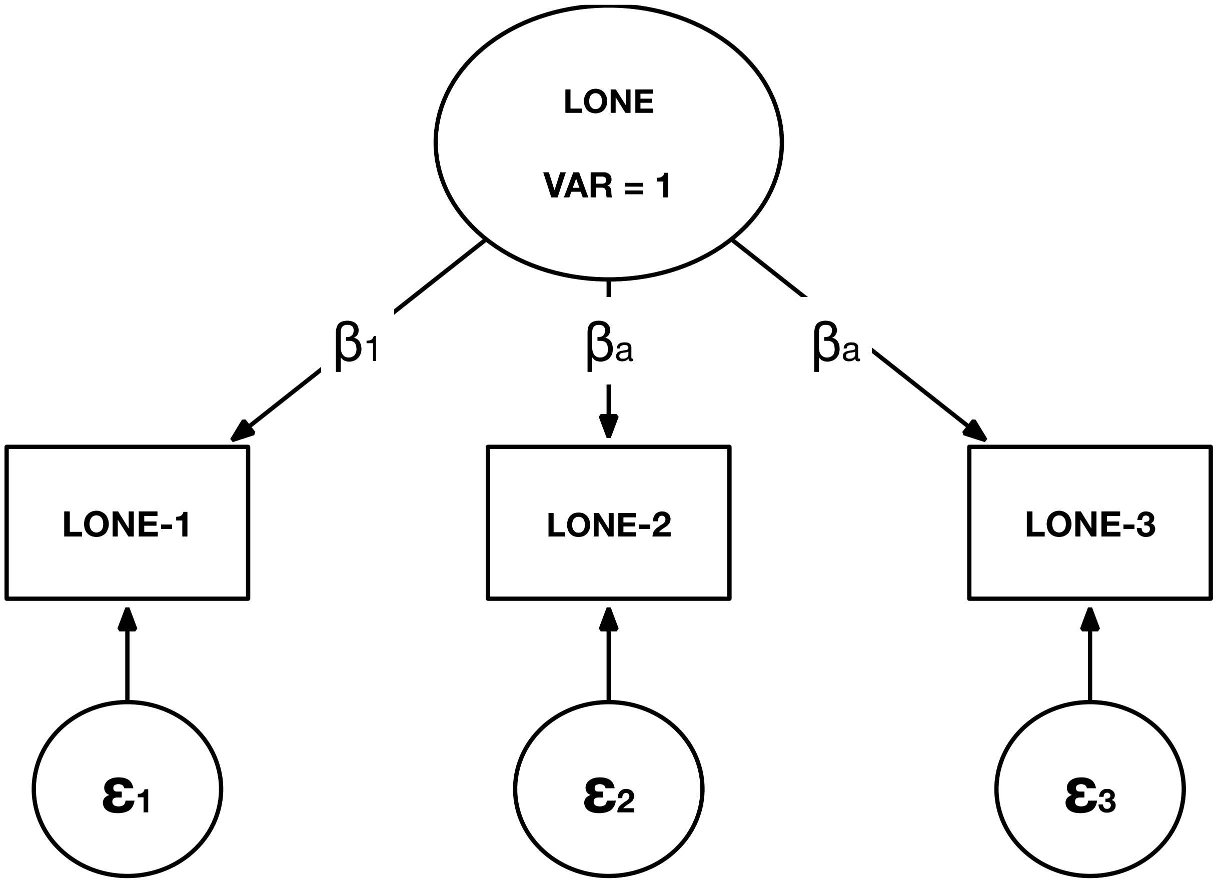

Figure S2 - Factor structure for Loneliness (LONE). Refer to Figure S1 for item formulation in the survey and to Table S2 for summary statistics of the items

SM 2 - Coding decisions – general overview

In the following, we present the coding of all variables used in the analysis, adding to the replication of this study.

Table S3 - Variable Coding Scheme and Survey Years

Variable	Coding Information	Survey Year
Independent Variables		
Network size – for emotional support	Count of the number of people with whom to discuss thoughts; Missing values in refugee population counted as 0 (not having a person).<br><br>This variable consists of five individual variables asking for the role relation providing emotional support. Each variable is re-coded as dummy – indicating whether a role relation was named.<br><br>Afterwards, the information is consolidated to one variable, indicating whether any emotional support is subjectively granted.<br><br>0 = no support<br>1 = support	2016 for host and migration population / 2017 for refugees of the IAB-BAMF-SOEP Survey of Refugees
Network size – informational support	Count of the number of people with whom to discuss educational and work-related matters; Missing values in refugee population counted as 0.<br><br>This variable consists of five individual variables asking for the role relation providing informational support. Each variable is re-coded as dummy – indicating whether a role relation was named.<br><br>Afterwards, the information is consolidated to one variable, indicating whether any informational support is subjectively granted.<br><br>0 = no support<br>1 = support	2016 for host and migration population / 2017 for refugees of the IAB-BAMF-SOEP Survey of Refugees
Network size – unpleasant truths	Count of the number of people with whom to discuss unpleasant truths; Missing values in refugee population counted as 0.<br><br>This variable consists of five individual variables asking for the role relation providing appraisal support. Each variable is re-coded as dummy – indicating whether a role relation was named.	2016 for host and migration population / 2017 for refugees of the IAB-BAMF-SOEP Survey of Refugees

	Afterwards, the information is consolidated to one variable, indicating whether any appraisal support is subjectively granted.<br><br>0 = no support<br>1 = support	
Spouse	0 = no spouse or partner<br>1 = spouse or partner	2017
Living Alone	0 = living not alone<br>1 = living alone	2017
Social Participation	Five dummy variables indicating the regular participation in social activities:<br>0 = not active<br>1 = active<br><br>Activities include: church, cultural activities, cinema/disco, sports, arts	2017
Dependent Variable		
Loneliness 1	Summary score of three items UCSL loneliness scale, coded 1 = never / 5 = very often: Company of others is missing, Feeling left alone, Feeling socially isolated	2017/ 2016 for those first surveyed in the IAB-BAMF-SOEP Survey of Refugees in 2016<br>*plj0587*<br>*plj0588*<br>*plj0589*
Loneliness 2	Weighted sum score of three items UCSL loneliness scale (subtraction of score from mean and divided by standard deviation)<br>[6]	2017/ 2016 for those first surveyed in the IAB-BAMF-SOEP Survey of Refugees in 2016<br>*plj0587*<br>*plj0588*<br>*plj0589*
Controls		
Age-Categories	Survey year – birthyear<br>Categorization:<br>1 = 18 - 24 years<br>2 = 25 - 29 years<br>3 = 30 - 34 years<br>4 = 35 – 39 years<br>5 = 40 – 44 years<br>6 = 45 – 49 years<br>7 = 50 – 54 years<br>8 = 55 – 59 years<br>9 = 60 – 64 years<br>10 = 65 – 69 years	2017

	11 = 70 – 74 years<br>12 = 75+ years	
Gender	0 = male<br>1 = female	2017
Education	CASMIN<br>1 = Inadequately Completed<br>2 = General Elementary School<br>3 = Basic Vocational Qualification<br>4 = Intermediate General Qualification<br>5 = Intermediate Vocational<br>6 = General Maturity Certificate<br>7 = Vocational Maturity Certificate<br>8 = Lower Tertiary Education<br>9 = Higher Tertiary Education	2017
Rural/ Urban divide (Population density)	Number of inhabitants - retrieved from BGSR classification of counties in Germany<br>1 = > 2000<br>2 = 2000 - 5000<br>3 = 5000 - 20000<br>4 = 20000 - 50000<br>5 = 50000 -100000<br>6 = 100000 - 500000<br>7 = 500000 +	2017
Migration status	Individuals identified and categorised based on the generated SOEP variable *migback* and by means of the refugee sample indicators[a]<br>1 = Native Germans – individuals with German nationality or residence status, who were born in Germany and whose parents were no migrants either [7][b]<br>2 = Migrants – in this case those with a direct migration background, meaning they were born outside of the German borders after 1949.<br>3 = Refugee status – individuals who applied for asylum between 2013 and 2016 (selection via sample M3, M4 and M5 of the IAB-BAMF-SOEP Survey of Refugees)	2017

[a] For one sample specification, we do not select refugees via the IAB BAMF SOEP Survey (Refugees who applied for asylum in Germany between 2013 and 2016) but also those who applied earlier. Those individuals can be identified using the generated *arefback* variable in the SOEP.

[b] For one specification of the model, we include second-generation migrants to the group of native Germans.

Table S4: Overview of years of measurement for social isolation and loneliness

Group	Loneliness	Social isolation
Host	2017	2016
Migrant	2017	2016
Refugee	2016/2017	2017

Table S5: Sample restrictions

	Restriction	Individuals
1	Full sample	69 766
2	Exclude non-refugees, pre-2013 refugees, and German born refugees in refugee sample	67 996
3	Exclude new 2017-sample (PIAAC-L) without support variables	62 331
4	Exclude non-probability observations	45 398
5	Exclude individuals below 18	30 628
6	Exclude individuals with missing information on covariates	28 569
7	Exclude individuals with missing information on loneliness	25 171

Table S6: Country of origin by migration status

Country of origin	Host	Migrant	Refugee
Germany	16 658	0	0
Turkey	0	311	6
Poland	0	441	0
Syria	0	288	0
Romania	0	54	2569
Syria	0	435	70
Russia	0	21	569
Afghanistan	0	58	633
Iraque	0	226	33
Ex-Yugoslavia	0	464	48
Southern Europe	0	386	11
Eastern Europe	0	484	43
CIS	0	93	116
Arabic	0	62	1
Latin America	0	243	3
Western Europe & Northern America	0	63	371
Asia	0	150	249
Rest of the world	0	11	1
**Total**	16 658	3790	4723

Figure S3 – Distribution of the year of immigration within migrant and refugee group

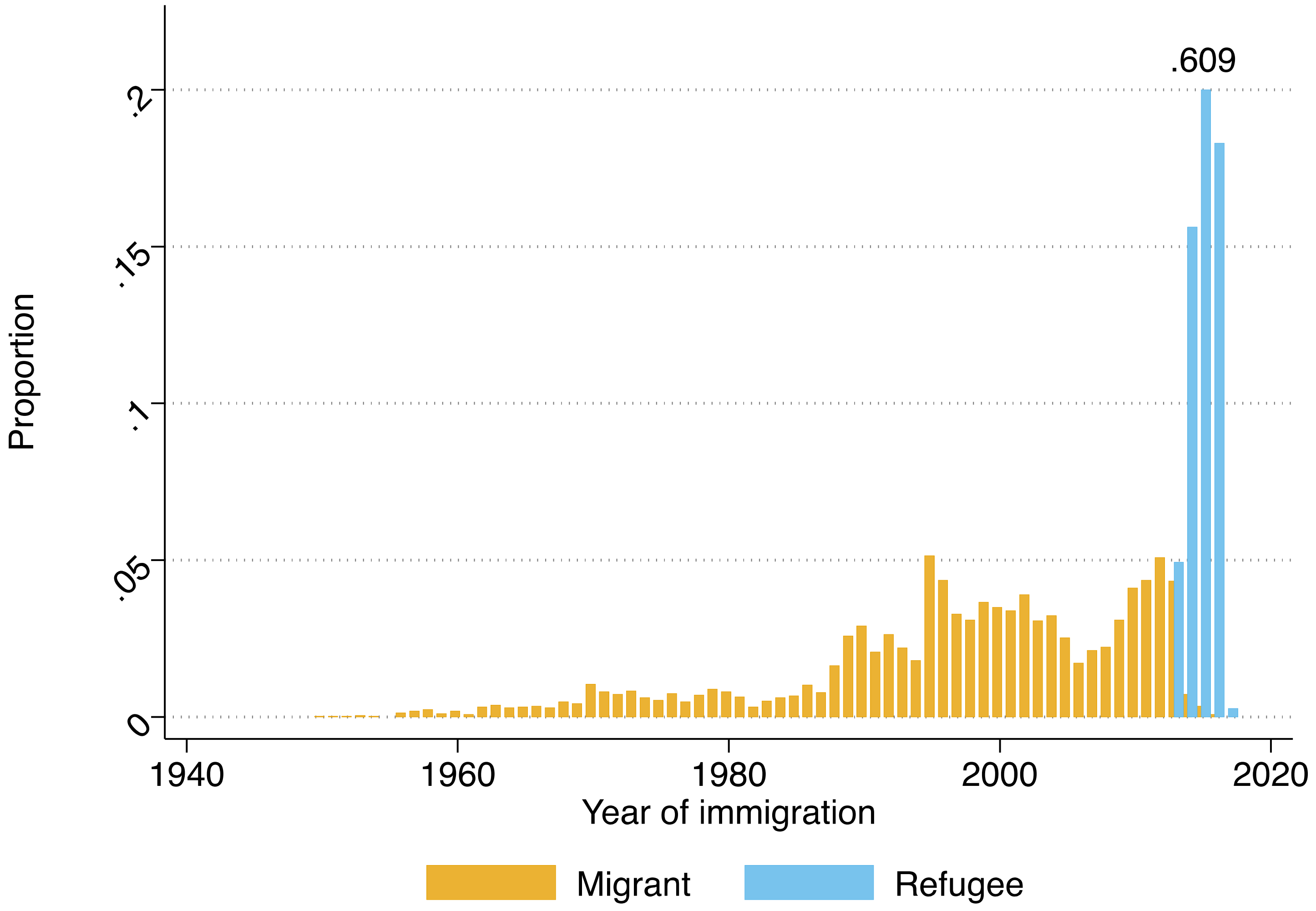

[a] Value of year 2015 for refugees (0.609) is cut off at 0.2 for illustrative purposes. It means that 60.9% of all refugees in the analytic sample arrived in Germany in 2015.

Table S7: Descriptive statistics - control variables

Migration status	Host				Migrant				Refugee			
	M	SD	Min	Max	M	SD	Min	Max	M	SD	Min	Max
Loneliness score (weighted)	-0.17	0.85	-1.28	3.33	0.04	0.97	-1.28	3.33	0.57	1.24	-1.28	3.33
Loneliness score (unweighted)	2.88	2.22	0.00	12.00	3.42	2.53	0.00	12.00	4.81	3.23	0.00	12.00
Gender (proportion of men)	0.46	0.50	0.00	1.00	0.45	0.50	0.00	1.00	0.61	0.49	0.00	1.00
Age	51.18	17.47	18.00	101.00	44.35	13.91	18.00	98.00	33.40	10.99	18.00	97.00
**Social Isolation by coding**												
1111	0.00	0.05	0.00	1.00	0.00	0.04	0.00	1.00	0.00	0.06	0.00	1.00
0111	0.02	0.15	0.00	1.00	0.01	0.12	0.00	1.00	0.04	0.19	0.00	1.00
1110	0.01	0.07	0.00	1.00	0.00	0.06	0.00	1.00	0.01	0.12	0.00	1.00
0110	0.06	0.24	0.00	1.00	0.04	0.20	0.00	1.00	0.11	0.31	0.00	1.00
1101	0.00	0.06	0.00	1.00	0.00	0.05	0.00	1.00	0.01	0.09	0.00	1.00
0101	0.04	0.19	0.00	1.00	0.03	0.16	0.00	1.00	0.08	0.28	0.00	1.00
1100	0.01	0.09	0.00	1.00	0.01	0.09	0.00	1.00	0.03	0.17	0.00	1.00
0100	0.11	0.31	0.00	1.00	0.10	0.29	0.00	1.00	0.24	0.42	0.00	1.00
1011	0.01	0.08	0.00	1.00	0.00	0.06	0.00	1.00	0.01	0.08	0.00	1.00
0011	0.05	0.22	0.00	1.00	0.04	0.20	0.00	1.00	0.08	0.28	0.00	1.00
1010	0.02	0.13	0.00	1.00	0.01	0.11	0.00	1.00	0.03	0.16	0.00	1.00
0010	0.10	0.30	0.00	1.00	0.10	0.31	0.00	1.00	0.19	0.39	0.00	1.00
1001	0.01	0.09	0.00	1.00	0.01	0.08	0.00	1.00	0.02	0.13	0.00	1.00
0001	0.07	0.26	0.00	1.00	0.07	0.25	0.00	1.00	0.14	0.35	0.00	1.00
1000	0.02	0.16	0.00	1.00	0.02	0.15	0.00	1.00	0.06	0.24	0.00	1.00
0000	0.15	0.35	0.00	1.00	0.16	0.36	0.00	1.00	0.30	0.46	0.00	1.00
**Education**												
Inadequately Completed	0.01	0.10	0.00	1.00	0.06	0.24	0.00	1.00	0.41	0.49	0.00	1.00
General Elementary School	0.06	0.24	0.00	1.00	0.15	0.36	0.00	1.00	0.21	0.40	0.00	1.00
Basic Vocational Qualification	0.22	0.41	0.00	1.00	0.20	0.40	0.00	1.00	0.02	0.13	0.00	1.00
Intermediate General Qualification	0.03	0.17	0.00	1.00	0.01	0.11	0.00	1.00	0.00	0.05	0.00	1.00
Intermediate Vocational	0.27	0.45	0.00	1.00	0.06	0.23	0.00	1.00	0.00	0.01	0.00	1.00
General Maturity Certificate	0.04	0.20	0.00	1.00	0.08	0.28	0.00	1.00	0.15	0.36	0.00	1.00
Vocational Maturity Certificate	0.09	0.29	0.00	1.00	0.16	0.37	0.00	1.00	0.03	0.16	0.00	1.00
Lower Tertiary Education	0.09	0.29	0.00	1.00	0.02	0.15	0.00	1.00	0.00	0.00	0.00	0.00
Higher Tertiary Education	0.16	0.37	0.00	1.00	0.24	0.43	0.00	1.00	0.18	0.39	0.00	1.00
West vs. East Germany	0.75	0.43	0.00	1.00	0.92	0.27	0.00	1.00	0.87	0.34	0.00	1.00
**Population density**												
GKK > 2.000	0.06	0.24	0.00	1.00	0.02	0.15	0.00	1.00	0.02	0.12	0.00	1.00
GKK 2000 - 5000.	0.10	0.30	0.00	1.00	0.05	0.22	0.00	1.00	0.06	0.23	0.00	1.00
GKK 5000 - 2000	0.29	0.45	0.00	1.00	0.24	0.43	0.00	1.00	0.23	0.42	0.00	1.00
GKK 20000 - 50000	0.18	0.38	0.00	1.00	0.21	0.41	0.00	1.00	0.22	0.42	0.00	1.00
GKK 50000 - 100000	0.09	0.28	0.00	1.00	0.09	0.29	0.00	1.00	0.12	0.33	0.00	1.00
GKK 100000 - 500000	0.13	0.34	0.00	1.00	0.17	0.37	0.00	1.00	0.20	0.40	0.00	1.00
GKK 500000 +	0.15	0.36	0.00	1.00	0.22	0.41	0.00	1.00	0.15	0.36	0.00	1.00
Observations	16658				3790				4723			

SM 3 - Social isolation index cut off choices – in detail

We base the construction of social isolation on the seminal work on the need to belong by Baumeister and Leary (1995)[8]. The authors set out two principles for the sense of belonging that guide our definition of social isolation. First, the principle of *satiation* refers to the need for a minimum level of social connectedness to be present. This means that individuals evaluate themselves as lonely primarily if a certain degree of social connectedness is not present. It implies a threshold effect of social connectedness on loneliness. From this principle we derive relevance of analyzing social isolation, as a categorical concept, instead of degrees of social connectedness. Second, the *substitution* principle refers to the idea that certain social connections can replace others, hence shielding from isolation to a certain extend. This is reflected in the composition of this variable.

Social connections can cover different dimensions of social life, for example family and household, social activities, or social support [9]. If an individual lacks these social linkages within a certain dimension, we will define the individual as being *deprived* in this particular social dimension in contrast to being *integrated*. If individuals are deprived in several dimensions, thereby not satisfying the *satiation* criterion, we will consider them to be overall socially isolated.

Concretely, we measure social isolation across three domains consisting of several indicators [9]:

1. the size of the support network (SS) as surveyed by means of the number of individuals named in a name generator on social support in three categories. The SOEP contains the social support items (SS) for refugees in 2017 and for the host population and other migrants in 2016. Hence, we transmit the 2016 information for SOEP participants to 2017.

2. living and partnership arrangements (LA) a) having a spouse and b) presence of other household members.
3. frequency of attending social activities (SA) a) church, b) cultural activities, c) cinema/disco, d) sports, e) arts.

As there are different reasonable thresholds, which can be used to define social isolation across the different social domains, we create different variants of our indicator. These cut offs vary across the degree of substitution that can be integrated within a dimension. For instance, we identify a deprivation in the dimension of social support networks first, when someone has named no one as social support provider. In a second variation, we consider someone as deprived who only names 1 person per dimension. Variation one allows full substitutability, the other one partial substitutability. Moreover, we assume the absence of substitution theoretical implausible as it would lead to empirical extremely high levels of social isolation. For each indicator and domain as well as the final addition of domains we test two alternative cut offs: one that allows for more and another allowing for less substitution. Overall, we derive 16 different social isolation indicators based on both principles.

Figure S4 – Coding scheme for social isolation

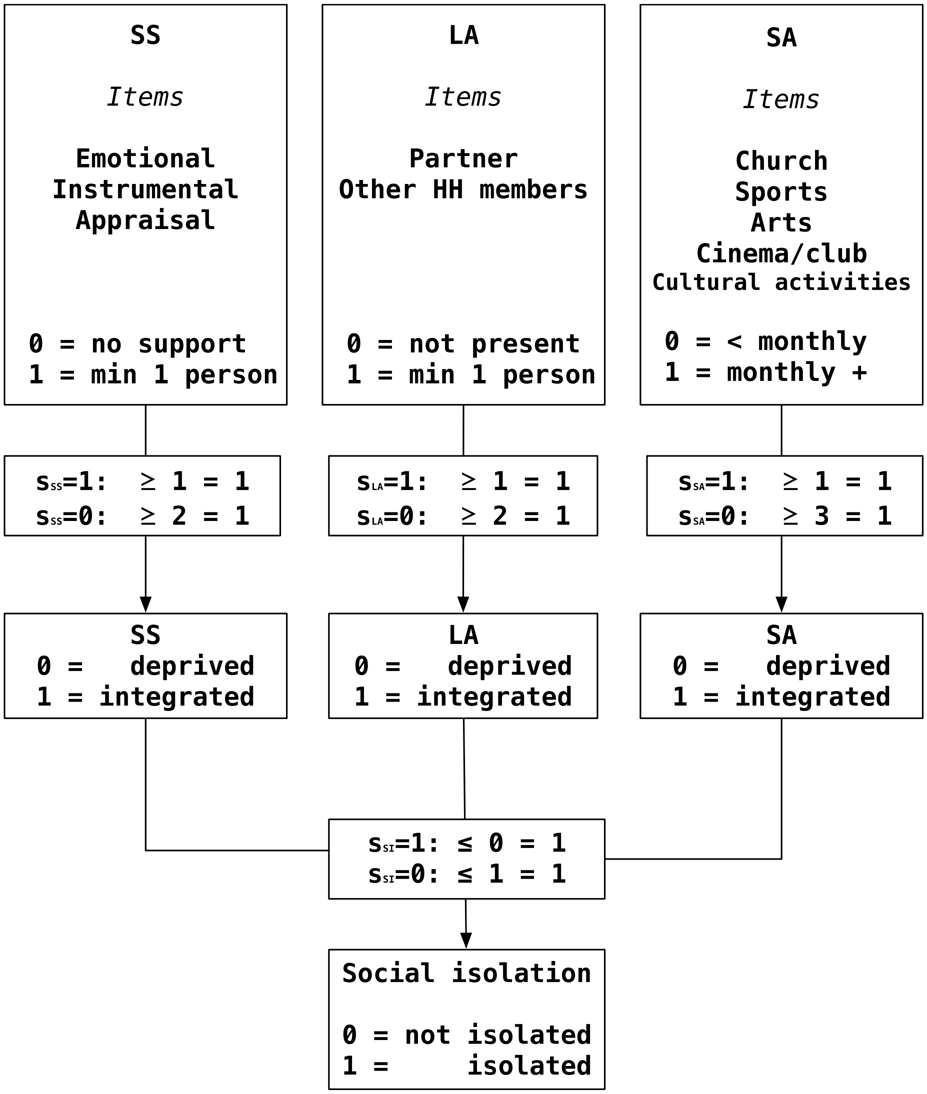

[a] The numbers 0 and 1 in the summary stages of indicators refer to s = 1: full substitutability. s = 0: partial substitutability. They are also part of Figure 2 below to signal the combinations of partial and full substitution. SS= social support, LA= living and partnership arrangements, SA= social activities, HH= household.

Figure S4b – Visualization of the binary coding scheme explaining the combination of cut offs for the social isolation indicator.

s_{SI}	s_{SS}	s_{LA}	s_{SA}
1	1	1	1
1	1	1	0
1	1	0	1
1	1	0	0
1	0	1	1
1	0	1	0
1	0	0	1
1	0	0	0
0	1	1	1
0	1	0	1
0	1	0	0
0	0	1	1
0	0	1	0
0	0	0	1
0	0	0	0

Overall, the different coding strategies across all domains and indicators leaves us with 16 alternative social isolation variables.

$$I_{js_j} = D[0,1], D = 1 \mid \sum_{k_j=1}^{k_j} C_{k_j} \geq t_{s_j}, j \in \{SS, LA, SA\}, s_j \in \{1,0\} \quad (1)$$

The D_{js} indicates the state of integration (1) versus deprivation (0) in dimension j applying threshold t_{s_j} which either allows for fully substitutability ($s_j = 1$) or only partial substitutability $s_j = 0$. k_j indicates the number of indicators C_{k_j}used to measure social connections in each dimension. Based on this coding of integration per dimension, social isolation is then defined as:

$$SI_{s_j} = D[0,1], D = 1 \mid \sum_{m=1}^{3} I_{js_j} \leq t_{s_{SI}}, j \in \{SS, LA, SA\}, s_{SI} \in \{1,0\} \quad (2)$$

This means that an individual is defined as being social isolated if the number of dimensions it is integrated into is equal or lower than a certain threshold $\mathrm{t}_{\mathrm{s_{si}}}$.

Figure S5 –Distribution of loneliness score by migration status

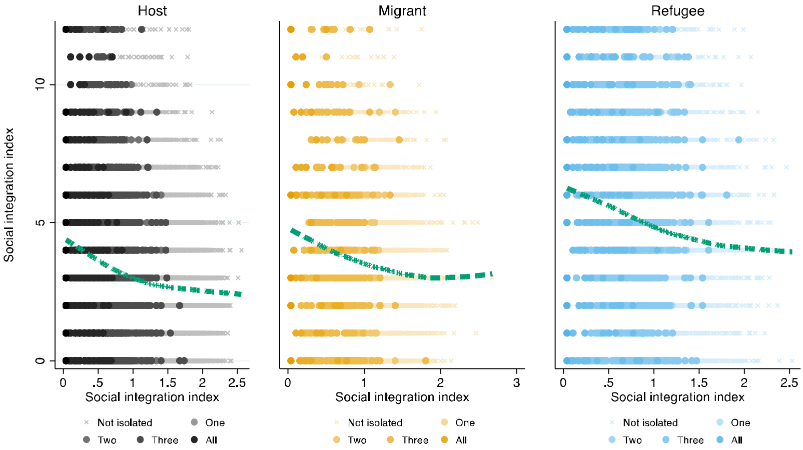

Note: Observed data points are plotted over the loneliness index and an index of social integration. The different shadings indicate under which coding the observation is considered to be socially isolated. Listed are the number of dimensions in which full substitution is allowed. As noted in the manuscript, the more substitution is allowed, the fewer observations are isolated. The dashed line shows the functional relationship between social integration and loneliness estimated by non-parametric LOESS regression.

Figure S6 – Distribution of loneliness score by migration status

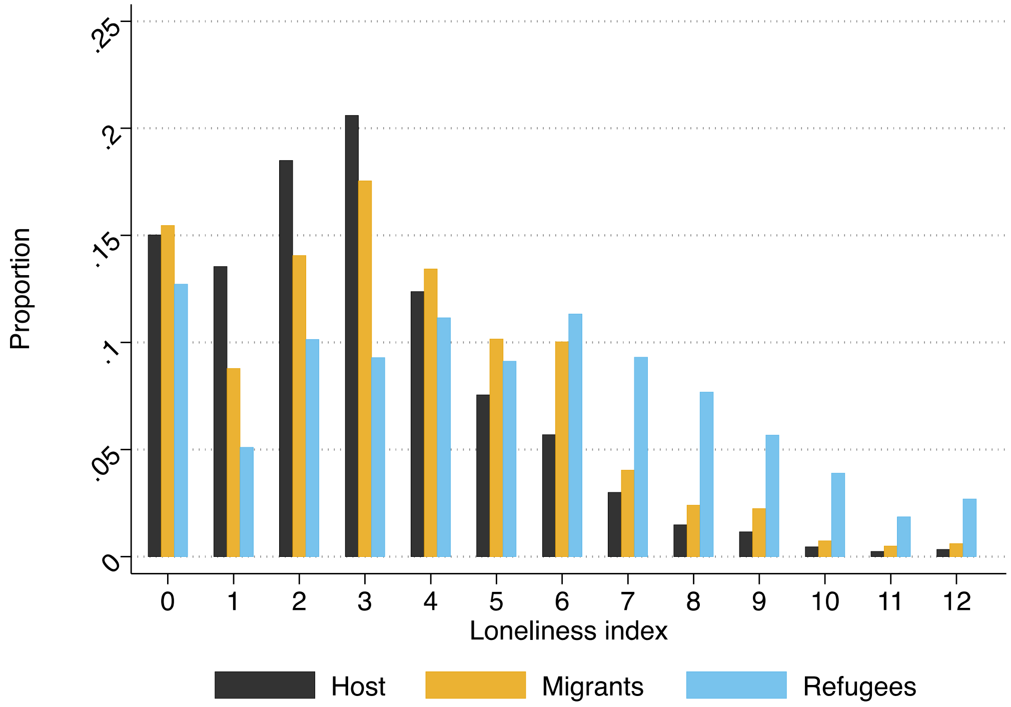

SM 4 – Choice of effect size threshold for contextual relevance hypothesis

Figure S7 – Relationship between support for H1 and effect size threshold

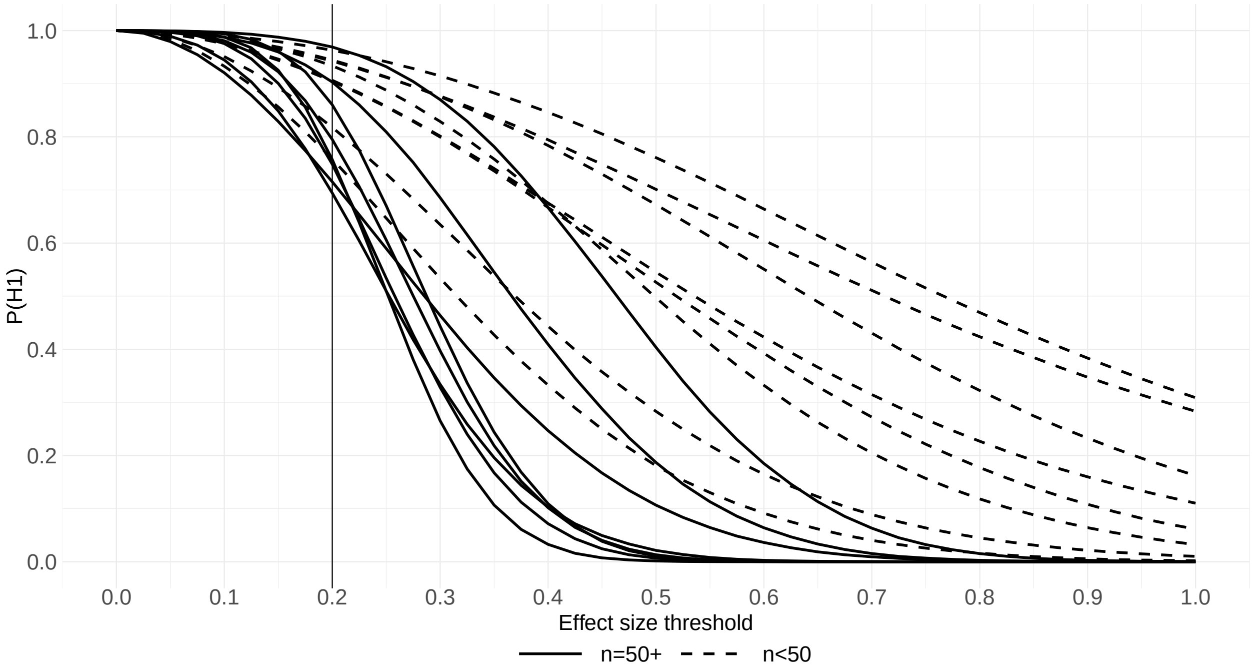

Note: The figure shows the relationship between choice of the effect size threshold and support for *contextual relevance* hypothesis H_1. for all 16 codings (divided into those with sufficient and those with non-sufficient minimum number of observations in the social isolation category).

With respect to the effect size threshold chosen in H_1, one could of course consider other values for this cut-off. Figure S6 shows the relationship between this choice and the degree of support H_1 would get using our data and model.

The higher the threshold is chosen (meaning only very large differences in effect size are considered to be consequential), the less support H_1 gets. We chose a reference cut-off of 0.2 SD, because this value about the size of the maximum difference in loneliness that is found between different age groups (age range 20 to 80) in a previous study using German data [10]. It is therefore a cut-off that considers only substantial differences (approximately as large or larger than strongest differences found across all age) as evidence in favor of H_1. Consequently, the strong support we find for H1 corroborates the visual impression of figure 3

in the manuscript that the differences between the migrant group and the host and refugee group are indeed substantial in size and meaningful.

SM 5 - Multiverse analysis and discussion

Recent research proposes that studies based on secondary data analysis report all plausible specifications of their data coding and sample definitions [11,12]. It reduces the probability of reporting findings, specific to certain idiosyncratic decisions in the process of the data analysis [13,14]. Based on the definition of social isolation and the different cut offs presented additionally to alterations in sample definition and coding, we report all plausible specifications in a multiverse framework (specifications are listed in Figure S6).

As the section on the social isolation index indicates, researchers not only take decisions on how to construct different measures of a concept. Through these decisions, they are able to have influence on the results presented.

In our analyses, we take several major coding decisions. In the following, we explain these different decisions. After that, we present the analyses with all the varying decisions taken to show how our results vary by specification. This method showing the volatility or robustness of results is called a multiverse analysis, as prominently features amongst others in the work by Rohrer et al. (2017).

Figure S8 shows four different areas in which this article sets out different specifications, leaving us with a theoretical set of 1920 sample specifications to test in the analysis.

1) We present two different coding schemes for the UCLA Loneliness Scale.
2) We test 5 different specifications regarding control variables: besides always controlling for gender and age, we test for (a) east/west differences, (b) leaving out any covariates, (c) size of population in the area, (d) education levels, and (e) all indicators combined.

3) We test 5 different sample restrictions: (a) leaving out individuals who applied for asylum before 2013, (b) leaving out second generation migrants, (c) including all individuals from the 2017 SOEP survey, (d) leaving out individuals age < 54, (e) leaving out individuals age < 65

4) Due to the different possible cut offs, 16 different social isolation indicators are derived in preparation of the analysis.

Figure S8: Overview of specifications

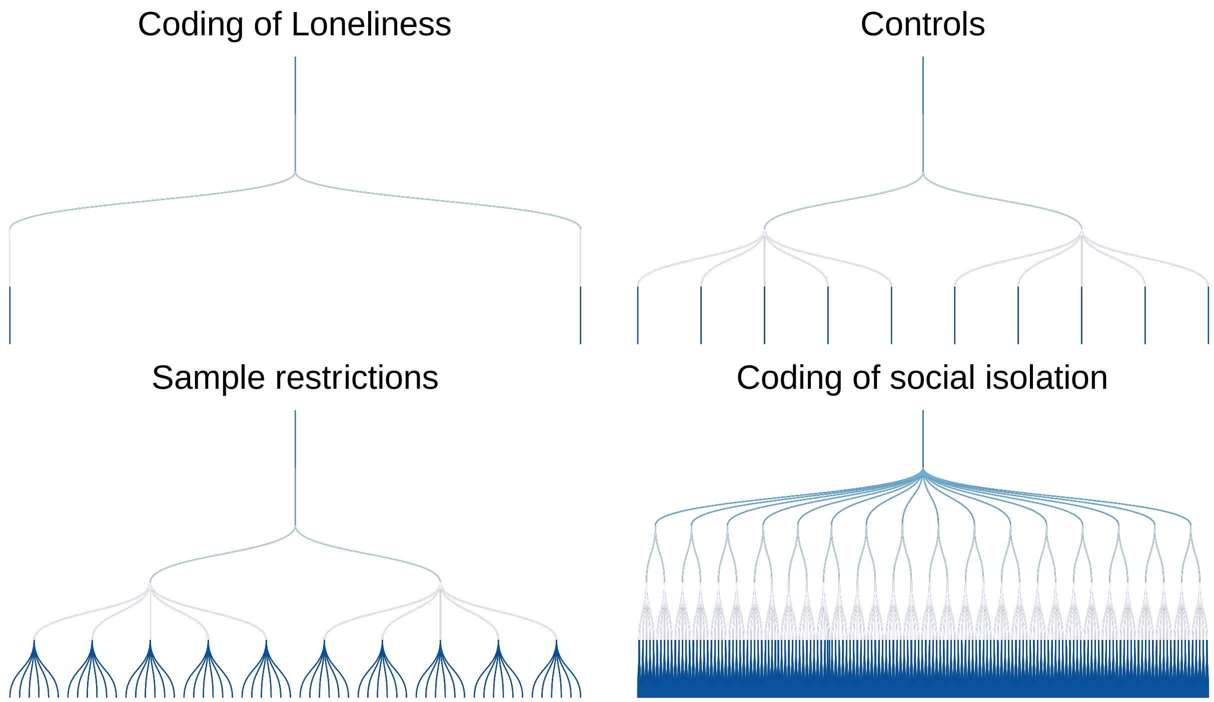

SM 6 – Specification curve analysis

The derived posterior means and credible intervals in the manuscript are not the indicators we are most interested about in our analysis. Aim of this study is to retrieve Bayes factors and Posterior Model probabilities that allow for a comparison of hypotheses. Unfortunately, it is difficult to derive these estimates with 1887 converged results per group of interest. 33 of the total 1920 models did not converge.

Hence, we have summarized the results from the multiverse framework further, along the lines of the 16 social isolation indicators.

Figure 4 of the manuscript shows the standardized effect sizes of the association between social isolation and loneliness on the X-axis. Meanwhile, the Y-axis provides us with the 16 different alternative social isolation indicators used. Per indicator, we present one posterior mean and credible interval per group.

How does one arrive at only three outcomes when in fact we started out with results from 1887 model specifications? Figure S8 shows how we created an overall mean from the posterior mean and the credible interval of all specifications for the social isolation indicator included in the model. The method allows discussing the differences in association depending on the specification of the social isolation index without going too much into detail about the possible other specifications.

In this example below, the graph shows the size of the association for a model specification where social isolation is coded with a soft cut of in the social network domain, a hard cut off in the living arrangement domain, a soft cut off in the social activities domain and a soft cut off for the summary of the overall domains.

Figure S9: Bayesian model averaging over specifications for the estimates of standardized posterior mean and credible interval of the association between social isolation and loneliness – an illustrative example

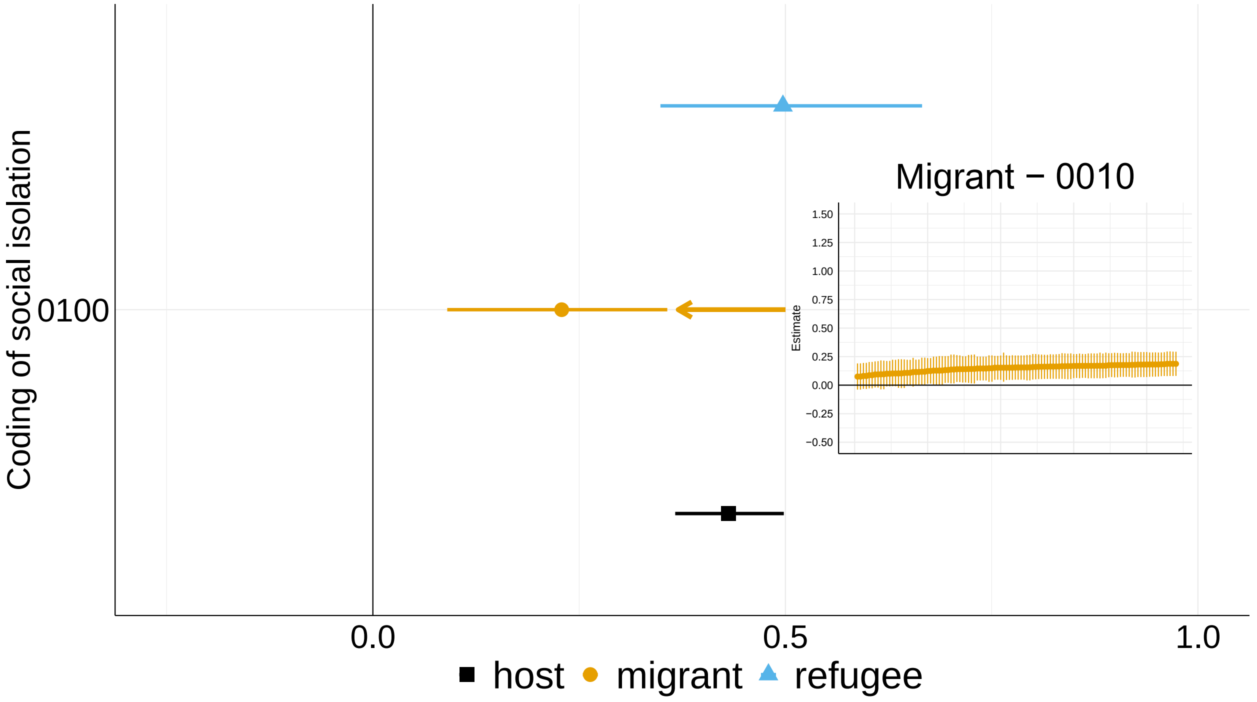

SM 7 – Definition of the statistical model

This paper comprises five competing hypotheses, postulating different associations between the groups of host (H), migrant (M) and refugee (R) population living in Germany in terms of their objective social isolation and loneliness. The central parameters that represent the quantities of interest from our hypotheses are the regression coefficients β. They estimate the association of social isolation (SI) and loneliness (LONE) for the host population, migrants, and refugees separately, conditional on a set of control variables (X).

$$LONE_{gsdi} = \beta_{gsd} SI_{gsdi} + \gamma_{gs} X_{gsdi} + u_{gsd} + \epsilon_{gsdi}, \qquad (3)$$

$$g \in \{H, M, R\}, s \in \{S\}, d \in \{D\}, u_{gsd} \sim N\left(0, \delta_{u_{gs}}\right), \epsilon_{gsdi} \sim N\left(0, \delta_{\epsilon_{gs}}\right), \beta_{gsd} \sim N\left(\beta_{gs}, \delta_{\beta_{gs}}\right)$$

The index g stands for the three groups with different migrant status while the index s stands for the different specifications that are chosen with $S = \{s_1, s_2, \ldots, s_k\}$ being the set of all $k = 1887$ converged specifications reported in our study (for a graphical representation of all results per group, consult Appendix Figures S4-6). Index i stands for the individual. SI_{gsd} is the social isolation indicator. β_{gsd} is the parameter of interest that we will compare across the three groups to evaluate the five hypotheses. X_{gsd} is a matrix of control variables, and γ_{gsd} is the corresponding vector of coefficients.

One index needs to be mentioned separately: d indexes age and gender specific groups. The model is therefore a multilevel model. Individuals nest within 24 gender specific age groups and $u_d$is the random effect for each group with standard deviation $\delta_{u_{gs}}$. ϵ_{gsdi} is the individual specific error-term with standard deviation δ_ϵ. We therefore allow the association of social isolation with loneliness to vary across gender specific age groups. This is important as in a second step the estimates of the migrant and refugee group are

post-stratified and averaged, with the same distribution across gender specific age groups as the host population. The post-stratification procedure accounts for the possibility that differences in the association found in the data could be attributed to the strong differences in age and gender composition of the three samples. Therefore, the hypotheses are evaluated based on these post-stratified parameters from the aforementioned multilevel regression models:

$$\bar{\beta}_{gs} = \frac{\sum_{d=1}^{24} \beta_{gsd} w_{d_H}}{w_{d_H}} \qquad (4)$$

w_d is the number of observations in each of the gender-specific age groups in the host population.

SM 8 - Bayesian Evaluation of Informative Hypotheses (BEIH)

The BEIH framework is designed for a comparative evaluation of competing hypotheses. It is based on a Bayesian approach to statistical modeling and differs in certain respects from the common frequentist approach [15,16]. The general estimation procedure for the posterior distribution of the parameters we use is the Integrated Nested Laplace Approximation (INLA) [17,18] implemented as a package for R (www.r-inla.org).

Our hypotheses imply a ranking of the association strength of the central parameters $\bar{\beta}_{gs}$. In a Bayesian framework we can estimate the probability that such a ranking - and by extension the proposed hypothesis - is supported by model and data. We therefore get $p(H_t|s(Y))$ where s indexes the specification of the data Y and the model as noted above.

The key feature of the BEIH method is to compare the *observed* support $p(H_t|s(Y))$ for the hypothesis from the estimated posterior distribution of the coefficients to the *expected* support $p(H_t)$ for the hypothesis (prior probability). The prior probability is calculated assuming random ordering of the coefficients [19,20]. From the relation of the two probabilities, we get the Bayes factor:

$$BF_{ts} = \frac{p(H_{th}|s(Y))}{p(H_t)} \qquad (5)$$

If the Bayes factor is larger than 1, the hypothesis formulated has more predictive power than given by chance. Otherwise, if the Bayes factor is smaller than 1, the hypothesis is less probable than by chance.

As we test more than two hypotheses against one another, we additionally calculate posterior model probabilities (PMP):

$$PMP(\boldsymbol{H_t}) = \frac{BF_t}{\sum_{t=1}^{2d} BF_t}; t \in 2a, 2b, 2c, 2d \qquad (6)$$

The PMP states how much support one hypothesis receives compared to the overall support that all hypotheses under investigation receive. The range of the PMP is from 0 to 100%. The higher the value, the stronger the support for the hypothesis in question compared to the competing hypotheses [20].

To illustrate the Bayesian Evaluation of Informative Hypotheses (BEIH) and the ranking of the different hypotheses, we use the *increased need hypothesis* (H_{2a}) as an example.

$$\beta_h < \beta_m < \beta_r$$

The hypothesis proposes a ranking of the strength of association between social isolation and loneliness. $ß_h$ represents the parameter for the host population, $ß_m$ the parameter for migrants of the first generation and $ß_r$ the parameter for refugees. In this case, the hypothesis does not propose equal strength of association but a ranking. This is indicated by the equality sign between parameters. Derived from an increased need to have social contacts, the hypothesis postulates the largest association between social isolation and loneliness for the group of refugees, with decreasing association parameters for migrants and refugees.

Applying BEIH we first specify alternative hypotheses with the different rankings proposed between parameters (see Table 1).

Next, every hypothesis possesses a defined prior that is the probability of finding the hypothesis in the data by chance. The more restrictions or inequalities we place between parameters in one hypothesis, the less support we will find in the data by chance. For example, H_3 represents the hypothesis without any rankings between parameters. It accepts all possible inequalities between the coefficients. Consequently, the *contextual relevance* hypothesis receives a prior of one, while the four hypotheses belong to the set of

context moderation hypothesis have priors based on the number of constraints between parameters. The *refugee exceptionalism hypothesis* (H_{2b}) is much more elaborate that H_3, with one restriction in relation to how the association found in the refugee population should be compared against the host- and migrant population. Most restrictions are placed between the hypothesis including two signs of order, such as the increased need hypothesis (H_{2a}). The likelihood to find these hypotheses by chance further decreases. Hence, their prior probabilities are smaller as well.

Applying our regression models on the data using INLA, we retrieve a posterior distribution. From this distribution, it is possible to draw random samples. From a sample of e.g. 100 000 draws we can now ask the question: How often does e.g. hypothesis $\boldsymbol{H_{2a}}$ hold true in our sample from the posterior distribution? We calculate the marginal likelihood of observing one hypothesis and compare it to the expected likelihood given by chance (our prior distribution). Given the observed support for the posterior distribution and the expected support from the prior of the hypotheses, we can calculate the so-called Bayes factor for each hypothesis (BF) [21], a comparison between the actual outcome and expectation by chance. The proportion $\frac{1}{d_t}$ denotes the grade of support from the posterior distribution, t standing for the hypothesis under consideration. After that we compare the hypothesis to the alternative, for instance that there is no ordering (H_1). $\frac{1}{c_t}$ is defined as the proportion of the prior distribution (expectation) that is in agreement with the hypothesis t. The formula to derive the Bayes Factor is:

$$BF_t = \frac{\frac{1}{d_t}}{\frac{1}{c_t}} = \frac{c_t}{d_t} \qquad (7)$$

Table S8: Main results from figure 2 – Association of social isolation with loneliness

SI-coding	Group	Posterior Mean	LL 95%-CI	UL 95%-CI	min. number of SI
1111	host	0.832	0.281	1.741	4
1111	migrant	0.590	-0.250	1.659	4
1111	refugee	0.967	0.207	1.756	4
1110	host	0.529	0.154	0.957	7
1110	migrant	0.275	-0.250	1.053	7
1110	refugee	0.992	0.479	1.591	7
1101	host	0.559	0.021	1.057	7
1101	migrant	0.899	0.137	1.680	7
1101	refugee	0.728	0.238	1.240	7
1100	host	0.385	0.119	0.617	18
1100	migrant	0.356	-0.197	0.870	18
1100	refugee	0.765	0.457	1.085	18
1011	host	0.990	0.673	1.452	9
1011	migrant	0.532	-0.188	1.225	9
1011	refugee	0.618	-0.050	1.313	9
1010	host	0.541	0.344	0.818	20
1010	migrant	0.210	-0.250	0.623	20
1010	refugee	0.703	0.311	1.164	20
1001	host	0.850	0.593	1.156	15
1001	migrant	0.821	0.303	1.342	15
1001	refugee	0.629	0.269	1.000	15
1000	host	0.468	0.333	0.610	55
1000	migrant	0.298	0.014	0.558	55
1000	refugee	0.564	0.335	0.808	55
0111	host	0.628	0.472	0.803	30
0111	migrant	0.428	0.095	0.751	30
0111	refugee	0.692	0.360	1.038	30
0110	host	0.461	0.363	0.566	94
0110	migrant	0.203	-0.004	0.392	94
0110	refugee	0.668	0.452	0.888	94
0101	host	0.655	0.548	0.772	70
0101	migrant	0.297	0.030	0.538	70
0101	refugee	0.560	0.365	0.772	70
0100	host	0.431	0.366	0.498	263
0100	migrant	0.229	0.090	0.357	263
0100	refugee	0.497	0.349	0.666	263
0011	host	0.512	0.394	0.650	97
0011	migrant	0.278	0.081	0.463	97
0011	refugee	0.424	0.232	0.621	97
0010	host	0.402	0.322	0.493	267
0010	migrant	0.149	0.017	0.271	267
0010	refugee	0.410	0.254	0.565	267
0001	host	0.465	0.383	0.557	182
0001	migrant	0.221	0.060	0.368	182
0001	refugee	0.408	0.249	0.580	182
0000	host	0.376	0.315	0.442	435
0000	migrant	0.166	0.053	0.272	435
0000	refugee	0.403	0.271	0.546	435

Note: Posterior mean = Posterior mean of the posterior distribution of the social isolation coefficient.
LL 95%-CI: Lower limit of the 95% credible interval of the posterior distribution of the social isolation coefficient.
UL 95%-CI: Upper limit of the 95% credible interval of the posterior distribution of the social isolation coefficient.

SM 9 – Theoretical considerations: The hypothesis under investigation and their theoretical origin

Introduction

It is uncontested that that there are differences in the prevalence of social isolation and loneliness between migrants, refugees, and host populations. Migrants are more often subject to social isolation than host populations, as their networks in the new environment need to be (re-) established [22,23]. Additionally, they are also prone to experiencing higher rates of loneliness due to cultural differences and language barriers [24,25]. The same result has been found for refugees [24]. Nonetheless, there is also evidence that migrants' level of loneliness diminishes with time spent in the country of destination, approaching loneliness levels of the host population [26]. Whether comparable trends exist for refugees has yet to be established in longitudinal studies. Moving beyond the investigation of the prevalence in social isolation and loneliness, we focus on the association of the two constructs in our study. The economic, legal, and social differences in context motivate our investigation into the question whether there are relevant differences in the way social isolation is associated with loneliness among regular migrants, refugees, and the host population.

We develop five competing hypotheses about the association of social isolation and loneliness when comparing host, migrant, and refugee populations. These hypotheses imply that the three groups might differ in their evaluation of social networks and support given their different economic, legal, and social circumstances (Table 1). To test the hypotheses, we use one of the few available data sets which includes comparable and harmonized data for refugees, migrants, and the host population, the German Socio-Economic Panel Study (SOEP, v.35) (N=25,171). We use a Bayesian Evaluation of Informative Hypotheses

(BEIH) framework to evaluate the hypotheses [20,27], testing the robustness of our results in a multiverse framework [11,12].

Competing hypotheses

H_1: The contextual relevance hypothesis – From an evolutionary perspective, feeling lonely is a warning sign of the human body. It indicates the deviation from a norm of socializing and hence the presence of a potential hazard in being unprotected without social support of other humans [28]. Research suggests that this mechanism has been established relatively early in human history and has coined the structure of the human brain to be sensitive to feelings of loneliness [28–30]. Hence, from a perspective of evolution we would expect susceptibility to loneliness in all human cultures and conditions. Further, a stronger version of this hypothesis – given a non-clinical, non-institutionalized context - would expect social isolation to predict loneliness to a similar degree, regardless of the context, and in consequence also regardless of migration background. This focus on commonalities between migration groups could be dubbed the evolutionary dominance hypothesis. Given that social circumstances and exposure to prior (possibly critical or traumatic) experiences vary greatly between migrants, refugees, and host population, we propose the competing contextual relevance hypothesis. Thus, stressing the differences between the groups, we expect differences in the association of social isolation and loneliness between host, migrant and refugee population to be of substantive size. In detail, we expect the maximum difference between the associations to be above a threshold of 0.2 standard deviations (for a more detailed discussion of the choice of this value, see SM 4 in the supplemental material).

Expanding on the contextual relevance hypothesis, we propose four hypotheses that make competing predictions the differences in the association between social isolation and loneliness.

H_{2a}: The increased need hypothesis – The post-migration phase requires new skills and knowledge to fully participate in society. Social networks are an important structure, and have the potential to aid integration of migrants [31] and refugees [32,33]. Given the peculiarity of the displacement experience, refugees tend to suffer even stronger resource losses, including income and property loss, expenses of the displacement, physical and mental strain during migration as well as loss of social contact and trust in neighbors, colleagues, and family [34–37]. Given the higher demand for social inclusion and support among refugees due to resource loss, the consequences of objective social isolation should weigh more strongly in perception on refugees. We hence expect the association of social isolation with loneliness to be strongest among refugees, and weaker for other migrants. It is supposedly weakest for the host population who on average have the lowest need to substitute resources.

H_{2b}: The refugee exceptionalism hypothesis – Alternatively to H_{2a} but in a similar line of argument, it can be hypothesized that the differences between the three groups is not gradual in nature, but categorical. Refugees face a more difficult situation in the host country regarding social, cultural, and legal integration. The involuntary disruption of social networks is fundamentally different from that of other migrants and the host population. This unnatural break from social resources sets refugees apart with respect to their vulnerability and hence a need to receive support. Violence of the past remains visible in the aftermath of refugee migration, for instance manifesting in post-traumatic stress disorder [36,38]. Moreover, refugees experience involuntary family separations, entailing fear of family members remaining in danger [39–43].

Finally, refugee housing further isolates the newcomers from the host population and other migrants, with an effect on refugee mental health [44]. A functioning social network, in quantity but also in quality support, is valuable in this context specific strain [45,46]. The lack of social resources under these excluding circumstances might lead to an increased emotional response to the externally induced social isolation. Hence, we expect the association between social isolation and loneliness to be strongest for refugees, with no systematic differences between host and migrant population.

H_{2c}: The numbing hypothesis – This hypothesis makes the opposite prediction to H_{2b}. It is based on the insight that refugees have a higher risk of suffering from Post-Traumatic Stress Disorder (PTSD) and depression due to the extreme circumstances amid their resettlement [47]. Psychological responses to trauma can include a series of bodily reactions such as depersonalization and derealization symptoms [48]. One of them, involuntary in nature, is emotional numbing (Fazel et al., 2005; Spahic-Mihajlovic, Crayton, & Neafsey, 2005). Numbing is a diminished affective responsiveness towards any kind of feelings one might have in an emotional situation, also connected to emotion suppression [50]. We hypothesize that numbing also affects the reaction of refugees towards experiences of social isolation. In the refugee situation, numbing means the dampening of their perception of loneliness. We therefore expect a lesser association between social isolation and loneliness among refugees compared to the host and migrant population.

H_{2d}: The anticipation hypothesis – No matter whether consulting economic theory [51–54], health research [55,56], or insights on social networks [54,57–59], it appears that migrants and refugees moving to another country systematically differ from those they leave behind. Though not fully conclusive, previous studies show that migrants are healthier and more socially connected than the average person in their country

of origin – an indicator for self-selection. They actively consider the opportunities and opportunity costs. For refugees, the trade-off is even stronger due to the nature of the migration process. Both groups might more readily come to terms with insufficient networks for the time being. Based on these assumptions about anticipation of reduced social connections in the post-migration phase, the last hypothesis postulates that migrants show a lower association between social isolation and loneliness than the host population. We expect refugees to show the weakest association of the three groups.

Bibliography

1. McNeish D, An J, Hancock GR. The Thorny Relation Between Measurement Quality and Fit Index Cutoffs in Latent Variable Models. J Pers Assess. Routledge; 2018;100:43–52.

2. Cheung GW, Rensvold RB. Evaluating goodness-of-fit indexes for testing measurement invariance. Struct Equ Model. Lawrence Erlbaum Associates, Inc. ; 2002;9:233–55.

3. Putnick DL, Bornstein MH. Measurement invariance conventions and reporting: The state of the art and future directions for psychological research. Dev Rev. Academic Press; 2016;41:71–90.

4. Siedler T, Schupp J, Spiess CK, Wagner GG. The German Socio-Economic Panel (SOEP) as Reference Data Set. Schmollers Jahrb J Appl Soc Sci Stud für Wirtschafts-und Sozialwissenschaften. 2009;129:367–74.

5. SOEP Group. SOEP-Core – 2016: Individual and Biography (M3/M4, CAPI, with Reference to Variables). SOEP Surv Pap Ser A. Berlin; 2019;656.

6. Hughes ME, Waite LJ, Hawkley LC, Cacioppo JT. A short scale for measuring loneliness in large surveys: Results from two population-based studies. Res. Aging. NIH Public Access; 2004. p. 655–72.

7. DESTATIS. Migration Background Definition. 2020.

8. Baumeister RF, Leary MR. The Need to Belong: Desire for Interpersonal Attachments as a Fundamental Human Motivation. Psychol Bull. 1995;117:497–529.

9. Cornwell EY, Waite LJ. Social disconnectedness, perceived isolation, and health among older adults. J Health Soc Behav. 2009;50:31–48.

10. Luhmann M, Hawkley L. Age differences in loneliness from late adolescence to oldest old age. Dev Psychol. 2016;52:943–59.

11. Simonsohn U, Simmons JP, Nelson LD. Specification curve: Descriptive and inferential statistics on all reasonable specifications. Available SSRN 2694998. 2019;

12. Steegen S, Tuerlinckx F, Gelman A, Vanpaemel W. Increasing Transparency Through a Multiverse Analysis. Perspect Psychol Sci. SAGE PublicationsSage CA: Los Angeles, CA; 2016;11:702–12.

13. Orben A, Dienlin T, Przybylski AK. Social media's enduring effect on adolescent life satisfaction. Proc Natl Acad Sci U S A. National Academy of Sciences; 2019;116:10226–8.

14. Rohrer JM, Egloff B, Schmukle SC. Probing Birth-Order Effects on Narrow Traits Using Specification-Curve Analysis. Psychol Sci. SAGE Publications Inc.; 2017;28:1821–32.

15. Fennessey J. Improving inference for social research and social policy: The Bayesian paradigm. Soc Sci Res. 1977;6:309–27.

16. Gelman A, Carlin JB, Stern HS, Rubin DB. Bayesian Data Analysis. 3rd ed. CRC Press; 2014.

17. Martins TG, Simpson D, Lindgren F, Rue H. Bayesian computing with INLA: new features. Comput Stat Data Anal. 2013;67:68–83.

18. Rue H, Martino S, Chopin N. Approximate Bayesian inference for latent Gaussian models by using integrated nested Laplace approximations. J R Stat Soc Ser B (Statistical Methodol. 2009;71:319–92.

19. Hoijtink H, Klugkist I, Boelen PA. Bayesian evaluation of informative hypotheses. Springer; 2008.

20. van de Schoot R, Verhoeven M, Hoijtink H. Bayesian evaluation of informative hypotheses in SEM using Mplus: A black bear story. Eur J Dev Psychol. 2013;10:81–98.

21. Klugkist I. Encompassing Prior Based Model Selection for Inequality Constrained Analysis of Variance. In: Hoijtink H, Klugkist I, Boelen PA, editors. Bayesian Eval Inf hypotheses. New York: Springer; 2008. p. 53–84.

22. Koelet S, de Valk HAG. Social networks and feelings of social loneliness after migration: The case of European migrants with a native partner in Belgium. Ethnicities. SAGE Publications Ltd; 2016;16:610–30.

23. Ten Kate RLF, Bilecen B, Steverink N, Castle NG. A Closer Look at Loneliness: Why Do First-

Generation Migrants Feel More Lonely Than Their Native Dutch Counterparts? Gerontologist. 2020;60:291–301.

24. De Jong Gierveld J, Van Der Pas S, Keating N. Loneliness of Older Immigrant Groups in Canada: Effects of Ethnic-Cultural Background. J Cross Cult Gerontol. 2015;30:251–68.

25. Lim MH, Eres R, Vasan S. Understanding loneliness in the twenty-first century: an update on correlates, risk factors, and potential solutions. Soc Psychiatry Psychiatr Epidemiol. Springer; 2020;55:793–810.

26. Dolberg P, Shiovitz-Ezra S, Ayalon L. Migration and changes in loneliness over a 4-year period: the case of older former Soviet Union immigrants in Israel. Eur J Ageing. Springer Verlag; 2016;13:287–97.

27. Klugkist I, Mulder J. Bayesian Estimation for Inequality Constrained Analysis of Variance. In: Hoijtink H, Klugkist I, Boelen PA, editors. Bayesian Eval Inf hypotheses. New York: Springer; 2008. p. 27–52.

28. Cacioppo JT, Patrick W. Loneliness : human nature and the need for social connection. W.W. Norton & Co; 2008.

29. Cacioppo S, Bangee M, Balogh S, Cardenas-Iniguez C, Qualter P, Cacioppo JT. Loneliness and implicit attention to social threat: A high-performance electrical neuroimaging study. Cogn Neurosci [Internet]. Psychology Press Ltd; 2016 [cited 2020 Apr 10];7:138–59. Available from: http://www.ncbi.nlm.nih.gov/pubmed/26274315

30. Hawkley LC, Capitanio JP. Perceived social isolation, evolutionary fitness and health outcomes: A lifespan approach [Internet]. Philos. Trans. R. Soc. B Biol. Sci. Royal Society of London; 2015 [cited 2020 Apr 10]. Available from: http://www.ncbi.nlm.nih.gov/pubmed/25870400

31. Gërxhani K, Kosyakova Y. The Effect of Social Networks on Migrants' Labor Market Integration: A Natural Experiment. IAB Discuss Pap. 2020;

32. Månsson J, Delander L. Mentoring as a way of integrating refugees into the labour market—Evidence from a Swedish pilot scheme. Econ Anal Policy [Internet]. 2017 [cited 2019 May 13];56:51–9. Available from: https://linkinghub.elsevier.com/retrieve/pii/S0313592617301613

33. Cheung SY, Phillimore J. Social networks, social capital and refugee integration [Internet]. 2013. Available from: https://www.nuffieldfoundation.org/sites/default/files/files/Phillimore Refugree Integration Report.pdf

34. Ryan D, Dooley B, Benson C. Theoretical perspectives on post-migration adaptation and psychological well-being among refugees: Towards a resource-based model. J Refug Stud [Internet]. Routledge, London; 2008 [cited 2017 Jul 18];21:1–18. Available from: https://academic.oup.com/jrs/article-lookup/doi/10.1093/jrs/fem047

35. Porter M, Haslam N. Predisplacement and postdisplacement factors associated with mental health of refugees and internally displaced persons: A meta-analysis. JAMA. 2005;294:602–12.

36. Schweitzer R, Melville F, Steel Z, Lacherez P. Trauma, post-migration living difficulties, and social support as predictors of psychological adjustment in resettled Sudanese refugees. Aust N Z J Psychiatry. 2006;40:179–87.

37. Steel Z, Silove D, Phan T, Auman A. Long-term effect of psychological trauma on the mental health of Vietnamese refugees resettled in Australia: a population-based study. Lancet. 2002;360:1056–62.

38. Silove D, Sinnerbrink I, Field A, Manicavasgar V, Steel Z. Anxiety, depressionand PTSD in asylum-seekers: assocations with pre-migration trauma and post-migration stressors. Br J Psychiatry. 1997;170:351–7.

39. Hutchinson M, Dorsett P. What does the literature say about resilience in refugee people? Implications for practice. J Soc Incl. 2012;3.

40. Choummanivong C, Poole GE, Cooper A. Refugee family reunification and mental health in resettlement. Kotuitui. Taylor and Francis Inc.; 2014;9:89–100.

41. Löbel LM. Family separation and refugee mental health–A network perspective. Soc Networks. Elsevier B.V.; 2020;61:20–33.

42. Savic M, Chur-Hansen A, Mahmood MA, Moore V. Separation from family and its impact on the mental health of Sudanese refugees in Australia: a qualitative study At-Risk Populations. Aust N Z J Public Health. 2013;37:383–8.

43. Nickerson A, Bryant RA, Steel Z, Silove D, Brooks R. The impact of fear for family on mental health in a resettled Iraqi refugee community. J Psychiatr Res. Pergamon; 2010;44:229–35.

44. Walther L, Kröger H, Tibubos AN, Ta TMT, Von Scheve C, Schupp J, et al. Psychological distress among refugees in Germany: A cross-sectional analysis of individual and contextual risk factors and potential consequences for integration using a nationally representative survey. BMJ Open. BMJ Publishing Group; 2020. p. e033658.

45. Thoits PA. Mechanisms Linking Social Ties and Support to Physical and Mental Health. J Health Soc Behav. 2011;52:145–61.

46. Berkman LF, Glass T, Brissette I, Seeman TE. From social integration to health: Durkheim in the new millennium. Soc Sci Med. 2000;51:843–57.

47. Fazel M, Wheeler J, Danesh J. Prevalence of serious mental disorder in 7000 refugees resettled in western countries: a systematic review. Lancet. 2005;365:1309–14.

48. Sierra M, David AS. Depersonalization: A selective impairment of self-awareness. Conscious Cogn [Internet]. 2011 [cited 2020 Mar 18];20:99–108. Available from: http://www.ncbi.nlm.nih.gov/pubmed/21087873

49. Spahic-Mihajlovic A, Crayton JW, Neafsey EJ. Selective numbing and hyperarousal in male and female Bosnian refugees with PTSD. J Anxiety Disord. Elsevier Ltd; 2005;19:383–402.

50. Tibubos AN, Grammes J, Beutel ME, Michal M, Schmutzer G, Brähler E. Emotion regulation

strategies moderate the relationship of fatigue with depersonalization and derealization symptoms. J Affect Disord. Elsevier B.V.; 2018;227:571–9.

51. Borjas GJ, Bronars SG, Trejo SJ. Self-selection and internal migration in the United States. J Urban Econ. 1992;32:159–85.

52. Borjas GJ, Kauppinen I, Poutvaara P. Self-selection of emigrants: Theory and evidence on stochastic dominance in observable and unobservable characteristics. Econ J. 2019;129:143–71.

53. Aksoy CG, Poutvaara P. Refugees' Self-Selection into Europe: Who Migrates Where? ifo Work Pap Ser No 289. 2019;

54. Mckenzie D, Rapoport H. Self-selection patterns in Mexico-U.S. migration: The role of migration networks 1 [Internet]. 2006. Available from: http://www.migrationint.com.au/ruralnews/guam/jul_2002-15rmn.asp

55. Silventoinen K, Hammar N, Hedlund E, Koskenvuo M, Ronnemaa T, Kaprio J. Selective international migration by social position, health behaviour and personality. Eur J Public Health. 2007;18:150–5.

56. Rubalcava LN, Teruel GM, Thomas D, Goldman N. The Healthy Migrant Effect: New Findings From the Mexican Family Life Survey. Am J Public Health. 2008;98:78–84.

57. Blumenstock J, Chi G, Tan X. Migration and the Value of Social Networks. CEPR Discuss Pap. 2019;13611.

58. Boyd M. Family and personal networks in international migration: Recent developments and new family and personal networks. Int Migr Rev. 1989;23:638–70.

59. Batista C, McIndoe-Calder T, Vicente PC. Return Migration, Self-selection and Entrepreneurship. Oxf Bull Econ Stat. 2017;79:797–821.
